# Asymptotic Robustness Study of the Polychoric Correlation Estimation

**DOI:** 10.1007/s11336-016-9512-2

**Published:** 2016-09-22

**Authors:** Shaobo Jin, Fan Yang-Wallentin

**Affiliations:** Department of Statistics, Uppsala University, 751 20 Uppsala, Sweden

**Keywords:** underlying distribution, asymptotic covariance matrix, non-normality, pseudo-maximum likelihood

## Abstract

Asymptotic robustness against misspecification of the underlying distribution for the polychoric correlation estimation is studied. The asymptotic normality of the pseudo-maximum likelihood estimator is derived using the two-step estimation procedure. The *t* distribution assumption and the skew-normal distribution assumption are used as alternatives to the normal distribution assumption in a numerical study. The numerical results show that the underlying normal distribution can be substantially biased, even though skewness and kurtosis are not large. The skew-normal assumption generally produces a lower bias than the normal assumption. Thus, it is worth using a non-normal distributional assumption if the normal assumption is dubious.

**Electronic supplementary material:**

The online version of this article (doi:10.1007/s11336-016-9512-2) contains supplementary material, which is available to authorized users.

## Introduction

Structural equation models (SEMs) are widely used in social sciences to model latent structures. Typically, normal distributions are assumed for both latent variables and error terms. However, observed measures in surveys are often ordinal. For example, a five-point Likert scale is commonly used in psychometric studies. Conceptually, categorical data should not be incorporated into a SEM by assuming they are continuous. There have been numerous advances in the literature on SEMs with respect to analysing ordinal data as they are. The observed ordinal data are usually assumed to be counterparts of some underlying continuous distributions. A typical choice of the underlying distributions is the standard normal distribution. Olsson (1979) studied the one-step maximum likelihood estimator (MLE) and the two-step MLE of the polychoric correlation coefficient. All parameters (i.e. thresholds and polychoric correlation) are estimated simultaneously for the one-step MLE, whereas the thresholds are estimated from the marginals and the polychoric correlation is computed based on the threshold estimates for the two-step MLE. Olsson showed that under the normality assumption, the one- and the two-step MLEs produce similar polychoric correlation estimates and similar variance estimates. Jöreskog (1994) derived the estimator of the asymptotic covariance matrix of the polychoric correlation estimators for the two-step maximum likelihood procedure (for a more compact expression, see Christoffersson & Gunsjö, 1996, and related references).

The underlying normality assumption is questionable. For example, the underlying normality assumption in the Life Orientation Test dataset (Scheier & Carver, 1985) was rejected by Maydeu-Olivares (2006). In yet another example, income is commonly used in the socio-economic status studies (e.g. Chateau, Metge, Prior, & Soodeen, 2012; Hodge & Treiman, 1968; Scharoun-Lee, Adair, Kaufman, & Gordon-Larsen, 2009). A Pareto distribution is classically used to model income (Arnold, 2008). Using a normal distribution to model income is dubious because the income is bounded by a lower limit. The question regarding income, however, is commonly categorized in a questionnaire: for example, see the National Longitudinal Study of Adolescent Health dataset (Carolina Population Center, 2009) used by Scharoun-Lee et al. (2009). Thus, “income" is an ordinal indicator with a non-normal underlying distribution. The consequences of violating the underlying normality assumption have been investigated (e.g. Flora & Curran, 2004; Lee & Lam, 1988; Quiroga, 1992). Flora and Curran (2004) generated non-normal data from the Fleishman–Vale–Maurelli method (Fleishman, 1978; Vale & Maurelli, 1983) in which a standard univariate normal random variable is polynomially transformed to introduce skewness and kurtosis. The authors found that the polychoric correlation estimates are only slightly biased when the underlying distribution has a skewness of 0.75 or 1.25 and a kurtosis of 1.75 or 3.75. They found, however, that the polychoric correlation is not robust against extreme underlying non-normality (e.g. skewness = 5 and kurtosis = 50). Lee and Lam (1988) generated non-normal data from an elliptical *t* distribution and an elliptical contaminated normal distribution and noted that the polychoric correlation estimates based on the normality assumption are fairly robust against non-normal underlying distributions. The study of Quiroga (1992) was conducted using non-normal data from an underlying bivariate skew-normal distribution and from the Fleishman–Vale–Maurelli method. The author also suggests that the polychoric correlation estimator is robust to non-normality. These studies share two features in common. First, they assume that the underlying distribution is normal to investigate the effect of underlying non-normality. So, a non-normal distribution assumption has not been systematically studied. Second, they are simulation studies. To our knowledge, there are no robustness studies on polychoric correlations from a theoretical standpoint.

Because the polychoric correlation is not distribution-free, tests of the underlying normality assumption are desired. For example, LISREL (Jöreskog & Sörbom, 1996) uses a likelihood ratio test to assess underlying normality, which is equivalent to a Pearson $\chi ^2$. Maydeu-Olivares, Forero, Gallardo-Pujol, and Renom (2009) and Maydeu-Olivares and Joe (2005, 2006) introduced a variant of the Pearson’s $\chi ^2$ that is more suitable for the two-step MLE of the polychoric correlation. LISREL (Jöreskog & Sörbom, 1996) also provides the root-mean-square error of approximation (RMSEA) to assess the underlying normality assumption.

If the normality assumption fails, a new assumption of distribution is needed. Quiroga (1992) studied a new underlying distributional assumption whose marginal distributions are weighted averages of a univariate skew-normal distribution and a standard univariate normal distribution. Through an empirical example, the author showed that the polychoric correlation estimates based on the new assumption of distribution produce a smaller $\chi ^2$ test statistic. The normality assumption has also been criticized in the item response theory and alternative distributions have been studied to account for the underlying non-normality (e.g. see Bolfarine & Bazán, 2010; Lucke, 2014; Woods & Thissen, 2006).

The purpose of this paper is twofold. First, we study robustness against misspecification of the underlying distribution from a theoretical perspective. The effect of distributional misspecification under the two-step maximum likelihood procedure is investigated. Because the two-step MLE is computationally easier (Olsson, 1979) and is implemented in LISREL, we focus only on the two-step MLE for its simplicity and popularity. Second, the underlying distribution is not restricted to a standard normal distribution. The *t* distribution and the skew-normal distribution are used as alternatives in the present study. In particular, the skew-normal distribution has been applied in the item response theory as an alternative to the normality assumption (e.g. see Azevedo, Bolfarine, & Andrade, 2011; Bázan, Branco, & Bolfarine, 2006; Molenaar, 2015; Molenaar, Dolan, & de Boeck, 2012; Santos, Azevedo, & Bolfarine, 2013). Because the underlying distribution cannot be fully determined from ordinal data, we attempt to pinpoint potential alternatives for the bivariate normal distribution assumption.

The remainder of this paper is organized as follows. General theories are presented, followed by numerical examples to illustrate our ideas. A brief conclusion ends the paper.

## General Theory

Consider two ordinal variables *U* and *V* with $m_{U}$ and $m_{V}$ categories, respectively. The classic polychoric correlation estimation method assumes that there are two underlying continuous variables *X* and *Y* for *U* and *V*, respectively. The values of *U* and *V* are defined through *X* and *Y* as

$$\begin{aligned} U=i&\Leftrightarrow \tau _{i-1}<X\le \tau _{i}\ \ i=1,2,\ldots ,m_{U},\\ V=j&\Leftrightarrow \xi _{j-1}<Y\le \xi _{j}\ \ j=1,2,\ldots ,m_{V}, \end{aligned}$$

where $\varvec{\tau }=(\tau _{1},\ldots ,\tau _{m_{U}-1})'$ and $\varvec{\xi }=(\xi _{1},\ldots ,\xi _{m_{V}-1})'$ are thresholds such that

$$\begin{aligned} -\infty =\tau _{0}<\tau _{1}<\cdots<\tau _{m_{U}-1}<\tau _{m_{U}}=\infty ,\\ -\infty =\xi _{0}<\xi _{1}<\cdots<\xi _{m_{V}-1}<\xi _{m_{V}}=\infty . \end{aligned}$$

The true joint distribution function is denoted by $\text {F}(x,y;\rho ,\varvec{\zeta })$ with two marginal distributions $\text {F}_{1}(x)$ and $\text {F}_{2}(y)$, where $\rho$ is the correlation coefficient and $\varvec{\zeta }$ is the vector of other parameters (e.g. degrees of freedom, location, and scale parameters). The corresponding joint density function is $\text {f}(x,y;\rho )$ with marginal densities $\text {f}_{1}(x)$ and $\text {f}_{2}(y)$. Because the true distribution family is unknown, we assume the underlying distribution to be $\text {H}(x,y;\rho )$ with marginal distributions $\text {H}_{1}(x)$ and $\text {H}_{2}(y)$. The joint density function is $\text {h}(x,y;\rho )$ with marginal densities $\text {h}_{1}(x)$ and $\text {h}_{2}(y)$, respectively. Conventionally, $\text {H}(x,y;\rho )$ is taken to be the distribution function of a standard bivariate normal distribution. The normality assumption will be relaxed in our study. We also allow for different marginal distributions both in true underlying distributions and in the assumed ones.

### Two-Step Estimation

#### Threshold Estimation

Let $n_{ij}$ and $p_{ij}$ be the observed frequency and proportion, respectively, of $U=i$ and $V=j$, for $i=1,\ldots ,m_{U}$ and $j=1,\ldots ,m_{V}$. If the true underlying distribution $\text {F}$ is different from the assumed distribution $\text {H}$, the MLEs of thresholds will be inconsistent estimators of $\varvec{\tau }_{0}=(\tau _{1,0},\ldots ,\tau _{m_{U}-1,0})'$ and $\varvec{\xi }_{0}=(\xi _{1,0},\ldots ,\xi _{m_{V}-1,0})'$, where the subscript 0 indicates true values. Consider the ordinal variable *U* first. Denote $\varvec{n}_{U}=(n_{1\cdot },\ldots ,n_{m_{U}\cdot })'$, where $n_{i \cdot }=\sum _{j=1}^{m_V}n_{ij}$ is the marginal total for $i=1,2,\ldots ,m_U$. The corresponding marginal proportion is $\varvec{p}_{U}=(p_{1\cdot },\ldots ,p_{m_{U}\cdot })'$. The pseudo-maximum likelihood estimator (PMLE) of $\varvec{\tau }$, denoted as $\hat{\varvec{\tau }}=(\hat{\tau }_{1},\ldots ,\hat{\tau }_{m_{U}-1})'$, is obtained by maximizing

$$\begin{aligned} \textit{Q}(\varvec{\tau }) = \sum _{i=1}^{m_{U}} n_{i\cdot } \log \intop _{\tau _{i-1}}^{\tau _{i}}\text {h}_1(x)\mathrm{d}x \,. \end{aligned}$$

It is easy to see that $\hat{\varvec{\tau }}$ is a consistent estimator of $\varvec{\tau }^{*}$, where $\text {H}_1 \left( \varvec{\tau }^{*} \right) =\text {F}_{1}(\varvec{\tau }_{0})$, because the observed cell probabilities are consistent estimators of $\text {F}_{1}(\varvec{\tau }_{0})$. Similarly, $\hat{\varvec{\xi }}$ is a consistent estimator of $\varvec{\xi }^{*}$, where $\text {H}_2 \left( \varvec{\xi }^{*} \right) =\text {F}_{2}(\varvec{\xi }_{0})$. Let $\varvec{P}$ be an $m_{U}\times m_{V}$ matrix with (*i*, *j*)-th entry $p_{ij}$. Then

$$\begin{aligned} \frac{\partial \text {Q} \left( \varvec{\tau } \right) }{\partial \varvec{\tau }}&= n\varvec{B}_U \left( \varvec{\tau } \right) '\varvec{D}_U^{-1} \left( \varvec{\tau } \right) \varvec{p}_{U}, \\ \frac{\partial ^2 \text {Q} \left( \varvec{\tau } \right) }{\partial \varvec{\tau }\partial \varvec{\tau }'}&= -n \varvec{B}_U \left( \varvec{\tau } \right) '\varvec{D}_U^{-1} \left( \varvec{\tau } \right) \varvec{D}_{\varvec{p}}\varvec{D}_U^{-1} \left( \varvec{\tau } \right) \varvec{B}_U \left( \varvec{\tau } \right) + n \varvec{S}, \end{aligned}$$

where $n=\sum _{i=1}^{m_U}\sum _{j=1}^{m_V}n_{ij}$ is the total number of observations,

$$\begin{aligned} \varvec{B}_U(\varvec{\tau })&=\left( \begin{array}{cccc} \text {h}_1(\tau _{1}) &{} 0 &{} \cdots &{} 0\\ -\text {h}_1(\tau _{1}) &{} \text {h}_1(\tau _{2}) &{} \cdots &{} 0\\ 0 &{} -\text {h}_1(\tau _{2}) &{} \cdots &{} 0\\ \vdots &{} \vdots &{} \ddots &{} \vdots \\ 0 &{} 0 &{} \cdots &{} \text {h}_1(\tau _{m_{U}-1})\\ 0 &{} 0 &{} \cdots &{} -\text {h}_1(\tau _{m_{U}-1}) \end{array}\right) , \end{aligned}$$

$\varvec{D}_U(\varvec{\tau })=\text {Diag}\left( \intop _{\tau _{0}}^{\tau _{1}}\text {h}_1(\textit{x})\mathrm{d}{} \textit{x},\ldots ,\intop _{\tau _{m_{U}-1}}^{\tau _{m_{U}}}\text {h}_1(\textit{x})\mathrm{d}{} \textit{x}\right) , \varvec{D}_{\varvec{p}}=\text {Diag}(p_{1\cdot },\ldots ,p_{m_{U}\cdot }), \varvec{p}_{U}=\varvec{P}\varvec{1}_{m_{V}}$ with $\varvec{1}_{m_{V}}$ being an $m_V \times 1$ vector of 1’s, and $\varvec{S}$ is a diagonal matrix with *i*-th element

$$\begin{aligned} \left( \frac{p_{i,\cdot }}{\intop _{\tau _{i-1}}^{\tau _{i}}\text {h}_1(x)\mathrm{d}x}-\frac{p_{i+1,\cdot }}{\intop _{\tau _{i}}^{\tau _{i+1}}\text {h}_1(x)\mathrm{d}x}\right) \frac{\partial \text {h}_{1}(\tau _{i})}{\partial \tau _{i}} \,, \end{aligned}$$

for $i \,=\, 1,\ldots ,m_{U}-1$. The operator $\text {Diag}(\cdot )$ constructs a diagonal matrix using the enclosed vector as diagonal elements. The Taylor expansion of $n^{-1/2} \partial \textit{Q}(\hat{\varvec{\tau }})/\partial \varvec{\tau }$ around $\varvec{\tau }^*$ is

1 $$\begin{aligned} 0 \,=\,&n^{-1/2} \frac{\partial \textit{Q} \left( \hat{\varvec{\tau }} \right) }{\partial \varvec{\tau }} = n^{-1/2}\frac{\partial \textit{Q} \left( \varvec{\tau }^* \right) }{\partial \varvec{\tau }} + n^{-1/2} \frac{\partial ^2 \textit{Q} \left( \tilde{\varvec{\tau }} \right) }{\partial \varvec{\tau } \partial \varvec{\tau }' } \left( \hat{\varvec{\tau }}-\varvec{\tau }^{*} \right) +o_{p}( 1), \end{aligned}$$

where $\tilde{\varvec{\tau }}$ lies between $\hat{\varvec{\tau }}$ and $\varvec{\tau }^*$. Because both $\hat{\varvec{\tau }}$ and $\tilde{\varvec{\tau }}$ are consistent estimators of $\varvec{\tau }^*, n^{-1} \partial ^2 \textit{Q}(\tilde{\varvec{\tau }})/\partial \varvec{\tau } \partial \varvec{\tau }'$ is consistent for $-\varvec{B}_U \left( \varvec{\tau }^{*} \right) '\varvec{D}_U^{-1} \left( \varvec{\tau }^{*} \right) \varvec{B}_U \left( \varvec{\tau }^{*} \right)$. So, Eq. (1) implies

2 $$\begin{aligned} n^{1/2} \left( \hat{\varvec{\tau }}-\varvec{\tau }^{*} \right) \,=\,&n^{1/2} \left[ \varvec{B}_U \left( \varvec{\tau }^{*} \right) '\varvec{D}_U^{-1} \left( \varvec{\tau }^{*} \right) \varvec{B}_U \left( \varvec{\tau }^{*} \right) \right] ^{-1} \varvec{B}_U \left( \varvec{\tau }^{*} \right) '\varvec{D}_U^{-1} \left( \varvec{\tau }^{*} \right) \varvec{p}_U+o_{p}(1). \end{aligned}$$

Similar arguments applying to $\varvec{\xi }$ yield

3 $$\begin{aligned} n^{1/2} \left( \hat{\varvec{\xi }}-\varvec{\xi }^{*} \right) =&n^{1/2} \left[ \varvec{B}_V \left( \varvec{\xi }^{*} \right) '\varvec{D}_V^{-1} \left( \varvec{\xi }^{*} \right) \varvec{B}_V \left( \varvec{\xi }^{*} \right) \right] ^{-1} \varvec{B}_V \left( \varvec{\xi }^{*} \right) '\varvec{D}_V^{-1} \left( \varvec{\xi }^{*} \right) \varvec{p}_V+o_{p}(1), \end{aligned}$$

where $\varvec{p}_V=\varvec{P}'\varvec{1}_{m_{U}}$. Here $\varvec{B}_V$ and $\varvec{D}_V$ are defined by substituting $\text {h}_1$ with $\text {h}_2$ in $\varvec{B}_U$ and $\varvec{D}_U$. The PMLEs $\hat{\varvec{\tau }}$ and $\hat{\varvec{\xi }}$ are inconsistent in the sense that $\varvec{\tau }^{*}$ and $\varvec{\xi }^{*}$ are different from the true values $\varvec{\tau }_{0}$ and $\varvec{\xi }_{0}$.

#### Polychoric Correlation Coefficient Estimation

Under the distributional assumption $\text {H}$, the assumed cell probability is

$$\begin{aligned} \pi _{ij,(\text {H})}={\int }_{\tau _{i-1}}^{\tau _{i}}{\int }_{\xi _{j-1}}^{\xi _{j}}\text {h}(x,y)\mathrm{d}y\mathrm{d}x, \end{aligned}$$

while the true cell probability $\pi _{ij,(\text {F})}$ is obtained by substituting $\text {h}(x,y)$ with $\text {f}(x,y)$. Conditionally on $\hat{\varvec{\tau }}$ and $\hat{\varvec{\xi }}$, the polychoric correlation $\rho$ is estimated by maximizing

$$\begin{aligned} \text {L} \left( \rho ,\hat{\varvec{\tau }},\hat{\varvec{\xi }} \right) =\sum _{i=1}^{m_{U}}\sum _{j=1}^{m_{V}}p_{ij}\log \,\pi _{ij,(\text {H})}. \end{aligned}$$

Theorem 2.2 in White (1982) shows that the PMLE is a consistent estimator that minimizes the Kullback–Leibler information (Kullback & Leibler, 1951) under some regularity conditions, one of which is that the absolute value of $\log \pi _{ij,(\text {H})}$ is dominated by a variable with finite expectation. Such a regularity condition is satisfied if $\pi _{ij,(\text {H})}=0$ implies $\pi _{ij,(\text {F})}=0$ for all (*i*, *j*). Consequently, Theorem 2.2 in White (1982) shows that $\hat{\rho }$ converges to $\rho ^{*}$ that minimizes the Kullback–Leibler information

$$\begin{aligned} \sum _{i=1}^{m_{U}}\sum _{j=1}^{m_{V}} \pi _{ij,(\text {F})} \left( \log \,\pi _{ij,(\text {F})} - \log \,\pi _{ij,(\text {H})} \right) . \end{aligned}$$

##### Theorem 1

Assume $\text {g} \left( \rho ,\varvec{\tau }^{*},\varvec{\xi }^{*} \right) = \sum _{i=1}^{m_{U}}\sum _{j=1}^{m_{V}} \pi _{ij,(\text {F})}\log \,\pi _{ij,(\text {H})}$, as a function of $\rho$, has a unique maximum at $\rho ^*$. If $\pi _{ij,(\text {H})}=0$ implies $\pi _{ij,(\text {F})}=0$ for all (*i*, *j*), then there exists a root $\hat{\rho }$ of the equation

$$\begin{aligned} \frac{\partial }{\partial \rho } \sum _{i=1}^{m_{U}}\sum _{j=1}^{m_{V}}p_{ij}\log \,\pi _{ij,(\text {H})}=0 \end{aligned}$$

such that $\hat{\rho }$ is a consistent estimator of $\rho ^{*}$.

That is, $\hat{\rho }$ is a consistent estimator of $\rho ^{*}$ that minimizes the probabilistic divergence between *H* and *F* (Kullback, 1959) in the sense of the Kullback–Leibler information. This minimized divergence implies similarities of *H* and *F* in terms of cell probabilities.

The assumption in Theorem 1 requires uniqueness of the maximum. In so doing, we rule out all cases with local maxima. If we have several stationary points, we can then only conclude that one of the stationary points minimizes the Kullback–Leibler information.

#### Asymptotic Variance of Polychoric Correlations

Let $\text {L}_{\rho } \left( \rho ,\varvec{\tau },\varvec{\xi } \right)$ denote the first order partial derivative of $\text {L} \left( \rho ,\varvec{\tau },\varvec{\xi } \right)$ with respect to $\rho$. Similar symbols are used to represent other partial derivatives and higher order partial derivatives. $\text {L}_{\rho } \left( \hat{\rho },\hat{\varvec{\tau }},\hat{\varvec{\xi }} \right)$ can be expanded around $\rho ^{*}$ for a sufficiently large *n*,

4 $$\begin{aligned} 0= n^{1/2} \text {L}_{\rho }\left( \hat{\rho },\hat{\varvec{\tau }},\hat{\varvec{\xi }} \right) = \underset{\left( i\right) }{\underbrace{n^{1/2} \text {L}_{\rho }\left( \rho ^{*},\hat{\varvec{\tau }},\hat{\varvec{\xi }} \right) }}+\underset{\left( ii\right) }{\underbrace{ n^{1/2} \left( \hat{\rho }-\rho ^{*}\right) \text {L}_{\rho \rho }\left( \tilde{\rho },\hat{\varvec{\tau }},\hat{\varvec{\xi }} \right) }}+o_{p}(1), \end{aligned}$$

where $\tilde{\rho }$ lies between $\rho ^*$ and $\hat{\rho }$. Term $\left( i\right)$ in Eq. (4) is equivalent to

5 $$\begin{aligned} n^{1/2}\text {L}_{\rho }\left( \rho ^{*},\hat{\varvec{\tau }},\hat{\varvec{\xi }}\right) \,=\,&n^{1/2}\text {L}_{\rho }\left( \rho ^{*},\varvec{\tau }^{*},\varvec{\xi }^{*}\right) + n^{1/2} \text {L}_{\rho \varvec{\tau }} \left( \rho ^{*},\tilde{\varvec{\tau }},\tilde{\varvec{\xi }}\right) '\left( \hat{\varvec{\tau }}-\varvec{\tau }^{*}\right) \nonumber \\&+n^{1/2} \text {L}_{\rho \varvec{\xi }} \left( \rho ^{*},\tilde{\varvec{\tau }},\tilde{\varvec{\xi }}\right) '\left( \hat{\varvec{\xi }}-\varvec{\xi }^{*}\right) +o_{p}(1), \end{aligned}$$

where $\tilde{\varvec{\tau }}$ lies between $\hat{\varvec{\tau }}$ and $\varvec{\tau }^{*}$ and $\tilde{\varvec{\xi }}$ lies between $\hat{\varvec{\xi }}$ and $\varvec{\xi }^{*}$. Hence, if $\pi _{ij,\left( \text {H}\right) }=0$ implies $\pi _{ij,\left( \text {F}\right) }=0$ for all $\left( i,j\right)$ in a neighbourhood of $(\varvec{\tau }^{*},\varvec{\xi }^{*})$ given the correlation $\rho ^{*}, \text {L}_{\rho \varvec{\tau }}\left( \rho ^{*},\tilde{\varvec{\tau }},\tilde{\varvec{\xi }}\right)$ is consistent for $\text {g}_{\rho \varvec{\tau }}\left( \rho ^{*},\varvec{\tau }^{*},\varvec{\xi }^{*}\right)$ and $\text {L}_{\rho \varvec{\xi }}\left( \rho ^{*},\tilde{\varvec{\tau }},\tilde{\varvec{\xi }}\right)$ is consistent for $\text {g}_{\rho \varvec{\xi }}\left( \rho ^{*},\varvec{\tau }^{*},\varvec{\xi }^{*}\right)$. Thus, Eq. (5) is equivalent to

$$\begin{aligned} n^{1/2}\text {L}_{\rho }\left( \rho ^{*},\hat{\varvec{\tau }},\hat{\varvec{\xi }}\right)&=n^{1/2}\text {L}_{\rho }\left( \rho ^{*},\varvec{\tau }^{*},\varvec{\xi }^{*}\right) +n^{1/2} \text {g}_{\rho \varvec{\tau }}\left( \rho ^{*},\varvec{\tau }^{*},\varvec{\xi }^{*}\right) '\left( \hat{\varvec{\tau }}-\varvec{\tau }^{*}\right) \nonumber \\&+n^{1/2} \text {g}_{\rho \varvec{\xi }}\left( \rho ^{*},\varvec{\tau }^{*},\varvec{\xi }^{*}\right) '\left( \hat{\varvec{\xi }}-\varvec{\xi }^{*}\right) +o_{p}(1). \end{aligned}$$

Likewise, Term $\left( ii\right)$ can be written as

$$\begin{aligned} n^{1/2}\left( \hat{\rho }-\rho ^{*}\right) \text {L}_{\rho \rho }\left( \tilde{\rho },\hat{\varvec{\tau }},\hat{\varvec{\xi }}\right) = n^{1/2}\left( \hat{\rho }-\rho ^{*}\right) \text {g}_{\rho \rho }\left( \rho ^{*},\varvec{\tau }^{*},\varvec{\xi }^{*}\right) +o_{p}(1), \end{aligned}$$

provided that $\pi _{ij,\left( \text {H}\right) }=0$ implies $\pi _{ij,\left( \text {F}\right) }=0$ in a neighbourhood of $\left( \rho ^*,\varvec{\tau }^{*},\varvec{\xi }^{*}\right)$. Hence, combining with Eqs. (2) and (3), (4) is equivalent to

$$\begin{aligned} n^{1/2}\left( \hat{\rho }-\rho ^{*}\right) =&-\frac{n^{1/2}}{ \text {g}_{\rho \rho }\left( \rho ^{*},\varvec{\tau }^{*},\varvec{\xi }^{*}\right) } \left\{ \text {L}_{\rho }\left( \rho ^{*},\varvec{\tau }^{*},\varvec{\xi }^{*}\right) +\, \text {g}_{\rho \varvec{\tau }}\left( \rho ^{*},\varvec{\tau }^{*},\varvec{\xi }^{*}\right) ' \left( \hat{\varvec{\tau }}-\varvec{\tau }^{*}\right) \right. \nonumber \\&\quad \left. +\, \text {g}_{\rho \varvec{\xi }}\left( \rho ^{*},\varvec{\tau }^{*},\varvec{\xi }^{*}\right) ' \left( \hat{\varvec{\xi }}-\varvec{\xi }^{*}\right) \right\} +o_{p}\left( 1\right) \nonumber \\ =&- \frac{n^{1/2}}{ \text {g}_{\rho \rho }\left( \rho ^{*},\varvec{\tau }^{*},\varvec{\xi }^{*}\right) } \text {tr} \left( \varvec{\Lambda }'\varvec{P} \right) +o_{p}(1), \end{aligned}$$

where $\varvec{\Lambda }=\varvec{A}+\varvec{E}_{\tau }\text {g}_{\rho \varvec{\tau }}\left( \rho ^{*},\varvec{\tau }^{*},\varvec{\xi }^{*}\right) \varvec{1}_{m_{V}}'+\varvec{1}{}_{m_{U}}\text {g}_{\rho \varvec{\xi }}\left( \rho ^{*},\varvec{\tau }^{*},\varvec{\xi }^{*}\right) '\varvec{E}'_{\xi }$ with $\varvec{A}$ being an $m_U\times m_V$ matrix with $\left( i,j\right)$-th element $\left( \partial \pi _{ij}/\partial \rho \right) /\pi _{ij}, \varvec{E}_{\tau } =\varvec{D}_{\tau }^{-1}\varvec{B}_{\tau }\left( \varvec{B}_{\tau }'\varvec{D}_{\tau }^{-1}\varvec{B}_{\tau }\right) ^{-1}$, and $\varvec{E}_{\xi } =\varvec{D}_{\xi }^{-1}\varvec{B}_{\xi }\left( \varvec{B}_{\xi }'\varvec{D}_{\xi }^{-1}\varvec{B}_{\xi }\right) ^{-1}$. Note that $\text {tr}\left( \varvec{\Lambda }'\varvec{P} \right) = \mathrm {vec}{ (\varvec{\Lambda })}' \mathrm {vec}{(\varvec{P})}$, where $\mathrm {vec}(\cdot )$ stacks the columns of the enclosed matrix and

$$\begin{aligned} \sqrt{n} \left( \mathrm {vec}{(\varvec{P})} - \mathrm {vec}(\varvec{\pi }_{\left( \text {F}\right) }) \right) \overset{d}{\rightarrow } N \left( \varvec{0}, \text {Diag}(\varvec{P}) - \mathrm {vec}{(\varvec{P})} \mathrm {vec}{(\varvec{P})}' \right) \end{aligned}$$

with $\varvec{\pi }_{\left( \text {F}\right) }$ being an $m_{U}\times m_{V}$ matrix with (*i*, *j*)-th entry $\pi _{ij,\left( \text {F}\right) }$. The arguments above establish the following theorem.

##### Theorem 2

Let $\hat{\rho }$ be the consistent root of $\text {L}_{\rho }\left( \rho ,\hat{\varvec{\tau }},\hat{\varvec{\xi }}\right) =0$ given $\hat{\varvec{\tau }}$ and $\hat{\varvec{\xi }}$. Assume $\pi _{ij,\left( \text {H}\right) }=0$ implies $\pi _{ij,\left( \text {F}\right) }=0$ in a neighbourhood of $\left( \rho ^*,\varvec{\tau }^{*},\varvec{\xi }^{*}\right)$, then

$$\begin{aligned} n^{1/2}\left( \hat{\rho }-\rho ^{*}\right)&\overset{d}{\rightarrow }N\left( 0,\sigma ^{2}\right) , \end{aligned}$$

where $\sigma ^{2}=\left[ \text {tr} \left( \left( \varvec{\Lambda }\odot \varvec{\Lambda } \right) '\varvec{\pi }_{\left( \text {F}\right) } \right) -\left( \text {tr}\left( \varvec{\Lambda }'\varvec{\pi }_{\left( \text {F}\right) } \right) \right) ^{2} \right] / \left( \text {g}_{\rho \rho } \left( \rho ^{*},\varvec{\tau }^{*},\varvec{\xi }^{*} \right) \right) ^{2}$. Here, matrix $\varvec{\Lambda }$ is evaluated under $\left( \rho ^{*},\varvec{\tau }^{*},\varvec{\xi }^{*}\right)$. The operator $\odot$ implies element-wise multiplication.

#### Estimating the Asymptotic Covariance Matrix

Following Theorem 2, the asymptotic variance of $\hat{\rho }$ can be consistently estimated by

$$\begin{aligned} \frac{1}{\left[ \text {L}_{\rho \rho }\left( \hat{\rho },\hat{\varvec{\tau }},\hat{\varvec{\xi }}\right) \right] ^{2} } \left[ \sum _{i=1}^{m_U}\sum _{j=1}^{m_V} \hat{\lambda }_{ij}^2 p_{ij} - \left( \sum _{i=1}^{m_U}\sum _{j=1}^{m_V} \hat{\lambda }_{ij} p_{ij} \right) ^2 \right] , \end{aligned}$$

where $\hat{\lambda }_{ij}$ is the (*i*, *j*)-th element in $\hat{\varvec{\Lambda }}$. The polychoric correlation between variables *U* and *V* satisfies

$$\begin{aligned} n^{1/2}\left( \hat{\rho }^{(UV)}-\rho ^{*(UV)}\right)&=\frac{-n^{1/2}}{ \text {g}_{\rho \rho }^{(UV)} \left( \rho ^{*},\varvec{\tau }^{*},\varvec{\xi }^{*} \right) }\text {tr} \left( \varvec{\Lambda }^{\left( UV\right) '}\varvec{P}^{\left( UV\right) } \right) +o_{p}\left( 1\right) , \end{aligned}$$

where the superscript (*UV*) emphasizes that all quantities are evaluated under the distributional assumption for *U* and *V*. Similarly, the polychoric correlation between variables *K* and *Z* satisfies

$$\begin{aligned} n^{1/2}\left( \hat{\rho }^{(KZ)}-\rho ^{*(KZ)}\right)&=\frac{-n^{1/2}}{ \text {g}_{\rho \rho }^{(KZ)} \left( \rho ^{*},\varvec{\tau }^{*},\varvec{\xi }^{*} \right) }\text {tr} \left( \varvec{\Lambda }^{\left( KZ\right) '}\varvec{P}^{\left( KZ\right) } \right) +o_{p}\left( 1\right) . \end{aligned}$$

The underlying distributional assumption for *U* and *V* can either be the same as that for *K* and *Z* or different. Thus, the asymptotic covariance between $\hat{\rho }^{(UV)}$ and $\hat{\rho }^{(KZ)}$ is consistently estimated by

6 $$\begin{aligned} \frac{\sum _{a=1}^{m_{U}}\sum _{b=1}^{m_{V}}\sum _{c=1}^{m_{K}}\sum _{d=1}^{m_{Z}} \hat{\lambda }_{ab}^{\left( UV\right) } \left( p_{abcd}^{\left( UVKZ\right) }-p_{ab}^{\left( UV\right) }p_{cd}^{\left( KZ\right) } \right) \hat{ \lambda }_{cd}^{\left( KZ\right) }}{\text {L}_{\rho \rho }\left( \hat{\rho }^{(UV)},\hat{\varvec{\tau }}^{\left( UV\right) },\hat{\varvec{\xi }}^{\left( UV\right) }\right) \text {L}_{\rho \rho }\left( \hat{\rho }^{(KZ)},\hat{\varvec{\tau }}^{\left( KZ\right) },\hat{\varvec{\xi }}^{\left( KZ\right) }\right) } \,, \end{aligned}$$

where $p_{abcd}^{(UVKZ)}$ is the sample proportion of observing $U=a, V=b, K=c$, and $Z=d$. Under the assumption that the underlying distribution is normal and correctly specified, Eq. (6) reduces to the estimator in Jöreskog (1994).

### A Variant of Two-Step Estimation

The above two-step estimation is applicable to bivariate distributions whose marginal distributions do not depend on unknown parameters. For example, the mean and variance of a bivariate normal distribution are unknown parameters and assuming a standard normal distribution fixes those parameters to known values. In many other distributions, unknown parameters are included in the marginal distributions. Consequently, the above two-step MLE cannot be obtained unless the unknown parameters are prefixed. In such a case, a variant of the two-step MLE can be obtained instead. The MLE maximizes

$$\begin{aligned} L\left( \varvec{\theta } \right)&= \sum _{i=1}^{m_{U}}\sum _{j=1}^{m_{V}}p_{ij}\log \,\pi _{ij,(\text {H})} \end{aligned}$$

with respect to the vector $\varvec{\theta }$ that consists of free unknown parameters. Not all parameters in $\rho$ and $\varvec{\zeta }$ are free parameters. The mean and variance of an ordinal variable are not identified. Thus, the scale and location parameters that do not contribute to the correlation coefficient are not identified. In some distributions (e.g. the skew-normal distribution introduced later), the correlation coefficient is also determined by $\varvec{\zeta }$ and, therefore, is not a free parameter. If $L\left( \varvec{\theta } \right)$ is differentiable with respect to $\varvec{\theta }$,

$$\begin{aligned} \frac{\partial }{\partial \varvec{\theta }}L\left( \varvec{\theta } \right) = \frac{\partial }{\partial \varvec{\theta }} \sum _{i=1}^{m_{U}}\sum _{j=1}^{m_{V}}p_{ij}\log \,\pi _{ij,(\text {H})}=\varvec{0} \end{aligned}$$

is solved to obtain $\hat{\varvec{\theta }}$. By standard calculation,

$$\begin{aligned} n^{1/2} \left( \hat{\varvec{\theta }} - \varvec{\theta }^* \right)&= - n^{1/2} E\left( L_{\varvec{\theta }\varvec{\theta }} ( \varvec{\theta }^*)\right) ^{-1} \varvec{C}( \varvec{\theta }^*) \text {vec} (\varvec{P}) + o_{p}(1), \end{aligned}$$

where $\varvec{\theta }^*= (\rho ^*, \varvec{\zeta }^{*'})'$ and the *k*-th row in $\varvec{C}$ is $\text {vec}(\varvec{C}_k)'$ with the (*i*, *j*)-th element being $\frac{1}{\pi _{ij,(H)}} \frac{\partial \pi _{ij,(H)}}{\partial \varvec{\theta }_k }$, provided that $E\left( L_{\varvec{\theta }\varvec{\theta }} ( \varvec{\theta }^*)\right)$ is invertible. Assume that the correlation coefficient satisfies $\rho =\rho (\varvec{\theta })$ in which $\rho _{\varvec{\theta }} (\varvec{\theta }^*)$ is nonzero. The delta method (Ferguson, 1996) indicates

$$\begin{aligned} n^{1/2} \left( \hat{\rho } - \rho ^* \right) \overset{d}{\rightarrow } N\left( 0, \rho _{\varvec{\theta }}(\varvec{\theta }^*)' E\left( L_{\varvec{\theta }\varvec{\theta }} ( \varvec{\theta }^*)\right) ^{-1} \varvec{C}( \varvec{\theta }^*) \varvec{\Omega } \varvec{C}( \varvec{\theta }^*)' E\left( L_{\varvec{\theta }\varvec{\theta }} ( \varvec{\theta }^*)\right) ^{-1} \rho _{\varvec{\theta }}(\varvec{\theta }^*) \right) , \end{aligned}$$

where $\varvec{\Omega }$ is the asymptotic covariance matrix of $\text {vec} (\varvec{P})$. Hence, the asymptotic covariance between $\hat{\rho }^{(UV)}$ and $\hat{\rho }^{(KZ)}$ can be consistently estimated in a similar manner to Eq. (6). Let the matrix $\varvec{\Upsilon }$ be constructed through $\text {vec} \left( \varvec{\Upsilon } \right) ' = \rho _{\varvec{\theta }} (\varvec{\theta })' E\left( L_{\varvec{\theta }\varvec{\theta }} ( \varvec{\theta })\right) ^{-1} \varvec{C}( \varvec{\theta })$. Then the asymptotic covariance between $\hat{\rho }^{(UV)}$ and $\hat{\rho }^{(KZ)}$ is consistently estimated by

7 $$\begin{aligned} \sum _{a=1}^{m_{U}}\sum _{b=1}^{m_{V}}\sum _{c=1}^{m_{K}}\sum _{d=1}^{m_{Z}} \hat{\varvec{\Upsilon }}_{ab}^{\left( UV\right) } \left( p_{abcd}^{\left( UVKZ\right) }-p_{ab}^{\left( UV\right) }p_{cd}^{\left( KZ\right) } \right) \hat{\varvec{\Upsilon }}_{cd}^{\left( KZ\right) } . \end{aligned}$$

In Olsson (1979), thresholds $\varvec{\tau }$ and $\varvec{\xi }$ are parameters for the one-step MLE. However, the thresholds are not always directly estimated for the one-step MLE for other distributions. For example, the density function of a bivariate skew-normal distribution in Azzalini and Valle (1996) is

8 $$\begin{aligned} \text {f} \left( x,y \right)&= 2\phi _2 \left( x,y; \omega \right) \Phi \left( \alpha _1x+\alpha _2y \right) \,, \end{aligned}$$

where $\phi _2(\cdot ,\cdot ;\omega )$ is the density function of the bivariate standard normal distribution with correlation coefficient $\omega , \Phi (\cdot )$ is the distribution function of a standard normal distribution, and $\alpha _1$ and $\alpha _2$ control skewness and kurtosis. The covariance matrix of *X* and *Y* is

9 $$\begin{aligned} \varvec{W} -\frac{2}{ \pi \left( 1+\alpha _{1}^{2}+2\omega \alpha _{1}\alpha _{2}+\alpha _{2}^{2}\right) } \varvec{R} \,, \end{aligned}$$

where

$$\begin{aligned} \varvec{W} = \left( \begin{array}{cc} 1 &{} \omega \\ \omega &{} 1 \end{array}\right) \quad \text {and} \quad \varvec{R} = \left( \begin{array}{cc} \left( \alpha _{1}+\omega \alpha _{2}\right) ^{2} &{} \left( \alpha _{1}+\omega \alpha _{2}\right) \left( \alpha _{2}+\omega \alpha _{1}\right) \\ \left( \alpha _{1}+\omega \alpha _{2}\right) \left( \alpha _{2}+\omega \alpha _{1}\right) &{} \left( \alpha _{2}+\omega \alpha _{1}\right) ^{2} \end{array}\right) . \end{aligned}$$

Thus, the correlation coefficient is affected by $\omega , \alpha _1$ and $\alpha _2$. The marginal distributions are univariate skew-normal distributions with densities

10 $$\begin{aligned} \text {f}\left( x \right)&= 2 \phi \left( x \right) \Phi \left( \alpha x \right) , \end{aligned}$$

where $\alpha = (\alpha _1+\omega \alpha _2)/\left[ 1+(1-\omega ^2)\alpha _2^2\right] ^{1/2}$ for *X* and $\alpha = (\alpha _2+\omega \alpha _1)/\left[ 1+(1-\omega ^2)\alpha _1^2\right] ^{1/2}$ for *Y*. Bazán et al. (2006), Molenaar (2015), and Molenaar et al. (2012) have applied the univariate skew-normal distribution to the item response theory. The marginal distributions are affected by $\omega , \alpha _1$, and $\alpha _2$, as are the thresholds. Therefore, the thresholds are not free parameters. The vector of free parameters in the variant of two-step estimation is $\varvec{\theta }=(\alpha _1,\alpha _2,\omega )'$.

## Numerical Examples

A numerical study is conducted in this section to examine the asymptotic bias under different distributional assumptions. Asymptotic limits of PMLE for polychoric correlation coefficients are numerically computed.

### Distributional Assumption

Four experiments are conducted in which different true underlying distributions are investigated.

#### Experiment 1: Elliptical Distribution

In probability and statistics, an elliptical distribution belongs to a broad family of probability distributions. The bivariate joint density function of an elliptical distribution is of the form

11 $$\begin{aligned} \frac{1}{2\pi \sigma _{11}\sigma _{22} \left( 1-\rho ^2 \right) ^{1/2} } \text {q} \left( z \right) , \end{aligned}$$

where $\text {q} \left( \cdot \right)$ is a univariate function and

$$\begin{aligned} z = \frac{1}{1-\rho ^{2}}\left[ \frac{(x-\mu _{1})^{2}}{\sigma _{11}^2}-\frac{2\rho (x-\mu _{1})(y-\mu _{2})}{ \sigma _{11}\sigma _{22} }+\frac{(y-\mu _{2})^{2}}{\sigma _{22}^2}\right] \end{aligned}$$

with $\sigma _{11}$ being the variance of *X* and $\sigma _{22}$ being the variance of *Y*. An elliptical distribution generalizes the normal distribution and keeps some properties (e.g. Balakrishnan & Lai, 2009; Fang, Kotz, & Ng, 1990; Kelker, 1970). Some examples of the bivariate elliptical distributions that will be used later are

1. Normal distributions: $$q\left( z \right) = \exp \left( -z/2 \right) $$;
2. *t*(*v*) distributions with degrees of freedom *v*: $q\left( z \right) = \left( 1+z/v \right) ^{-(v+2)/2}$;
3. Bivariate uniform distributions: $q\left( z \right) = 2\text {I}_{ \{z\le 1\} }$ with $\text {I}$ being an indicator function;
4. Bivariate Logistic distributions: $q\left( z \right) = 4\exp \left( -z \right) /\left[ 1+\exp \left( -z \right) \right] ^2$;
5. Bivariate exponential power distributions: $q\left( z \right) = 2\exp \left( -z^{\beta }/2 \right) /\left( 2^{1/\beta } \Gamma (1+1/\beta )\right)$.

The elliptical distribution family plays a very important role in robustness studies (e.g. Kano, Berkane, & Bentler, 1993). In the context of Pearson correlation estimation, Hampel, Ronchetti, Rousseeuw, and Stahel (1986) showed that the PMLE of the covariance matrix is proportional to the MLE under the true distributional assumption, provided that continuous data have been acquired. This result enables us to use any member of the family to estimate the correlation matrix, having the same estimates as if the true distribution were used. Likewise, Berkane, Kano, and Bentler (1994) claimed that “there is practically no cost in treating the distribution as multivariate *t* with specified (possibly small) degrees of freedom” (Berkane et al., 1994, p. 266) when the true distribution is normal and continuous data are observed. It only slightly inflates the variance of the resulting estimator. Thus, it is worth investigating the effect of an underlying elliptical distribution. Because we have only categorical data, the mean and variance are not identified. But then only the correlation coefficient $\rho$ is the parameter of interest, so we can assume $\mu _{1}=\mu _{2}=0$ and $\sigma _{11}=\sigma _{22}=1$.

For some members of the elliptical distribution family, the marginal distribution is still elliptical but not of the same type (Gómez, Gómez-villegas, & Marín, 2003). The bivariate uniform distribution, the logistic distribution, and the exponential power distribution possess such properties. The support of the bivariate uniform distribution is not the whole Cartesian plane, whereas the other distributions have the whole Cartesian plane as their support. The exponential power distribution includes the normal distribution $(\beta =1)$ and the Laplace distribution $(\beta =1/2)$ as special cases.

#### Experiment 2: Skew-Normal Distribution

An elliptical distribution is symmetric. Qui-roga (1992) reported that kurtosis does not have strong effects on the polychoric correlation but that skewness increases the bias. The above elliptical distributions examine various values of kurtosis. The following distributions introduce nonzero values of skewness.

A natural generalization of a standard normal distribution is the univariate skew-normal distribution proposed by Azzalini (1985) and extended by Azzalini and Valle (1996) to a multivariate skew-normal distribution. The bivariate density function, covariance matrix and marginal density function are shown in Eqs. (8), (9), and (10), respectively. The ranges of the skewness and excess kurtosis are $(-0.9953,0.9953)$ and [0, 0.8692), respectively (Azzalini & Capitanio, 2014, p. 32). This range is close to the low skewness and low kurtosis case in Flora and Curran (2004). The reader can refer to Azzalini (2005) for an overview of the skew-normal distribution and to Azzalini and Capitanio (2014) for the expressions of skewness and excess kurtosis. Note that the bivariate skew-normal distribution proposed by Azzalini and Valle (1996) is different from the skew-normal distribution in Quiroga (1992). The specification in Azzalini and Valle (1996) is used in the present study for its connection with the skew-t(*v*) distribution in the next experiment.

#### Experiment 3: Skew-t(*v*) Distribution

Skewness can also be introduced to the t(*v*) distribution. Azzalini and Capitanio (2003) proposed a multivariate skew-*t*(*v*) distribution whose bivariate density function is

$$\begin{aligned} \text {f}\left( x,y \right)&= 2 \text {t} \left( x,y; \omega , v \right) \text {T} \left( (\alpha _1x+\alpha _2y) \left( \frac{v+2}{v+ (x^2 - 2\omega xy + y^2)/(1-\rho ^2)} \right) ^{1/2} ; v+2 \right) , \end{aligned}$$

where $\text {t}(\cdot ,\cdot ; \omega , v)$ is the density function of a standard bivariate *t* distribution with correlation *w* and degrees of freedom *v* and $\text {T}(\cdot ;v+2)$ is the distribution function of a univariate *t* distribution with degrees of freedom $v+2$. The covariance matrix of *X* and *Y* is

$$\begin{aligned} \frac{v}{v-2} \varvec{W} -\frac{2}{\pi \left( 1+\alpha _{1}^{2}+2\omega \alpha _{1}\alpha _{2}+\alpha _{2}^{2}\right) }\varvec{R} \,, \end{aligned}$$

provided that $v>2$. Both marginal distributions are univariate skew-*t* distributions with density function

$$\begin{aligned} \text {f}\left( x \right)&= 2 \text {t} \left( x; v \right) \text {T} \left( \alpha x \left( \frac{v+1}{v+x^2} \right) ^{1/2} ; v+1 \right) . \end{aligned}$$

The reader is directed to Azzalini and Capitanio (2003) for the expressions of skewness and excess kurtosis.

#### Experiment 4: Other Distributions

The skew-normal and t(*v*) distributions are special cases of the skew-t(*v*) distribution family. There are many distributions that are not members of the skew-elliptical distribution family. In addition, the underlying distribution cannot be truly determined from the observed ordinal data. It is therefore important to investigate the effect of distributional misspecification using the distributions that do not belong to the skew-*t* distribution family. A Pareto distribution is commonly used to model income (Arnold, 2008) and income is commonly used as an indicator of socio-economic status. Mardia (1962) proposed a multivariate Pareto distribution in which the bivariate density function is

$$\begin{aligned} \text {f}(x,y;a,\theta _1,\theta _2) = (a+1)a(\theta _1 \theta _2)^{a+1}(\theta _2x + \theta _1y - \theta _1 \theta _2)^{-(a+2)}, \end{aligned}$$

and the marginal density function is $\text {f}(x)=a\theta _i^a x^{-(a+1)}$, with $x \ge \theta _1>0, y \ge \theta _2>0$, and $a>0$. The correlation coefficient between *X* and *Y* is 1 / *a*, which is always positive.

### Numerical Design

Three combinations of categories are used. First, both *U* and *V* have five categories with cell probabilities (0.1, 0.2, 0.4, 0.2, 0.1) and (0.1, 0.1, 0.3, 0.3, 0.2), respectively. Second, both *U* and *V* have three categories with cell probabilities (0.2, 0.5, 0.3) and (0.1, 0.3, 0.6), respectively. Third, *U* has three categories with cell probabilities (0.2, 0.5, 0.3) and *V* has five categories with cell probabilities (0.1, 0.1, 0.3, 0.3, 0.2).

In Experiment 1, $\beta$ in the exponential power distribution is $\beta =0.3,0.4,0.5,0.6$. In Experiments 2 and 3, three values of $\alpha _1$ are considered ($\alpha _1=0.1,0.5,1$) and 20 evenly spaced values of $\alpha _2$ are considered ranging from 0.5 to 10 for both skew-normal and skew-t(*v*) distributions. Thus, different combinations of univariate skewness and kurtosis are investigated. In Experiments 1, 2 and 3, the degrees of freedom for the t(*v*) and skew-t(*v*) distributions are 4, 6, 8, and 10. In Experiment 4, parameters for the Pareto distribution are $\theta _1=\theta _2=3$.

For all experiments, two values of $\rho$ are used: $\rho _{0}=0.4,0.6$. For the purpose of illustration, the assumed underlying distributions are bivariate normal, skew-normal, and t(*v*) distributions. The normal assumption consists of only one unknown parameter of interest, $\rho$. The skew-normal assumption consists of three parameters: $\alpha _1, \alpha _2$, and $\omega$ that determine the correlation coefficient. The degrees of freedom in the t(*v*) are prefixed to be 4, 6, 8, 10 and the correlation coefficient is the only parameter of interest. The expressions of the partial derivatives of the skew-normal distribution and t distribution can be found in the supplementary materials.

### Numerical Results

To assess the bias of polychoric correlation estimates, the relative bias (RB) is computed, which is defined as $RB = 100 \times \left( \hat{\rho }-\rho _0\right) / \rho _0$. Following the definition in Flora and Curran (2004), $RB \le 5, 5< RB \le 10$, and $RB \ge 10$ indicate slight, moderate, and large bias, respectively. To assess the closeness of the fit, the limit value of RMSEA

$$\begin{aligned} RMSEA&= \left( \max \left[ \frac{ 2\sum _{i=1}^{m_{U}}\sum _{j=1}^{m_{V}} \pi _{ij,(\text {F})}\log \left( \pi _{ij,(\text {F})} / \pi _{ij,(\text {H})}\right) }{m_U m_V -m_U - m_V} , 0 \right] \right) ^{1/2}, \end{aligned}$$

is computed. Owing to space limitations, here only some main results are presented and discussed in this subsection. Complete results can be found in the supplementary materials.

#### Experiment 1

**Fig. 1:**
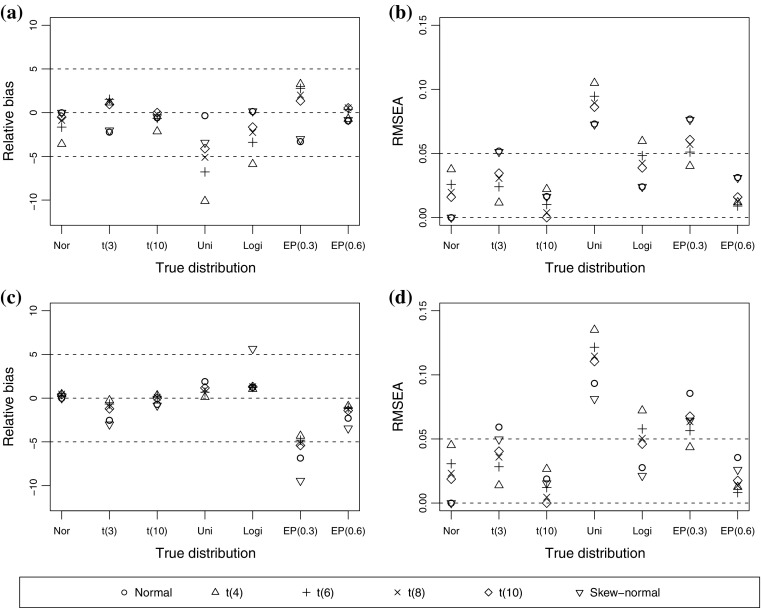
Relative bias (RB) and root-mean-square error of approximation (RMSEA) of correlation estimates when the true underlying distribution belongs to the elliptical distribution family. The true correlation coefficient is 0.4, **a** RB when both ordinal variables have five categories. **b** RMSEA when both ordinal variables have five categories. **c** RB when both ordinal variables have three categories. **d** RMSEA when both ordinal variables have three categories. *Note* *Nor* normal, *Uni* uniform, *Logi* logistic, EP$(\cdot )$=exponential power distribution with the enclosed value of $\beta .$

Figures 1 displays the RB and RMSEA values when the true correlation is 0.4. As expected, assuming a wrong underlying distribution generally biases the polychoric correlation. Observe that the skew-normal distribution contains the normal distribution as a special case. Thus, both the normal and skew-normal assumptions consistently estimate the polychoric correlation when the true underlying distribution is normal. When the true underlying distribution is a normal distribution or a t distribution, all distributional assumptions produce a low RB (less than $5\,\%$). When the true underlying distribution is a uniform distribution or logistic distribution, the normal assumption generally produces a low-biased correlation estimate. However, the t assumption may produce a high RB (Figure 1). The normal and skew-normal assumptions can produce moderately biased polychoric correlations when the underlying distribution is the exponential power distribution with $\beta =0.3$ that corresponds to a distribution with high kurtosis (Figure 1). As the kurtosis in the exponential power family decreases, the magnitude of RB concomitantly decreases. When the underlying distribution is non-normal, the normal and skew-normal assumptions may produce different correlation estimates. Thus, the skew-normal distribution adjusts the underlying non-normality by introducing some degree of skewness. Consequently, the magnitude of RB may become higher but the RMSEA may become lower (Figure 1), which occurs when the number of categories is three for both ordinal variables. The polychoric correlation based on the underlying normal assumption generally underestimates the true correlation coefficient. The *t*(4) and *t*(6) assumptions sometimes outperform the normal assumption in Experiment 1.

#### Experiment 2

**Fig. 2:**
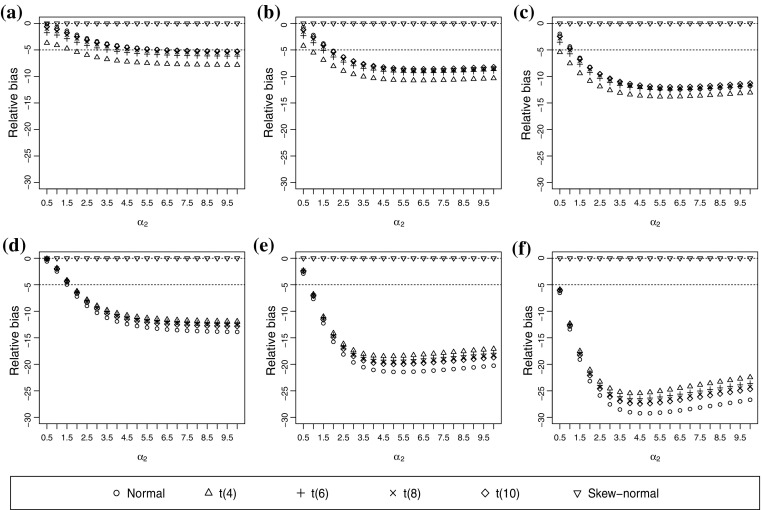
Relative bias (RB) of correlation estimates when the true underlying distribution is skew-normal. The true correlation coefficient is 0.4. **a** $\alpha _1=0.1$ and both ordinal variables have five categories. **b** $\alpha _1=0.5$ and both ordinal variables have five categories. **c** $\alpha _1=1$ and both ordinal variables have five categories. **d** $\alpha _1=0.1$ and both ordinal variables have three categories. **e** $\alpha _1=0.5$ and both ordinal variables have three categories. **f** $\alpha _1=1$ and both ordinal variables have three categories.

As expected, the polychoric correlation is consistently estimated when the true and assumed underlying distributions are both skew-normal (Figure 2). The normal assumption produces negatively biased correlation estimates. It can be moderately or strongly biased unless both $\alpha _1$ and $\alpha _2$ are small. Recall that $\alpha _1$ and $\alpha _2$ control the skewness and kurtosis of the underlying distribution. Small values of $\alpha _1$ and $\alpha _2$ only introduce a small departure from the bivariate normal distribution. All the *t* distribution assumptions produce similar RBs relative to the normal assumption. Under both the normal and t(*v*) distribution assumptions, three categories in both ordinal variables generally lead to a higher magnitude of the RB value than five categories in both variables. For example, the RB with five-category variables does not exceed $-15$ when $\alpha _1=1$ and $\rho _0=0.4$, whereas the RB with three-category variables frequently exceeds $-25$ under the same condition (Figure 2). As the true value of the correlation increases while the other conditions remain the same, the RB generally becomes smaller (see Figures 7, 8, and 9 in the supplementary materials).

**Fig. 3:**
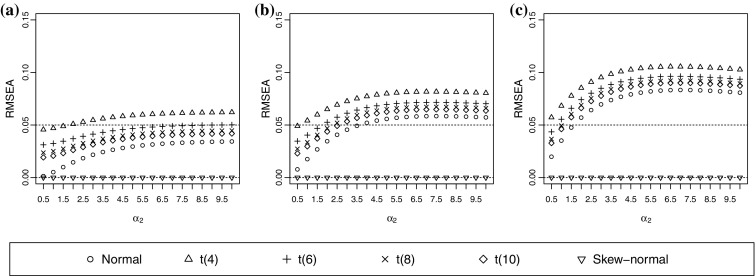
Root-mean-square error of approximation (RMSEA) of correlation estimates when the true underlying distribution is skew-normal. The true correlation coefficient is 0.4. Both ordinal variables have three categories. **a** $\alpha _1=0.1$. **b** $\alpha _1=0.5$. (c) $\alpha _1=1.$

In Experiment 2, the RMSEA can be misleading when the number of categories is three in both variables. Consider the normal assumption as an example. The magnitude of RB may exceed 10 when $\alpha _1=0.1, \rho _0=0.4$, and both ordinal variables have three categories (Figure 2), whereas the RMSEA is still below 0.05 (Figure 3). The pattern is more dramatic when $\alpha _1=1$. The RB is almost $-20$ when $\alpha _2=1.5$ and $\rho _0=0.4$, but the RMSEA is slightly below 0.05. Thus, the estimated probabilities can be rather close to the true probabilities but the polychoric correlation can be largely biased. This event occurs because RMSEA only measures the closeness between the estimated and true category probabilities, and is not a direct measure of the correlation estimate.

On the other hand, although the skew-normal assumption consistently estimates the polychoric correlation in Experiment 2, the numerical difficulties (such as non-convergence and local maximizer) are encountered in the present study. The fit function $L\left( \varvec{\theta } \right)$ can be fairly flat (see Figure 13 in the supplementary materials as an illustration). A bad choice of the starting value for the numerical optimization process can lead to the aforementioned issues. Thus, 20 starting values are employed. As a result, the skew-normal assumption is computationally much more intensive than the normal assumption.

#### Experiment 3

**Fig. 4:**
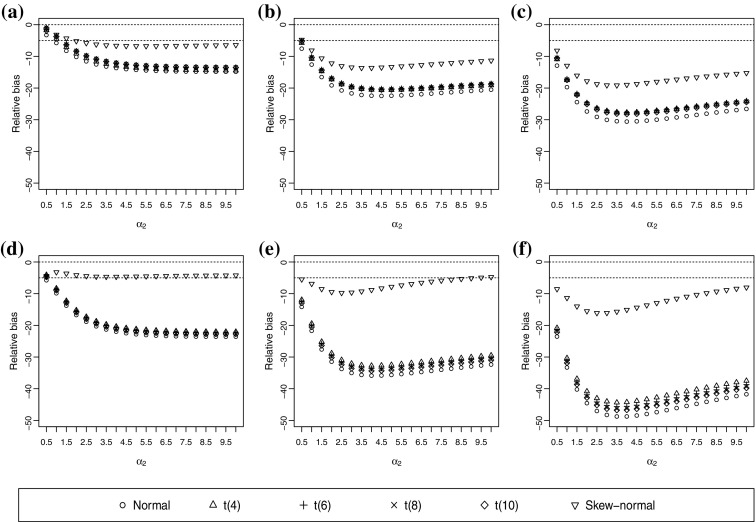
Relative bias (RB) of correlation estimates when the true underlying distribution is skew-t(4). The true correlation coefficient is 0.4. **a** $\alpha _1=0.1$ and both ordinal variables have five categories. **b** $\alpha _1=0.5$ and both ordinal variables have five categories. **c** $\alpha _1=1$ and both ordinal variables have five categories. **d** $\alpha _1=0.1$ and both ordinal variables have three categories. **e** $\alpha _1=0.5$ and both ordinal variables have three categories. **f** $\alpha _1=1$ and both ordinal variables have three categories.

When the true underlying distribution is a skew-t(4) distribution, the normal and t(*v*) underlying distributional assumptions lead to a largely biased polychoric correlation, except when both $\alpha _1$ and $\alpha _2$ are small (Figure 4). A small pair of $(\alpha _1,\alpha _2)$ only introduces a small skewness and kurtosis to the underlying distribution, which is similar to a t(4) distribution. As known from Experiment 1, the normal and t(*v*) underlying distributional assumptions are only slightly biased when the true underlying distribution is a *t* distribution. The skew-normal assumption may produce not so biased correlations when both ordinal variables have three categories and $\alpha _1$ is small (Figure 4). In general, the skew-normal assumption is less biased than the normal and *t*(*v*) assumptions. As the degrees of freedom of the skew-*t*(*v*) distribution increases, all distributional assumptions become less biased, and the skew-normal assumption in particular is often robust (See the figures in the supplementary materials). This effect is expected from the fact that the skew-normal distribution corresponds to the skew-*t* $(\infty )$ distribution. Nevertheless, the normal and t(*v*) assumptions still produce moderately or largely biased polychoric correlations. Similar to the conclusions from the underlying skew-normal distribution, a wrong distributional assumption tends to underestimate the polychoric correlation. Three categories in both ordinal variables generally lead to a higher RB in magnitudes than five categories in both ordinal variables; and a higher value of the true correlation coefficient generally leads to less biased estimates. Similar to the case in Experiment 2, the RMSEA can be misleading as well. A low RMSEA does not necessarily indicate a low RB (e.g. see Figure 26 in the supplementary materials).

#### Experiment 4

Table 1 shows that all the underlying distributional assumptions tend to be extremely biased when the true underlying distribution is a Pareto distribution. Similar to Experiments 2 and 3, the polychoric correlation tends to be underestimated across all conditions in Experiment 4. The skew-normal assumption produces a lower RB than the normal and t(*v*) assumptions, although all assumptions generally produce a large RB. The value of RMSEA tends to be small despite the heavily biased polychoric correlation. In particular, the RMSEA produced by the skew-normal assumption is always low. Note that the Pareto distribution is skewed. Thus, the skew-normal distribution assumption mimics the skewed pattern, although the true correlation coefficient is inconsistently estimated.

**Table 1:** Relative bias (RB) and root-mean-squared error of approximation (RMSEA) of polychoric correlations in Experiment 4.

$m_{U}$	$m_{V}$	$\rho _{0}$	Assumed distribution					
			Normal	*t*(4)	*t*(6)	*t*(8)	*t*(10)	Skew-normal
RB								
3	3	0.4	$-$47.18	$-$45.98	$-$46.29	$-$46.47	$-$46.59	$-$32.89
		0.6	$-$51.23	$-$50.17	$-$50.42	$-$50.57	$-$50.68	$-$38.78
3	5	0.4	$-$38.23	$-$37.83	$-$37.60	$-$37.60	$-$37.65	$-$32.12
		0.6	$-$43.50	$-$43.12	$-$42.92	$-$42.93	$-$42.97	$-$37.99
5	5	0.4	$-$36.26	$-$38.19	$-$36.94	$-$36.48	$-$36.28	$-$31.60
		0.6	$-$41.96	$-$43.35	$-$42.36	$-$42.01	$-$41.86	$-$37.41
RMSEA								
3	3	0.4	0.04	0.07	0.05	0.05	0.04	0.00
		0.6	0.05	0.08	0.06	0.06	0.06	0.01
3	5	0.4	0.04	0.05	0.04	0.04	0.04	0.01
		0.6	0.05	0.06	0.05	0.05	0.05	0.01
5	5	0.4	0.03	0.04	0.04	0.03	0.03	0.01
		0.6	0.04	0.05	0.04	0.04	0.04	0.01

### Asymptotic Variance

**Fig. 5:**
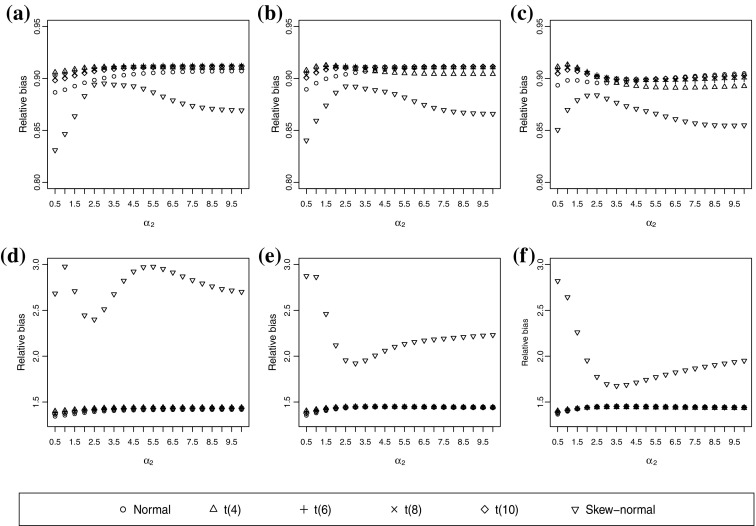
Asymptotic variances of correlation estimators when the true underlying distribution is skew-normal. The true correlation coefficient is 0.4. **a** $\alpha _1=0.1$ and both ordinal variables have five categories. **b** $\alpha _1=0.5$ and both ordinal variables have five categories. **c** $\alpha _1=1$ and both ordinal variables have five categories. **d** $\alpha _1=0.1$ and both ordinal variables have three categories. **e** $\alpha _1=0.5$ and both ordinal variables have three categories. **f** $\alpha _1=1$ and both ordinal variables have three categories.

In this subsection, the asymptotic variance is illustrated in Figure 5 when the true underlying distribution is a skew-normal distribution and both ordinal variables have five categories. The skew-normal assumption produces a lower asymptotic variance than do the other assumptions of distribution. The normal assumption often produces a similar asymptotic variance to the t assumption when $\rho _0=0.4$. Otherwise, the normal assumption tends to be slightly less variable than the t assumption. However, Figure 5 shows that the asymptotic variances under the skew-normal assumption can be substantially higher than the asymptotic variances of other assumptions of distribution when both ordinal variables have three categories. Recall that the normal assumption is asymptotically biased (Figure 2); however, a lower variance may lead to a lower mean squared error than the skew-normal assumption. Thus, although the skew-normal assumption is asymptotically unbiased, the correlation estimate is likely to have a larger departure from the true value than the normal assumption because of the large variation.

## Conclusion and Discussion

In this paper, we study robustness of polychoric correlation estimation against misspecification of underlying distributions. The asymptotic polychoric correlation and its asymptotic (co)variance are derived under the conditions of the support of assumed distributions. Unlike the continuous case, the correlation structure is not asymptotically unbiased any more. Although the bias is sometimes small, a large bias can occur, especially when the true underlying distribution is skewed but a bivariate normal or t distribution is assumed. It is seen from the numerical example that the skew-normal assumption performs as well as the conventional normal assumption when the true underlying distribution is a t distribution and improves the normal assumption when skewness exists.

Both Flora and Curran (2004) and Quiroga (1992) found that the normal assumption is robust against non-normal data generated from the Fleishman–Vale–Maurelli method. For example, the largest skewness and kurtosis considered in Flora and Curran (2004) are 1.25 and 3.75, respectively. The RB is lower than 10 in most conditions and is lower than 5 when the number of categories is five and $\rho _0=0.49$ (Flora & Curran, 2004, Table 2). Our results show that the polychoric correlation can be largely underestimated using the normal assumption when the true underlying distribution is a skew-normal distribution skewness and kurtosis of which are bounded by some small values. The bias becomes even higher when the true underlying distribution is skew-*t*(4) or a Pareto distribution in which cases the kurtosis is not well defined. Although the skew-normal assumption is also largely biased sometimes, it greatly improves the conventional normal assumption. Still, the skew-normal assumption has a much higher variance than the normal assumption when the number of categories is small. Thus, the volatility is high under the skew-normal assumption. Obviously, more studies are needed to investigate small sample volatility in order to provide suggestions for practice.

Lee and Lam (1988) suggested using the correct underlying distributional assumption to estimate more accurately the polychoric correlation if the ordinal data are asymmetric. Because the ordinal data indicate the loss of information when comparing with continuous data, we cannot have visual inspections of the underlying distribution. If the tests of the underlying distribution were rejected, the underlying distributional assumption is questionable, and an alternative distributional assumption should be used. In practice, several assumptions of underlying distribution can be tested and then the most plausible one chosen.

The normal distribution is a special case of the skew-normal distribution. We have shown that both distributions consistently estimate the polychoric correlation when the true distribution is normal. Thus, the skew-normal assumption, which is able to model skewness and kurtosis, is a natural extension to the conventional normal assumption and frequently outperforms the normal assumption. However, three parameters are simultaneously estimated in the skew-normal distribution. Because the thresholds are determined through $\alpha _1, \alpha _2$, and $\omega$, the gradient and Hessian matrix involve derivatives of the thresholds with respect to $\alpha _1, \alpha _2$, and $\omega$. Accordingly, it is computationally more difficult than the normal assumption. Besides, non-convergence and local optimizers are encountered in the present study and multiple starting values are used to obtain the correlation estimate.

Although only the t and skew-normal assumptions are illustrated as non-normal alternatives in the present study, other distributions that are differentiable with respect to unknown parameters can be used to estimate the correlation coefficient by the aid of Theorem 1 or Eq. (6). Its asymptotic variance and covariance can be estimated using Theorem 2 or Eq. (7). For example, the logistic distribution can be assumed in the two-step estimation and the skew-*t* distribution can be assumed in the variant of the two-step estimation. It will be of interest to derive analytical expressions for the skew-elliptical distribution family that consists of the skew-normal and skew-*t* distributions. Our numerical results demonstrate that the skew-normal assumption generally improves the conventional normal assumption in the imaginary case where *n* is infinite. It is worthy to conduct a simulation study to investigate the small sample bias in estimating the correlation coefficient and its effects on the bias of parameters in a SEM with ordinal data.

## Electronic supplementary material

Below is the link to the electronic supplementary material.

Supplementary material 1 (pdf 1853 KB)
