# Supplementary material for: Asymptotic Robustness Study of the Polychoric Correlation Estimation
Source: Psychometrika. 2016 Sep 22;82(1):67–85. doi: 10.1007/s11336-016-9512-2 (PMC5591612; doi:10.1007/s11336-016-9512-2)
Supplement: Supplementary file 1 — Supplementary material 1 (pdf 1853 KB) [file 11336_2016_9512_MOESM1_ESM.pdf]

SUPPLEMENTARY MATERIAL OF "ASYMPTOTIC ROBUSTNESS STUDY OF THE POLYCHORIC CORRELATION ESTIMATION"

May 17, 2016

SUPPLEMENTARY MATERIAL OF "ASYMPTOTIC ROBUSTNESS STUDY OF THE POLYCHORIC CORRELATION ESTIMATION"

Abstract

Asymptotic robustness against misspecification of the underlying distribution for the polychoric correlation estimation is studied. The asymptotic normality of the pseudo-maximum likelihood estimator is derived using the two-step estimation procedure. The t distribution assumption and the skew-normal distribution assumption are used as alternatives to the normal distribution assumption in a numerical study. The numerical results show that the underlying normal distribution can be substantially biased, even though skewness and kurtosis are not large. The skew-normal assumption generally produces a lower bias than the normal assumption. Thus, it is worth using a non-normal distributional assumption if the normal assumption is dubious.

Key words: underlying distribution, asymptotic covariance matrix, non-normality, pseudo-maximum likelihood.

1. General Distribution

Let $\boldsymbol{\theta}$ be a vector of free unknown parameters. The MLE of $\boldsymbol{\theta}$ maximizes

$$L(\boldsymbol{\theta}) = \sum_{i=1}^{m_U} \sum_{j=1}^{m_V} p_{ij} \log \ \pi_{ij,(\mathrm{H})}.$$

The gradient is

$$\frac{\partial}{\partial \boldsymbol{\theta}} L(\boldsymbol{\theta}) = \sum_{i=1}^{m_U} \sum_{j=1}^{m_V} \frac{p_{ij}}{\pi_{ij,(\mathrm{H})}} \frac{\partial \pi_{ij,(\mathrm{H})}}{\partial \boldsymbol{\theta}}, \tag{1}$$

and the hessian is

$$\frac{\partial^2}{\partial \boldsymbol{\theta} \partial \boldsymbol{\theta}^T} L(\boldsymbol{\theta}) = \sum_{i=1}^{m_U} \sum_{j=1}^{m_V} \left[\frac{p_{ij}}{\pi_{ij,(\mathrm{H})}} \frac{\partial^2 \pi_{ij,(\mathrm{H})}}{\partial \boldsymbol{\theta} \partial \boldsymbol{\theta}^T} - \frac{p_{ij}}{\pi_{ij,(\mathrm{H})}^2} \frac{\partial \pi_{ij,(\mathrm{H})}}{\partial \boldsymbol{\theta}} \frac{\partial \pi_{ij,(\mathrm{H})}}{\partial \boldsymbol{\theta}^T} \right]. \tag{2}$$

The gradient and hessian depend on $\frac{\partial \pi_{ij,(\mathrm{H})}}{\partial \boldsymbol{\theta}}$ and $\frac{\partial^2 \pi_{ij,(\mathrm{H})}}{\partial \boldsymbol{\theta} \partial \boldsymbol{\theta}^T}$ that depends on the distributional assumption. In the following two sections, necessary derivatives for a t distribution and a skew-normal distribution are provided.

2. T Distribution

The density function of a bivariate elliptical t distribution with v degrees of freedom is

$$f(x, y) = \frac{1}{2\pi\sqrt{1-\rho^2}} \left[1 + \frac{x^2 - 2\rho xy + y^2}{v(1-\rho^2)} \right]^{-(v+2)/2}$$

(Balakrishnan & Lai, 2009). The marginal distributions are student$-t$ distributions with degrees of freedom v. Note that

$$\begin{aligned}\frac{\partial}{\partial\rho}f(x,y) &= \frac{\rho}{1-\rho^2}f(x,y)+\frac{\rho x-y}{1-\rho^2}\frac{\partial}{\partial x}f(x,y)\\ &= \frac{\rho}{1-\rho^2}f(x,y)+\frac{\rho y-x}{1-\rho^2}\frac{\partial}{\partial y}f(x,y).\end{aligned}$$

Conditional on the thresholds τ and ξ, the gradient is

$$\frac{\partial}{\partial\rho}\int_{-\infty}^{\tau}\int_{-\infty}^{\xi}f(x,y)\,dydx=\int_{-\infty}^{\tau}\int_{-\infty}^{\xi}\frac{\partial}{\partial\rho}f(x,y)\,dydx=\int_{-\infty}^{\xi}f(\tau,y)\frac{\rho\tau-y}{1-\rho^2}dy.$$

If $\tau=\pm\infty$ or $\xi=\pm\infty$, the gradient is zero. The second order derivative is

$$\begin{aligned}\frac{\partial^2}{\partial\rho^2}\int_{-\infty}^{\tau}\int_{-\infty}^{\xi}f(x,y)\,dydx &= \int_{-\infty}^{\xi}\frac{\partial}{\partial\rho}\left[f(\tau,y)\frac{\rho\tau-y}{1-\rho^2}\right]dy\\ &=\frac{\rho}{(1-\rho^2)^2}\int_{-\infty}^{\xi}f(\tau,y)(\rho\tau-y)\,dy+f(\tau,\xi)\frac{(\xi-\rho\tau)(\tau-\rho\xi)}{(1-\rho^2)^2}.\end{aligned}$$

If $\tau=\pm\infty$ or $\xi=\pm\infty$, the second order derivative is zero. Both the first-order and second-order derivatives depend on

$$\int_{-\infty}^{\xi}f(\tau,y)(\rho\tau-y)\,dy,$$

which can be solved numerically. In order to obtain the asymptotic variance, the following derivatives are needed:

$$\frac{\partial}{\partial\tau}\int_{-\infty}^{\tau}\int_{-\infty}^{\xi} f\left(x,y\right)dydx = \int_{-\infty}^{\xi} f\left(\tau,y\right)dy,$$

$$\frac{\partial}{\partial\xi}\int_{-\infty}^{\tau}\int_{-\infty}^{\xi} f\left(x,y\right)dydx = \int_{-\infty}^{\tau} f\left(x,\xi\right)dx,$$

$$\frac{\partial^2}{\partial\rho\partial\tau}\int_{-\infty}^{\tau}\int_{-\infty}^{\xi} f\left(x,y\right)dydx = \frac{\rho\xi-\tau}{1-\rho^2} f\left(\tau,\xi\right),$$

$$\frac{\partial^2}{\partial\rho\partial\xi}\int_{-\infty}^{\tau}\int_{-\infty}^{\xi} f\left(x,y\right)dydx = \frac{\rho\tau-\xi}{1-\rho^2} f\left(\tau,\xi\right).$$

3. Skew-Normal Distribution

The density function of a bivariate skew-normal distribution is

$$f(x, y) = 2\phi_2(x, y; w)\,\Phi(\alpha_1 x + \alpha_2 y)$$

(Azzalini & Valle, 1996). If $\alpha_1 = \alpha_2 = 0$, it reduces to a bivariate normal distribution. The marginal distributions are

$$f(x) = 2\phi(x)\,\Phi(\alpha x),$$

where $\alpha = (\alpha_1 + w\alpha_2)/\sqrt{1 + (1 - w^2)\,\alpha_2^2}$ for X and $\alpha = (\alpha_2 + w\alpha_1)/\sqrt{1 + (1 - w^2)\,\alpha_1^2}$ for Y. For more details, readers are directed to Azzalini and Valle (1996) and Azzalini and Capitanio (2014). Since the thresholds depend on α_1, α_2, and w, the parameter vector is $\boldsymbol{\theta} = (\alpha_1, \alpha_2, w)^T$. To emphasize the random variable of marginal distribution, define $\alpha_x = (\alpha_1 + w\alpha_2)/\sqrt{1 + (1 - w^2)\,\alpha_2^2}$ and $\alpha_y = (\alpha_2 + w\alpha_1)/\sqrt{1 + (1 - w^2)\,\alpha_1^2}$.

3.1. Some Useful Results

In this section, results that will be called in later derivation are presented.

3.1.1. Some simple formulas

From Ulsson (1979) we know that

$$\frac{\partial}{\partial \rho} \int_{-\infty}^{\tau} \int_{-\infty}^{\xi} \phi_2 (x, y; \rho)\, dydx = \phi_2 (x, y; \rho),$$

$$\frac{\partial^2}{\partial \rho^2} \int_{-\infty}^{\tau} \int_{-\infty}^{\xi} \phi_2 (x, y; \rho)\, dydx = \frac{\partial}{\partial \rho} \phi_2 (x, y; \rho) = \phi_2 (x, y; \rho) \left[\frac{\rho}{1-\rho^2} + \frac{(\rho x - y)(\rho y - x)}{(1-\rho^2)^2} \right],$$

$$\frac{\partial}{\partial x} \phi_2 (x, y; \rho) = \phi_2 (x, y; \rho) \frac{\rho y - x}{1-\rho^2},$$

$$\frac{\partial}{\partial y} \phi_2 (x, y; \rho) = \phi_2 (x, y; \rho) \frac{\rho x - y}{1-\rho^2}.$$

Some formulas that are associated with the density function of a standard normal distribution are

$$\frac{\partial}{\partial x} \phi (x) = -x \phi (x),$$

$$\int_{-\infty}^{\tau} \phi \left(\frac{x-\mu}{\sigma} \right) dx = \sigma \Phi \left(\frac{x-\mu}{\sigma} \right),$$

$$\int_{-\infty}^{\tau} \exp \left\{ -\frac{(x-\mu)^2}{2\sigma^2} \right\} dx = \sqrt{2\pi} \sigma \Phi \left(\frac{\tau-\mu}{\sigma} \right).$$

3.1.2. Integral that will be solved numerically

There are some one-dimensional integrals that will be solved numerically, i.e.

$$\int_{-\infty}^{\tau} \phi\left(\frac{x}{\sqrt{c}}\right)\Phi\left(a-bx\right)dx, \tag{3}$$

$$\int_{-\infty}^{\tau} x^3\phi\left(x\right)\phi\left(\alpha x\right)dx, \tag{4}$$

$$\int_{-\infty}^{\tau} x^2\Phi\left(ax\right)dx, \tag{5}$$

$$\int_{-\infty}^{\xi} \phi_2\left(\tau,y;w\right)\Phi\left(\alpha_1\tau+\alpha_2 y\right)dy, \tag{6}$$

$$\int_{-\infty}^{\xi} y\phi_2\left(\tau,y;w\right)\Phi\left(\alpha_1\tau+\alpha_2 y\right)dy, \tag{7}$$

$$\int_{-\infty}^{\xi} y^2\phi_2\left(\tau,y;w\right)\Phi\left(\alpha_1\tau+\alpha_2 y\right)dy. \tag{8}$$

3.1.3. Other formulas

Based on the above results, the following formulas can be derived and will be used to derive the gradient and Hessian matrix. In order to avoid introducing too many unnecessary symbols, scalars a, b, and c may have different definitions from one formula to another.

$$\int_{-\infty}^{\tau} x\phi\left(\frac{x-\mu}{\sigma}\right)dx = -\sigma^2\phi\left(\frac{\tau-\mu}{\sigma}\right)+\mu\sigma\Phi\left(\frac{\tau-\mu}{\sigma}\right). \tag{9}$$

$$\int_{-\infty}^{\tau} x^2 \phi(x)\, dx = -\tau\phi(\tau) + \Phi(\tau). \tag{10}$$

$$\int_{-\infty}^{\tau} x^2 \phi\left(\frac{x-\mu}{\sigma}\right) dx = \mu^2\sigma\Phi\left(\frac{\tau-\mu}{\sigma}\right) + 2\mu\sigma^2 \int_{-\infty}^{\frac{\tau-\mu}{\sigma}} x\phi(x)\, dx + \sigma^3 \int_{-\infty}^{\frac{\tau-\mu}{\sigma}} x^2\phi(x)\, dx, \tag{11}$$

which can be solved by (9) and (10).

$$\int_{-\infty}^{\tau} \phi\left(\frac{x}{\sqrt{c}}\right)\phi(a-bx)\, dx = \begin{cases} 0 & \text{if } \tau = -\infty \text{ or } a = \infty, \\ \frac{1}{\sqrt{2\pi}}\sqrt{\frac{1}{b^2+\frac{1}{c}}}\exp\left\{-\frac{1}{2}\left[a^2 - \frac{a^2b^2}{b^2+\frac{1}{c}}\right]\right\}\Phi\left(\frac{\tau - \frac{ab}{b^2+1/c}}{\sqrt{1/(b^2+1/c)}}\right) & \text{otherwise,} \end{cases} \tag{12}$$

for any constants a, b, and $c \neq 0$.

$$\int_{-\infty}^{\tau} x\phi(x)\,\phi(ax)\, dx = -\frac{1}{\sqrt{2\pi}\,(1+a^2)}\phi\left(\sqrt{1+a^2}\tau\right), \tag{13}$$

for any constant a.

$$\int_{-\infty}^{\tau} 2\phi_2(x,\xi;w)\,\phi(\alpha_1 x + \alpha_2\xi)\, dx = \begin{cases} 0 & \text{if } \tau = -\infty \text{ or } \xi = \pm\infty \\ \frac{1}{\pi\sqrt{a}}\exp\left\{-\frac{\left(ac-b^2\right)\xi^2}{2a(1-w^2)}\right\}\Phi\left(\frac{\tau - b\xi/a}{\sqrt{(1-w^2)/a}}\right) & \text{otherwise,} \end{cases} \tag{14}$$

where $a = 1 + (1-w^2)\,\alpha_1^2$, $b = w - \alpha_1\alpha_2(1-w^2)$, and $c = 1 + (1-w^2)\,\alpha_2^2$.

$$\int_{-\infty}^{\xi} 2\phi_2(\tau, y; w)\,\phi(\alpha_1\tau + \alpha_2 y)\,dy = \begin{cases} 0 & \text{if } \xi = -\infty \text{ or } \tau = \pm\infty, \\ \frac{1}{\pi\sqrt{c}} \exp\left\{-\frac{\left(ac-b^2\right)\tau^2}{2c(1-w^2)}\right\} \Phi\left(\frac{\xi - b\tau/c}{\sqrt{(1-w^2)/c}}\right) & \text{otherwise,} \end{cases} \tag{15}$$

where $a = 1 + (1 - w^2)\,\alpha_1^2$, $b = w - \alpha_1\alpha_2\,(1 - w^2)$, and $c = 1 + (1 - w^2)\,\alpha_2^2$.

$$\int_{-\infty}^{\tau} 2x\phi_2(x, \xi; w)\,\phi(\alpha_1 x + \alpha_2\xi)\,dx = \frac{1}{\pi\sqrt{1-w^2}} \exp\left\{-\frac{(ac - b^2)\,\xi^2}{2a\,(1-w^2)}\right\} \int_{-\infty}^{\tau} x\phi\left(\frac{x - b\xi/a}{\sqrt{(1-w^2)/a}}\right) dx, \tag{16}$$

where $a = 1 + (1 - w^2)\,\alpha_1^2$, $b = w - \alpha_1\alpha_2\,(1 - w^2)$, and $c = 1 + (1 - w^2)\,\alpha_2^2$.

$$\int_{-\infty}^{\tau} x\phi\left(\frac{x}{\sqrt{c}}\right)\phi(a - bx)\,dx = \frac{1}{\sqrt{2\pi}} \exp\left\{-\frac{a^2}{2\,(b^2c+1)}\right\} \int_{-\infty}^{\tau} x\phi\left(\frac{x - \frac{abc}{b^2c+1}}{\sqrt{\frac{c}{b^2c+1}}}\right) dx, \tag{17}$$

for any constants a, b, and c. Here the integrals can be solved by (9).

$$\int_{-\infty}^{\tau} x\phi\left(\frac{x}{\sqrt{c}}\right)\Phi(a - bx)\,dx = \begin{cases} \int_{-\infty}^{\tau} x\phi\left(\frac{x}{\sqrt{c}}\right) dx & \text{if } a = \infty, \\ -c\phi\left(\frac{\tau}{\sqrt{c}}\right)\Phi(a - b\tau) - bc\int_{-\infty}^{\tau}\phi\left(\frac{x}{\sqrt{c}}\right)\phi(a - bx)\,dx & \text{otherwise,} \end{cases} \tag{18}$$

for any constants a, b, and c. Here $\int_{-\infty}^{\tau}\phi\left(\frac{x}{\sqrt{c}}\right)\phi\left(a-bx\right)dx$ is solved in (12).

$$\int\limits_{-\infty}^{\tau} x^2\phi\left(\frac{x}{\sqrt{c}}\right)\phi\left(a-bx\right)dx = \frac{1}{\sqrt{2\pi}}\exp\left\{-\frac{b^2}{2\left(a^2c+1\right)}\right\}\int\limits_{-\infty}^{\tau} x^2\phi\left(\frac{x-\frac{abc}{a^2c+1}}{\sqrt{\frac{c}{a^2c+1}}}\right)dx, \tag{19}$$

for any constants a, b, and c. Here the integrals can be solved by (11).

$$\int\limits_{-\infty}^{\tau} x^2\phi\left(\frac{x}{\sqrt{c}}\right)\Phi\left(a-bx\right)dx \tag{20}$$

$$= \begin{cases} 0 & \tau=-\infty, \\ c\int_{-\infty}^{\tau}\phi\left(\frac{x}{\sqrt{c}}\right)\Phi\left(a-bx\right)dx - bc\int_{-\infty}^{\tau}x\phi\left(\frac{x}{\sqrt{c}}\right)\phi\left(a-bx\right)dx & \tau=\infty, \\ -c\tau\phi\left(\frac{\tau}{\sqrt{c}}\right)\Phi\left(a-b\tau\right)+c\int_{-\infty}^{\tau}\phi\left(\frac{x}{\sqrt{c}}\right)\Phi\left(a-bx\right)dx - bc\int_{-\infty}^{\tau}x\phi\left(\frac{x}{\sqrt{c}}\right)\phi\left(a-bx\right)dx & \text{otherwise,} \end{cases} \tag{21}$$

for any constants a, b, and c. Here the integrals will be solved by (3) and (17).

$$\begin{aligned} &\int\limits_{-\infty}^{\tau} x^3\phi\left(\frac{x}{\sqrt{c}}\right)\Phi\left(a-bx\right)dx \\ &= -c\phi\left(\frac{\tau}{\sqrt{c}}\right)\tau^2\Phi\left(a-b\tau\right)+2c\int\limits_{-\infty}^{\tau}x\phi\left(\frac{x}{\sqrt{c}}\right)\Phi\left(a-bx\right)dx - bc\int\limits_{-\infty}^{\tau}x^2\phi\left(\frac{x}{\sqrt{c}}\right)\phi\left(a-bx\right)dx, \end{aligned} \tag{22}$$

for any constants a, b, and c. Here the integrals can be solved by (18) and (19).

$$\begin{aligned}
&\int_{-\infty}^{\xi} y^2\phi_2\left(\tau, y; w\right)\phi\left(\alpha_1\tau + \alpha_2 y\right)dy \\
=&\frac{1}{\sqrt{2\pi\left(1-w^2\right)}}\exp\left\{-\frac{\tau^2}{2}\right\}\int_{-\infty}^{\xi-w\tau} y^2\phi\left(\frac{y}{\sqrt{1-w^2}}\right)\phi\left(\alpha_1\tau+\alpha_2 w\tau+\alpha_2 y\right)dy \\
&+\frac{2w\tau}{\sqrt{2\pi\left(1-w^2\right)}}\exp\left\{-\frac{\tau^2}{2}\right\}\int_{-\infty}^{\xi-w\tau} y\phi\left(\frac{y}{\sqrt{1-w^2}}\right)\phi\left(\alpha_1\tau+\alpha_2 w\tau+\alpha_2 y\right)dy \\
&+\frac{w^2\tau^2}{\sqrt{2\pi\left(1-w^2\right)}}\exp\left\{-\frac{\tau^2}{2}\right\}\int_{-\infty}^{\xi-w\tau} \phi\left(\frac{y}{\sqrt{1-w^2}}\right)\phi\left(\alpha_1\tau+\alpha_2 w\tau+\alpha_2 y\right)dy,
\end{aligned} \tag{23}$$

which will be solved by (12), (17), and (19).

$$\frac{\partial\alpha_x}{\partial\boldsymbol{\theta}} = \begin{pmatrix} \frac{1}{\sqrt{1+(1-w^2)\alpha_2^2}} & \frac{w-\alpha_1\alpha_2+\alpha_1\alpha_2 w^2}{\left[1+(1-w^2)\alpha_2^2\right]^{1.5}} & \frac{\alpha_2+\alpha_2^3+\alpha_1\alpha_2^2 w}{\left[1+(1-w^2)\alpha_2^2\right]^{1.5}} \end{pmatrix}^T. \tag{24}$$

$$\frac{\partial\alpha_y}{\partial\boldsymbol{\theta}} = \begin{pmatrix} \frac{w-\alpha_1\alpha_2+\alpha_1\alpha_2 w^2}{\left[1+(1-w^2)\alpha_1^2\right]^{1.5}} & \frac{1}{\sqrt{1+(1-w^2)\alpha_1^2}} & \frac{\alpha_1+\alpha_1^3+\alpha_1^2\alpha_2 w}{\left[1+(1-w^2)\alpha_1^2\right]^{1.5}} \end{pmatrix}^T. \tag{25}$$

$$\frac{\partial\tau}{\partial\boldsymbol{\theta}} = -\frac{1}{\phi\left(\tau\right)\Phi\left(\alpha_x\tau\right)}\int_{-\infty}^{\tau} x\phi\left(x\right)\phi\left(\alpha_x x\right)dx\frac{\partial\alpha_x}{\partial\boldsymbol{\theta}}, \tag{26}$$

which can be solved by (13) and (24).

$$\frac{\partial\xi}{\partial\boldsymbol{\theta}} = -\frac{1}{\phi\left(\xi\right)\Phi\left(\alpha_y\xi\right)}\int_{-\infty}^{\xi} y\phi\left(y\right)\phi\left(\alpha_y y\right)dy\frac{\partial\alpha_y}{\partial\boldsymbol{\theta}}, \tag{27}$$

which can be solved by (12) and (25).

$$\frac{\partial^2\alpha_x}{\partial\boldsymbol{\theta}\partial\boldsymbol{\theta}^T}=\begin{pmatrix} 0 & -\frac{\left(1-w^2\right)\alpha_2}{c^{1.5}} & \frac{w\alpha_2^2}{c^{1.5}} \\ -\frac{\alpha_2\left(1-w^2\right)}{c^{1.5}} & \frac{-\alpha_1\left(1-w^2\right)c-3\alpha_2\left(1-w^2\right)b}{c^{2.5}} & \frac{(1+2\alpha_1\alpha_2 w)c+3w\alpha_2^2 b}{c^{2.5}} \\ \frac{w\alpha_2^2}{c^{1.5}} & \frac{(1+2\alpha_1\alpha_2 w)c+3w\alpha_2^2 b}{c^{2.5}} & \frac{\alpha_1\alpha_2^2 c+3w\alpha_2^3\left(1+\alpha_2^2+\alpha_1\alpha_2 w\right)}{c^{2.5}} \end{pmatrix}, \tag{28}$$

where $b=w-\alpha_1\alpha_2\left(1-w^2\right)$ and $c=1+\left(1-w^2\right)\alpha_2^2$.

$$\frac{\partial^2\alpha_y}{\partial\boldsymbol{\theta}\partial\boldsymbol{\theta}^T}=\begin{pmatrix} \frac{-\alpha_2\left(1-w^2\right)a-3\alpha_1\left(1-w^2\right)b}{a^{2.5}} & \frac{-\left(1-w^2\right)\alpha_1}{a^{1.5}} & \frac{(1+2\alpha_1\alpha_2 w)a+3w\alpha_1^2 b}{a^{2.5}} \\ \frac{-\left(1-w^2\right)\alpha_1}{a^{1.5}} & 0 & \frac{w\alpha_1^2}{a^{1.5}} \\ \frac{(1+2\alpha_1\alpha_2 w)a+3w\alpha_1^2 b}{a^{2.5}} & \frac{w\alpha_1^2}{a^{1.5}} & \frac{\alpha_1^2\alpha_2 a+3w\alpha_1^3\left(1+\alpha_1^2+\alpha_1\alpha_2 w\right)}{a^{2.5}} \end{pmatrix}, \tag{29}$$

where $a=1+\left(1-w^2\right)\alpha_1^2$, and $b=w-\alpha_1\alpha_2\left(1-w^2\right)$.

$$\begin{aligned} &\frac{\partial}{\partial\boldsymbol{\theta}}\int_{-\infty}^{\tau}\phi\left(\frac{x}{\sqrt{c}}\right)\phi\left(a-bx\right)dx \\ =&\frac{1}{2c^2}\frac{\partial c}{\partial\boldsymbol{\theta}}\int_{-\infty}^{\tau}x^2\phi\left(\frac{x}{\sqrt{c}}\right)\phi\left(a-bx\right)dx-a\frac{\partial a}{\partial\boldsymbol{\theta}}\int_{-\infty}^{\tau}\phi\left(\frac{x}{\sqrt{c}}\right)\phi\left(a-bx\right)dx \\ &+a\frac{\partial b}{\partial\boldsymbol{\theta}}\int_{-\infty}^{\tau}x\phi\left(\frac{x}{\sqrt{c}}\right)\phi\left(a-bx\right)dx+b\frac{\partial a}{\partial\boldsymbol{\theta}}\int_{-\infty}^{\tau}x\phi\left(\frac{x}{\sqrt{c}}\right)\phi\left(a-bx\right)dx \\ &-b\frac{\partial b}{\partial\boldsymbol{\theta}}\int_{-\infty}^{\tau}x^2\phi\left(\frac{x}{\sqrt{c}}\right)\phi\left(a-bx\right)dx+\phi\left(\frac{\tau}{\sqrt{c}}\right)\phi\left(a-b\tau\right)\frac{\partial\tau}{\partial\boldsymbol{\theta}}, \end{aligned} \tag{30}$$

for functions a, b, and c that depend on θ. It can be solved by (12), (17), (19), and (26). The final expression depends on the expression of a, b, and c.

$$
\begin{aligned}
&\frac{\partial}{\partial \boldsymbol{\theta}} \int_{-\infty}^{\tau} \phi\left(\frac{x}{\sqrt{c}}\right) \Phi\left(a-bx\right) dx \\
=&\frac{1}{2c^2}\frac{\partial c}{\partial \boldsymbol{\theta}} \int_{-\infty}^{\tau} x^2 \phi\left(\frac{x}{\sqrt{c}}\right) \Phi\left(a-bx\right) dx + \frac{\partial a}{\partial \boldsymbol{\theta}} \int_{-\infty}^{\tau} \phi\left(\frac{x}{\sqrt{c}}\right) \phi\left(a-bx\right) dx \\
&- \frac{\partial b}{\partial \boldsymbol{\theta}} \int_{-\infty}^{\tau} x \phi\left(\frac{x}{\sqrt{c}}\right) \phi\left(a-bx\right) dx + \phi\left(\frac{\tau}{\sqrt{c}}\right) \Phi\left(a-b\tau\right) \frac{\partial \tau}{\partial \theta},
\end{aligned} \tag{31}
$$

for functions a, b, and c that depend on θ. It can be solved by (12), (17), (20), and 26. The final expression depends on the expression of a, b, and c.

If $\tau = -\infty$

$$
\frac{\partial}{\partial \boldsymbol{\theta}} \int_{-\infty}^{\tau} x \phi\left(\frac{x}{\sqrt{c}}\right) \Phi\left(a-bx\right) dx = 0.
$$

If $\tau \neq -\infty$,

$$
\begin{aligned}
&\frac{\partial}{\partial \boldsymbol{\theta}} \int_{-\infty}^{\tau} x \phi\left(\frac{x}{\sqrt{c}}\right) \Phi\left(a-bx\right) dx \\
=&\frac{1}{2c^2}\frac{\partial c}{\partial \boldsymbol{\theta}} \int_{-\infty}^{\tau} x^3 \phi\left(\frac{x}{\sqrt{c}}\right) \Phi\left(a-bx\right) dx + \frac{\partial a}{\partial \boldsymbol{\theta}} \int_{-\infty}^{\tau} x \phi\left(\frac{x}{\sqrt{c}}\right) \phi\left(a-bx\right) dx \\
&- \frac{\partial b}{\partial \boldsymbol{\theta}} \int_{-\infty}^{\tau} x^2 \phi\left(\frac{x}{\sqrt{c}}\right) \phi\left(a-bx\right) dx + \tau \phi\left(\frac{\tau}{\sqrt{c}}\right) \Phi\left(a-b\tau\right) \frac{\partial \tau}{\partial \boldsymbol{\theta}}.
\end{aligned} \tag{32}
$$

The integrals can be solved by (17), (19), and (22). If $\tau = \infty$, the last term in (31) is zero.

$$\frac{\partial}{\partial \boldsymbol{\theta}} \int_{-\infty}^{\tau} x\phi(x)\,\phi(\alpha x)\,dx = -\alpha \frac{\partial \alpha}{\partial \boldsymbol{\theta}} \int_{-\infty}^{\tau} x^3 \phi(x)\,\phi(\alpha x)\,dx + \tau\phi(\tau)\,\phi(\alpha\tau)\,\frac{\partial \tau}{\partial \boldsymbol{\theta}}, \tag{33}$$

where α is a function of $\boldsymbol{\theta}$.

$$\frac{\partial}{\partial \boldsymbol{\theta}} \int_{-\infty}^{\xi} y\phi(y)\,\phi(\alpha y)\,dy = -\alpha \frac{\partial \alpha}{\partial \boldsymbol{\theta}} \int_{-\infty}^{\xi} y^3 \phi(y)\,\phi(\alpha y)\,dy + \xi\phi(\xi)\,\phi(\alpha\xi)\,\frac{\partial \xi}{\partial \boldsymbol{\theta}}, \tag{34}$$

where α is a function of $\boldsymbol{\theta}$.

$$\begin{aligned}\frac{\partial^2 \tau}{\partial \boldsymbol{\theta} \partial \boldsymbol{\theta}^T} =& \frac{-\tau\phi(\tau)\,\frac{\partial \tau}{\partial \boldsymbol{\theta}}\Phi(\alpha_x\tau) + \phi(\tau)\,\phi(\alpha_x\tau)\left(\frac{\partial \alpha_x}{\partial \boldsymbol{\theta}}\tau + \alpha_x \frac{\partial \tau}{\partial \boldsymbol{\theta}}\right)}{\left[\phi(\tau)\,\Phi(\alpha_x\tau)\right]^2} \int_{-\infty}^{\tau} x\phi(x)\,\phi(\alpha_x x)\,dx \frac{\partial \alpha_x}{\partial \boldsymbol{\theta}^T} \\ & - \frac{1}{\phi(\tau)\,\Phi(\alpha_x\tau)} \left(\frac{\partial}{\partial \boldsymbol{\theta}} \int_{-\infty}^{\tau} x\phi(x)\,\phi(\alpha_x x)\,dx\right) \frac{\partial \alpha_x}{\partial \boldsymbol{\theta}^T} \\ & - \frac{1}{\phi(\tau)\,\Phi(\alpha_x\tau)} \int_{-\infty}^{\tau} x\phi(x)\,\phi(\alpha_x x)\,dx \frac{\partial^2 \alpha_x}{\partial \boldsymbol{\theta} \partial \boldsymbol{\theta}^T},\end{aligned} \tag{35}$$

which can be solved by (24), (26), (28), and (33).

$$\begin{aligned}\frac{\partial^2 \xi}{\partial \boldsymbol{\theta} \partial \boldsymbol{\theta}^T} =& \frac{-\xi\phi(\xi)\,\frac{\partial \xi}{\partial \boldsymbol{\theta}}\Phi(\alpha_y\xi) + \phi(\xi)\,\phi(\alpha_y\xi)\left(\frac{\partial \alpha_y}{\partial \boldsymbol{\theta}}\xi + \alpha_y \frac{\partial \xi}{\partial \boldsymbol{\theta}}\right)}{\left[\phi(\xi)\,\Phi(\alpha_y\xi)\right]^2} \int_{-\infty}^{\xi} y\phi(y)\,\phi(\alpha_y y)\,dy \frac{\partial \alpha_y}{\partial \boldsymbol{\theta}^T} \\ & - \frac{1}{\phi(\xi)\,\Phi(\alpha_y\xi)} \left(\frac{\partial}{\partial \boldsymbol{\theta}} \int_{-\infty}^{\xi} y\phi(y)\,\phi(\alpha_y y)\,dy\right) \frac{\partial \alpha_y}{\partial \boldsymbol{\theta}^T} \\ & - \frac{1}{\phi(\xi)\,\Phi(\alpha_y\xi)} \int_{-\infty}^{\xi} y\phi(y)\,\phi(\alpha_y y)\,dy \frac{\partial^2 \alpha_y}{\partial \boldsymbol{\theta} \partial \boldsymbol{\theta}^T},\end{aligned} \tag{36}$$

which can be solved by (25), (27), (29), and (30).

$$\begin{aligned}\frac{\partial}{\partial \boldsymbol{\theta}}\phi_2\left(\tau, y; w\right) =& \frac{1}{4\pi\left(1-w^2\right)^{1.5}}\frac{\partial w^2}{\partial \boldsymbol{\theta}}\exp\left\{-\frac{\tau^2-2w\tau y+y^2}{2\left(1-w^2\right)}\right\}\\ &-\phi_2\left(\tau, y; w\right)\frac{\left[\tau\frac{\partial \tau}{\partial \boldsymbol{\theta}}-y\left(\frac{\partial w}{\partial \boldsymbol{\theta}}\tau+w\frac{\partial \tau}{\partial \boldsymbol{\theta}}\right)\right]2\left(1-w^2\right)+\left(\tau^2-2w\tau y+y^2\right)\frac{\partial w^2}{\partial \boldsymbol{\theta}}}{2\left(1-w^2\right)^2},\end{aligned} \tag{37}$$

which can be solved by (26).

$$\begin{aligned}\frac{\partial}{\partial \boldsymbol{\theta}}\phi_2\left(x, \xi; w\right) =& \frac{1}{4\pi\left(1-w^2\right)^{1.5}}\frac{\partial w^2}{\partial \boldsymbol{\theta}}\exp\left\{-\frac{x^2-2wx\xi+\xi^2}{2\left(1-w^2\right)}\right\}\\ &-\phi_2\left(x, \xi; w\right)\frac{\left(-\frac{\partial w}{\partial \boldsymbol{\theta}}x\xi-wx\frac{\partial \xi}{\partial \boldsymbol{\theta}}+\frac{\partial \xi}{\partial \boldsymbol{\theta}}\xi\right)2\left(1-w^2\right)+\left(x^2-2wx\xi+\xi^2\right)\frac{\partial w^2}{\partial \boldsymbol{\theta}}}{2\left(1-w^2\right)^2},\end{aligned} \tag{38}$$

which can be solved by (27).

$$\begin{aligned}\frac{\partial}{\partial \boldsymbol{\theta}}\phi_2\left(\tau, \xi; w\right) =& \frac{1}{2\left(1-w^2\right)}\frac{\partial w^2}{\partial \boldsymbol{\theta}}\phi_2\left(\tau, \xi; w\right)\\ &-\phi_2\left(\tau, \xi; w\right)\frac{\left(\tau\frac{\partial \tau}{\partial \boldsymbol{\theta}}-\frac{\partial w}{\partial \boldsymbol{\theta}}\tau\xi-w\frac{\partial \tau}{\partial \boldsymbol{\theta}}\xi-w\tau\frac{\partial \xi}{\partial \boldsymbol{\theta}}+\xi\frac{\partial \xi}{\partial \boldsymbol{\theta}}\right)2\left(1-w^2\right)+\left(\tau^2-2w\tau\xi+\xi^2\right)\frac{\partial w^2}{\partial \boldsymbol{\theta}}}{2\left(1-w^2\right)^2},\end{aligned} \tag{39}$$

which can be solved by (26) and (27).

$$
\begin{aligned}
&\frac{\partial}{\partial \boldsymbol{\theta}}\left[\int_{-\infty}^{\tau} 2\phi_2\left(x,\xi;w\right)\Phi\left(\alpha_1 x+\alpha_2\xi\right)dx\right]\\
=&\frac{1}{1-w^2}\frac{\partial w^2}{\partial \boldsymbol{\theta}}\phi\left(\xi\right)\int_{-\infty}^{\tau-w\xi}\phi\left(\frac{x}{\sqrt{1-w^2}}\right)\Phi\left(\alpha_1 w\xi+\alpha_2\xi+\alpha_1 x\right)dx\\
&+\frac{\frac{\partial w}{\partial \boldsymbol{\theta}}\xi+w\frac{\partial \xi}{\partial \boldsymbol{\theta}}}{1-w^2}\int_{-\infty}^{\tau} 2x\phi_2\left(x,\xi;w\right)\Phi\left(\alpha_1 x+\alpha_2\xi\right)dx-\frac{1}{1-w^2}\frac{\partial \xi}{\partial \boldsymbol{\theta}}\xi\int_{-\infty}^{\tau} 2\phi_2\left(x,\xi;w\right)\Phi\left(\alpha_1 x+\alpha_2\xi\right)dx\\
&-\frac{\frac{\partial w^2}{\partial \boldsymbol{\theta}}}{\left(1-w^2\right)^2}\int_{-\infty}^{\tau} x^2\phi_2\left(x,\xi;w\right)\Phi\left(\alpha_1 x+\alpha_2\xi\right)dx+\frac{2w\xi\frac{\partial w^2}{\partial \boldsymbol{\theta}}}{\left(1-w^2\right)^2}\int_{-\infty}^{\tau} x\phi_2\left(x,\xi;w\right)\Phi\left(\alpha_1 x+\alpha_2\xi\right)dx\\
&-\frac{\xi^2}{\left(1-w^2\right)^2}\frac{\partial w^2}{\partial \boldsymbol{\theta}}\int_{-\infty}^{\tau} \phi_2\left(x,\xi;w\right)\Phi\left(\alpha_1 x+\alpha_2\xi\right)dx+\frac{\partial \alpha_1}{\partial \boldsymbol{\theta}}\int_{-\infty}^{\tau} 2x\phi_2\left(x,\xi;w\right)\phi\left(\alpha_1 x+\alpha_2\xi\right)dx\\
&+\left[\frac{\partial \alpha_2}{\partial \boldsymbol{\theta}}\xi+\alpha_2\frac{\partial \xi}{\partial \boldsymbol{\theta}}\right]\int_{-\infty}^{\tau} 2\phi_2\left(x,\xi;w\right)\phi\left(\alpha_1 x+\alpha_2\xi\right)dx+2\phi_2\left(\tau,\xi;w\right)\Phi\left(\alpha_1\tau+\alpha_2\xi\right)\frac{\partial \tau}{\partial \boldsymbol{\theta}},
\end{aligned}
\tag{40}
$$

which can be solved by (3), (6), (7), (8), (14), (16) (26) and (27).

$$
\begin{aligned}
&\frac{\partial}{\partial \boldsymbol{\theta}}\left[\int_{-\infty}^{\xi} 2\phi_2\left(\tau, y; w\right)\Phi\left(\alpha_1\tau+\alpha_2 y\right)dy\right] \\
=&\frac{1}{\sqrt{2\pi}\left(1-w^2\right)^{1.5}}\frac{\partial w^2}{\partial \boldsymbol{\theta}}\exp\left\{-\frac{\tau^2}{2}\right\}\int_{-\infty}^{\xi-w\tau}\phi\left(\frac{y}{\sqrt{1-w^2}}\right)\Phi\left(\alpha_1\tau+\alpha_2 w\tau+\alpha_2 y\right)dy \\
&+2\frac{\frac{\partial w}{\partial \boldsymbol{\theta}}\tau+w\frac{\partial \tau}{\partial \boldsymbol{\theta}}}{1-w^2}\int_{-\infty}^{\xi} y\phi_2\left(\tau, y; w\right)\Phi\left(\alpha_1\tau+\alpha_2 y\right)dy-2\frac{\tau\frac{\partial \tau}{\partial \boldsymbol{\theta}}}{1-w^2}\int_{-\infty}^{\xi}\phi_2\left(\tau, y; w\right)\Phi\left(\alpha_1\tau+\alpha_2 y\right)dy \\
&-\frac{\frac{\partial w^2}{\partial \boldsymbol{\theta}}}{\left(1-w^2\right)^2}\int_{-\infty}^{\xi} y^2\phi_2\left(\tau, y; w\right)\Phi\left(\alpha_1\tau+\alpha_2 y\right)dy+\frac{2w\tau\frac{\partial w^2}{\partial \boldsymbol{\theta}}}{\left(1-w^2\right)^2}\int_{-\infty}^{\xi} y\phi_2\left(\tau, y; w\right)\Phi\left(\alpha_1\tau+\alpha_2 y\right)dy \\
&-\frac{\tau^2\frac{\partial w^2}{\partial \boldsymbol{\theta}}}{\left(1-w^2\right)^2}\int_{-\infty}^{\xi}\phi_2\left(\tau, y; w\right)\Phi\left(\alpha_1\tau+\alpha_2 y\right)dy+\frac{\partial \alpha_2}{\partial \boldsymbol{\theta}}\int_{-\infty}^{\xi} 2y\phi_2\left(\tau, y; w\right)\phi\left(\alpha_1\tau+\alpha_2 y\right)dy \\
&+\left(\frac{\partial \alpha_1}{\partial \boldsymbol{\theta}}\tau+\alpha_1\frac{\partial \tau}{\partial \boldsymbol{\theta}}\right)\int_{-\infty}^{\xi} 2\phi_2\left(\tau, y; w\right)\phi\left(\alpha_1\tau+\alpha_2 y\right)dy+2\phi_2\left(\tau, \xi; w\right)\Phi\left(\alpha_1\tau+\alpha_2\xi\right)\frac{\partial \xi}{\partial \boldsymbol{\theta}},
\end{aligned}
\tag{41}
$$

which can be solved by (3), (6), (7), (8), (15), (16) (26) and (27).

3.2. Gradient

The gradient can be derived as functions of the results presented above. In the rest part of the paper, define $a = 1 + \left(1 - w^2\right)\alpha_1^2$, $b = w - \alpha_1\alpha_2\left(1 - w^2\right)$, and $c = 1 + \left(1 - w^2\right)\alpha_2^2$.

3.2.1. Gradient with respect to α_1

Consider the upper limits τ and ξ that are functions of $\boldsymbol{\theta}$.

$$\begin{aligned}\frac{\partial}{\partial \alpha_1}\int_{-\infty}^{\tau}\int_{-\infty}^{\xi} f(x,y)\,dydx &= \int_{-\infty}^{\tau}\int_{-\infty}^{\xi} 2\phi_2(x,y;w)\frac{\partial}{\partial \alpha_1}\left[\Phi(\alpha_1 x+\alpha_2 y)\right]dydx \\ &+ \frac{\partial \tau}{\partial \alpha_1}\int_{-\infty}^{\xi} 2\phi_2(\tau,y;w)\,\Phi(\alpha_1\tau+\alpha_2 y)\,dy \\ &+ \frac{\partial \xi}{\partial \alpha_1}\int_{-\infty}^{\tau} 2\phi_2(x,\xi;w)\,\Phi(\alpha_1 x+\alpha_2\xi)\,dx. \end{aligned} \tag{42}$$

The first term in (42) can be expressed as

$$\begin{aligned}&\int_{-\infty}^{\tau}\int_{-\infty}^{\xi} 2\phi_2(x,y;w)\frac{\partial}{\partial \alpha_1}\left[\Phi(\alpha_1 x+\alpha_2 y)\right]dydx \\ &= -\frac{2\sqrt{1-w^2}}{\sqrt{2\pi}a}\int_{-\infty}^{\xi}\phi\left(\frac{y}{\sqrt{\frac{a(1-w^2)}{ac-b^2}}}\right)\phi\left(\frac{a\tau}{\sqrt{a(1-w^2)}}-\frac{by}{\sqrt{a(1-w^2)}}\right)dy \\ &\quad+\frac{2}{\sqrt{2\pi}}\frac{b}{a}\sqrt{\frac{1}{a}}\int_{-\infty}^{\xi} y\phi\left(\frac{y}{\sqrt{\frac{a(1-w^2)}{ac-b^2}}}\right)\Phi\left(\frac{a\tau}{\sqrt{a(1-w^2)}}-\frac{by}{\sqrt{a(1-w^2)}}\right)dy,\end{aligned}$$

which can be solved by (12) and (18). If $\tau=\infty$, the first part is zero and $\Phi\left(\frac{a\tau-by}{\sqrt{a(1-w^2)}}\right)=1$ since a is always positive. The second term in (42) can be solved by (6) and (26). The third term in (42) can be solved by (6) and (27).

3.2.2. Gradient with respect to α_2

The gradient with respect to α_2 is

$$\begin{aligned}\frac{\partial}{\partial \alpha_2}\int_{-\infty}^{\tau}\int_{-\infty}^{\xi} f(x,y)\,dydx &= \int_{-\infty}^{\tau}\int_{-\infty}^{\xi} 2\phi_2(x,y;w)\frac{\partial}{\partial \alpha_2}\left[\Phi(\alpha_1 x+\alpha_2 y)\right]dydx \\ &+ \frac{\partial \tau}{\partial \alpha_2}\int_{-\infty}^{\xi} 2\phi_2(\tau,y;w)\,\Phi(\alpha_1\tau+\alpha_2 y)\,dy \\ &+ \frac{\partial \xi}{\partial \alpha_2}\int_{-\infty}^{\tau} 2\phi_2(x,\xi;w)\,\Phi(\alpha_1 x+\alpha_2\xi)\,dx. \qquad (43)\end{aligned}$$

The first term in (43) can be expressed as

$$\begin{aligned}&\int_{-\infty}^{\tau}\int_{-\infty}^{\xi} 2\phi_2(x,y;w)\frac{\partial}{\partial \alpha_2}\left[\Phi(\alpha_1 x+\alpha_2 y)\right]dydx \\ &= -\frac{2\sqrt{1-w^2}}{\sqrt{2\pi}c}\int_{-\infty}^{\tau}\phi\left(\frac{x}{\sqrt{\frac{c(1-w^2)}{ac-b^2}}}\right)\phi\left(\frac{c\xi}{\sqrt{c(1-w^2)}}-\frac{bx}{\sqrt{c(1-w^2)}}\right)dx \\ &\quad+\frac{2}{\sqrt{2\pi}}\frac{b}{c}\sqrt{\frac{1}{c}}\int_{-\infty}^{\tau}x\phi\left(\frac{x}{\sqrt{\frac{c(1-w^2)}{ac-b^2}}}\right)\Phi\left(\frac{c\xi}{\sqrt{c(1-w^2)}}-\frac{bx}{\sqrt{c(1-w^2)}}\right)dx,\end{aligned}$$

which can be solved by (12) and (18). If $\xi=\infty$, the first part is zero and $\Phi\left(\frac{c\xi-bx}{\sqrt{c(1-w^2)}}\right)=1$. The second term in (43) can be solved by (6) and (26). The third term in (43) can be solved by (6) and (27).

3.2.3. Gradient with respect to w

The gradient with respect to w is

$$\frac{\partial}{\partial w}\int_{-\infty}^{\tau}\int_{-\infty}^{\xi} f(x,y)\,dydx = \int_{-\infty}^{\tau}\int_{-\infty}^{\xi} 2\frac{\partial}{\partial w}\left[\phi_2(x,y;w)\,\Phi(\alpha_1 x+\alpha_2 y)\right]dydx \tag{44}$$

$$+\frac{\partial \tau}{\partial w}\int_{-\infty}^{\xi} 2\phi_2(\tau,y;w)\,\Phi(\alpha_1\tau+\alpha_2 y)\,dy \tag{45}$$

$$+\frac{\partial \xi}{\partial w}\int_{-\infty}^{\tau} 2\phi_2(x,\xi;w)\,\Phi(\alpha_1 x+\alpha_2\xi)\,dx. \tag{46}$$

Consider Term (44) first.

$$\begin{aligned}
(44) =& \int_{-\infty}^{\tau}\int_{-\infty}^{\xi} 2\phi_2(x,y;w)\left[\frac{w}{1-w^2}+\frac{(wx-y)(wy-x)}{(1-w^2)^2}\right]\Phi(\alpha_1 x+\alpha_2 y)\,dydx\\
=&2\Phi(\alpha_1\tau+\alpha_2\xi)\,\phi_2(\tau,\xi;w)-\int_{-\infty}^{\xi} 2\alpha_2\phi_2(\tau,y;w)\,\phi(\alpha_1\tau+\alpha_2 y)\,dy\\
&-\int_{-\infty}^{\tau} 2\alpha_1\phi_2(x,\xi;w)\,\phi(\alpha_1 x+\alpha_2\xi)\,dx\\
&-\int_{-\infty}^{\tau}\int_{-\infty}^{\xi} 2\alpha_1\alpha_2(\alpha_1 x+\alpha_2 y)\,\phi_2(x,y;w)\,\phi(\alpha_1 x+\alpha_2 y)\,dydx\\
=&2\Phi(\alpha_1\tau+\alpha_2\xi)\,\phi_2(\tau,\xi;w)-\alpha_1(14)-\alpha_2(15)\\
&-\int_{-\infty}^{\tau}\int_{-\infty}^{\xi} 2\alpha_1\alpha_2(\alpha_1 x+\alpha_2 y)\,\phi_2(x,y;w)\,\phi(\alpha_1 x+\alpha_2 y)\,dydx.
\end{aligned}$$

Note that

$$\int_{-\infty}^{\tau} (\alpha_1 x + \alpha_2 y)\, \phi_2 (x, y; w)\, \phi (\alpha_1 x + \alpha_2 y)\, dx$$
$$= \frac{1}{(2\pi)^{3/2} \sqrt{1-w^2}} \exp\left\{-\frac{ac-b^2}{2a(1-w^2)} y^2\right\} \int_{-\infty}^{\tau} (\alpha_1 x + \alpha_2 y) \exp\left\{-\frac{\left(x-\frac{b}{a}y\right)^2}{2\frac{1-w^2}{a}}\right\} dx.$$

The integral in the above equation can be expressed as

$$\int_{-\infty}^{\tau} (\alpha_1 x + \alpha_2 y) \exp\left\{-\frac{\left(x-\frac{b}{a}y\right)^2}{2\frac{1-w^2}{a}}\right\} dx$$
$$= \alpha_1 \sqrt{2\pi} \left[-\frac{1-w^2}{a} \phi\left(\frac{\tau-\frac{b}{a}y}{\sqrt{\frac{1-w^2}{a}}}\right) + \frac{b}{a} y \sqrt{\frac{1-w^2}{a}} \Phi\left(\frac{\tau-\frac{b}{a}y}{\sqrt{\frac{1-w^2}{a}}}\right)\right] + \alpha_2 y \sqrt{2\pi} \sqrt{\frac{1-w^2}{a}} \Phi\left(\frac{x-\frac{b}{a}y}{\sqrt{\frac{1-w^2}{a}}}\right).$$

Therefore

$$\int_{-\infty}^{\tau} (\alpha_1 x + \alpha_2 y)\, \phi_2 (x, y; w)\, \phi (\alpha_1 x + \alpha_2 y)\, dx$$
$$= \frac{\alpha_1}{2\pi\sqrt{1-w^2}} \exp\left\{-\frac{ac-b^2}{2a(1-w^2)} y^2\right\} \left[-\frac{1-w^2}{a} \phi\left(\frac{\tau-\frac{b}{a}y}{\sqrt{\frac{1-w^2}{a}}}\right) + \frac{b}{a} y \sqrt{\frac{1-w^2}{a}} \Phi\left(\frac{\tau-\frac{b}{a}y}{\sqrt{\frac{1-w^2}{a}}}\right)\right]$$
$$+ \frac{a_2 y}{2\pi\sqrt{a}} \exp\left\{-\frac{ac-b^2}{2a(1-w^2)} y^2\right\} \Phi\left(\frac{\tau-\frac{b}{a}y}{\sqrt{\frac{1-w^2}{a}}}\right).$$

Consequently,

$$\int_{-\infty}^{\xi}\int_{-\infty}^{\tau} (\alpha_1 x + \alpha_2 y)\,\phi_2(x, y; w)\,\phi(\alpha_1 x + \alpha_2 y)\,dxdy$$

$$= -\int_{-\infty}^{\xi} \frac{\alpha_1}{2\pi\sqrt{1-w^2}} \exp\left\{-\frac{ac-b^2}{2a(1-w^2)}y^2\right\} \frac{1-w^2}{a}\phi\left(\frac{\tau - \frac{b}{a}y}{\sqrt{\frac{1-w^2}{a}}}\right) dy \tag{47}$$

$$+ \int_{-\infty}^{\xi} \frac{\alpha_1}{2\pi\sqrt{1-w^2}} \exp\left\{-\frac{ac-b^2}{2a(1-w^2)}y^2\right\} \frac{b}{a}y\sqrt{\frac{1-w^2}{a}}\Phi\left(\frac{\tau - \frac{b}{a}y}{\sqrt{\frac{1-w^2}{a}}}\right) dy \tag{48}$$

$$+ \int_{-\infty}^{\xi} \frac{a_2 y}{2\pi\sqrt{a}} \exp\left\{-\frac{ac-b^2}{2a(1-w^2)}y^2\right\} \Phi\left(\frac{\tau - \frac{b}{a}y}{\sqrt{\frac{1-w^2}{a}}}\right) dy. \tag{49}$$

Term (47) can be expressed as

$$\int_{-\infty}^{\xi} \frac{\alpha_1\sqrt{1-w^2}}{(2\pi)^{3/2}\,a} \exp\left\{-\frac{ac-b^2}{2a(1-w^2)}y^2\right\} \exp\left\{-\frac{\left(\tau - \frac{b}{a}y\right)^2}{2\frac{1-w^2}{a}}\right\} dy$$

$$= \frac{\alpha_1 1-w^2}{\sqrt{2\pi}a\sqrt{c}}\Phi\left(\frac{c\xi - b\tau}{\sqrt{c(1-w^2)}}\right)\phi\left(\frac{\tau}{\sqrt{\frac{c(1-w^2)}{(ac-b^2)}}}\right),$$

which will be zero if $\tau = \pm\infty$. Term (48) can be expressed as

$$\int_{-\infty}^{\xi} \frac{\alpha_1}{2\pi\sqrt{1-w^2}} \exp\left\{-\frac{ac-b^2}{2a(1-w^2)}y^2\right\} \frac{b}{a}y\sqrt{\frac{1-w^2}{a}}\Phi\left(\frac{\tau - \frac{b}{a}y}{\sqrt{\frac{1-w^2}{a}}}\right) dy$$

$$= \frac{\alpha_1}{\sqrt{2\pi}}\frac{b}{a}\sqrt{\frac{1}{a}}\int_{-\infty}^{\xi} y\phi\left(\frac{y}{\sqrt{\frac{a(1-w^2)}{ac-b^2}}}\right)\Phi\left(\frac{a\tau}{\sqrt{a(1-w^2)}} - \frac{b}{\sqrt{a(1-w^2)}}y\right) dy,$$

which can be solved by (18). Term (49) can be expressed as

$$\int_{-\infty}^{\xi} \frac{a_2 y}{2\pi\sqrt{a}} \exp\left\{-\frac{ac-b^2}{2a\left(1-w^2\right)}y^2\right\} \Phi\left(\frac{\tau-\frac{b}{a}y}{\sqrt{\frac{1-w^2}{a}}}\right) dy$$

$$=\frac{a_2}{\sqrt{2\pi}\sqrt{a}} \int_{-\infty}^{\xi} y\phi\left(\frac{y}{\sqrt{\frac{a(1-w^2)}{ac-b^2}}}\right) \Phi\left(\frac{a\tau}{\sqrt{a\left(1-w^2\right)}} - \frac{b}{\sqrt{a\left(1-w^2\right)}}y\right) dy,$$

which can be solved by (18). Term (45) can be solved by (6) and (26). Term (46) can be solved by (6) and (27).

3.3. Hessian matrix

The Hessian matrix is

$$\boldsymbol{H} = \frac{\partial^2}{\partial\boldsymbol{\theta}\partial\boldsymbol{\theta}^T} \int_{-\infty}^{\tau}\int_{-\infty}^{\xi} f\left(x,y\right) dy dx.$$

The following derivatives will be used,

$$\frac{\partial a}{\partial\boldsymbol{\theta}} = \begin{pmatrix} 2\left(1-w^2\right)\alpha_1 \\ 0 \\ -2\alpha_1^2 w \end{pmatrix}, \quad \frac{\partial b}{\partial\boldsymbol{\theta}} = \begin{pmatrix} -\alpha_2\left(1-w^2\right) \\ -\alpha_1\left(1-w^2\right) \\ 1+2\alpha_1\alpha_2 w \end{pmatrix}, \quad \frac{\partial c}{\partial\boldsymbol{\theta}} = \begin{pmatrix} 0 \\ 2\left(1-w^2\right)\alpha_2 \\ -2\alpha_2^2 w \end{pmatrix}.$$

3.3.1. First column of H

The first column of $\boldsymbol{H}$ is

$$\begin{aligned}
\frac{\partial^2}{\partial\boldsymbol{\theta}\partial\alpha_1}\int_{-\infty}^{\tau}\int_{-\infty}^{\xi} f(x,y)\,dydx = &-\frac{\partial}{\partial\boldsymbol{\theta}}\left[\frac{2\sqrt{1-w^2}}{\sqrt{2\pi}a}\int_{-\infty}^{\xi}\phi\left(\frac{y}{\sqrt{\frac{a(1-w^2)}{ac-b^2}}}\right)\phi\left(\frac{\tau-\frac{b}{a}y}{\sqrt{\frac{1-w^2}{a}}}\right)dy\right]\\
&+\frac{\partial}{\partial\boldsymbol{\theta}}\left[\frac{2}{\sqrt{2\pi}}\frac{b}{a}\sqrt{\frac{1}{a}}\int_{-\infty}^{\xi} y\phi\left(\frac{y}{\sqrt{\frac{a(1-w^2)}{ac-b^2}}}\right)\Phi\left(\frac{\tau-\frac{b}{a}y}{\sqrt{\frac{1-w^2}{a}}}\right)dy\right]\\
&+\frac{\partial}{\partial\boldsymbol{\theta}}\left[\frac{\partial\tau}{\partial\alpha_1}\int_{-\infty}^{\xi}2\phi_2(\tau,y;w)\,\Phi(\alpha_1\tau+\alpha_2 y)\,dy\right]\\
&+\frac{\partial}{\partial\boldsymbol{\theta}}\left[\frac{\partial\xi}{\partial\alpha_1}\int_{-\infty}^{\tau}2\phi_2(x,\xi;w)\,\Phi(\alpha_1 x+\alpha_2\xi)\,dxy\right].
\end{aligned}$$

Firstly,

$$\begin{aligned}
&\frac{\partial}{\partial\boldsymbol{\theta}}\left[\frac{\sqrt{1-w^2}}{a}\int_{-\infty}^{\xi}\phi\left(\frac{y}{\sqrt{\frac{a(1-w^2)}{ac-b^2}}}\right)\phi\left(\frac{\tau-\frac{b}{a}y}{\sqrt{\frac{1-w^2}{a}}}\right)dy\right]\\
&=\left(\frac{\partial}{\partial\boldsymbol{\theta}}\frac{\sqrt{1-w^2}}{a}\right)\int_{-\infty}^{\xi}\phi\left(\frac{y}{\sqrt{\frac{a(1-w^2)}{ac-b^2}}}\right)\phi\left(\frac{a\tau}{\sqrt{a(1-w^2)}}-\frac{b}{\sqrt{a(1-w^2)}}y\right)dy\\
&\quad+\frac{\sqrt{1-w^2}}{a}\left[\frac{\partial}{\partial\boldsymbol{\theta}}\int_{-\infty}^{\xi}\phi\left(\frac{y}{\sqrt{\frac{a(1-w^2)}{ac-b^2}}}\right)\phi\left(\frac{a\tau}{\sqrt{a(1-w^2)}}-\frac{b}{\sqrt{a(1-w^2)}}y\right)dy\right],
\end{aligned}$$

which can be solved by (12) and (30).

Secondly,

$$
\begin{aligned}
&\frac{\partial}{\partial \boldsymbol{\theta}}\left[\frac{2}{\sqrt{2\pi}}\frac{b}{a}\sqrt{\frac{1}{a}}\int_{-\infty}^{\xi} y\phi\left(\frac{y}{\sqrt{\frac{a(1-w^2)}{ac-b^2}}}\right)\Phi\left(\frac{\tau-\frac{b}{a}y}{\sqrt{\frac{1-w^2}{a}}}\right)dy\right]\\
=&\frac{2}{\sqrt{2\pi}}\left(\frac{\partial}{\partial \boldsymbol{\theta}}\frac{b}{a^{1.5}}\right)\int_{-\infty}^{\xi} y\phi\left(\frac{y}{\sqrt{\frac{a(1-w^2)}{ac-b^2}}}\right)\Phi\left(\frac{a\tau}{\sqrt{a\left(1-w^2\right)}}-\frac{b}{\sqrt{a\left(1-w^2\right)}}y\right)dy\\
&+\frac{2}{\sqrt{2\pi}}\frac{b}{a}\sqrt{\frac{1}{a}}\left[\frac{\partial}{\partial \boldsymbol{\theta}}\int_{-\infty}^{\xi} y\phi\left(\frac{y}{\sqrt{\frac{a(1-w^2)}{ac-b^2}}}\right)\Phi\left(\frac{a\tau}{\sqrt{a\left(1-w^2\right)}}-\frac{b}{\sqrt{a\left(1-w^2\right)}}y\right)dy\right],
\end{aligned}
$$

which can be solved by (18) and (31).

Thirdly,

$$
\begin{aligned}
&\frac{\partial}{\partial \boldsymbol{\theta}}\left[\frac{\partial \tau}{\partial \alpha_1}\int_{-\infty}^{\xi} 2\phi_2\left(\tau, y; w\right)\Phi\left(\alpha_1\tau+\alpha_2 y\right)dy\right]\\
=&\frac{\partial}{\partial \boldsymbol{\theta}}\left[\frac{\partial \tau}{\partial \alpha_1}\right]\int_{-\infty}^{\xi} 2\phi_2\left(\tau, y; w\right)\Phi\left(\alpha_1\tau+\alpha_2 y\right)dy+\frac{\partial \tau}{\partial \alpha_1}\frac{\partial}{\partial \boldsymbol{\theta}}\left[\int_{-\infty}^{\xi} 2\phi_2\left(\tau, y; w\right)\Phi\left(\alpha_1\tau+\alpha_2 y\right)dy\right],
\end{aligned}
$$

which can be solved by (35) and (41).

Fourthly,

$$
\begin{aligned}
&\frac{\partial}{\partial \boldsymbol{\theta}}\left[\frac{\partial \xi}{\partial \alpha_1}\int_{-\infty}^{\tau} 2\phi_2\left(x, \xi; w\right)\Phi\left(\alpha_1 x+\alpha_2 \xi\right)dxy\right]\\
=&\frac{\partial}{\partial \boldsymbol{\theta}}\left[\frac{\partial \xi}{\partial \alpha_1}\right]\int_{-\infty}^{\tau} 2\phi_2\left(x, \xi; w\right)\Phi\left(\alpha_1 x+\alpha_2 \xi\right)dx+\frac{\partial \xi}{\partial \alpha_1}\frac{\partial}{\partial \boldsymbol{\theta}}\left[\int_{-\infty}^{\tau} 2\phi_2\left(x, \xi; w\right)\Phi\left(\alpha_1 x+\alpha_2 \xi\right)dx\right],
\end{aligned}
$$

which can be solved by (36) and (40).

3.3.2. Second column of H

The second column of $\boldsymbol{H}$ is

$$\begin{aligned}
&\frac{\partial^2}{\partial\boldsymbol{\theta}\partial\alpha_2}\int_{-\infty}^{\tau}\int_{-\infty}^{\xi} f(x,y)\,dydx \\
=&-\frac{\partial}{\partial\boldsymbol{\theta}}\frac{2\sqrt{1-w^2}}{\sqrt{2\pi}c}\int_{-\infty}^{\tau}\phi\left(\frac{x}{\sqrt{\frac{c(1-w^2)}{ac-b^2}}}\right)\phi\left(\frac{c\xi}{\sqrt{c(1-w^2)}}-\frac{bx}{\sqrt{c(1-w^2)}}\right)dx \\
&+\frac{\partial}{\partial\boldsymbol{\theta}}\frac{2}{\sqrt{2\pi}}\frac{b}{c}\sqrt{\frac{1}{c}}\int_{-\infty}^{\tau}x\phi\left(\frac{x}{\sqrt{\frac{c(1-w^2)}{ac-b^2}}}\right)\Phi\left(\frac{c\xi}{\sqrt{c(1-w^2)}}-\frac{bx}{\sqrt{c(1-w^2)}}\right)dx \\
&+\frac{\partial}{\partial\boldsymbol{\theta}}\left(\frac{\partial\tau}{\partial\alpha_2}\int_{-\infty}^{\xi}2\phi_2(\tau,y;w)\,\Phi(\alpha_1\tau+\alpha_2 y)\,dy\right) \\
&+\frac{\partial}{\partial\boldsymbol{\theta}}\left(\frac{\partial\xi}{\partial\alpha_2}\int_{-\infty}^{\tau}2\phi_2(x,\xi;w)\,\Phi(\alpha_1 x+\alpha_2\xi)\,dx\right).
\end{aligned}$$

Firstly,

$$\begin{aligned}
&\frac{\partial}{\partial\boldsymbol{\theta}}\frac{\sqrt{1-w^2}}{c}\int_{-\infty}^{\tau}\phi\left(\frac{x}{\sqrt{\frac{c(1-w^2)}{ac-b^2}}}\right)\phi\left(\frac{c\xi}{\sqrt{c(1-w^2)}}-\frac{bx}{\sqrt{c(1-w^2)}}\right)dx \\
=&\frac{\partial}{\partial\boldsymbol{\theta}}\left(\frac{\sqrt{1-w^2}}{c}\right)\int_{-\infty}^{\tau}\phi\left(\frac{x}{\sqrt{\frac{c(1-w^2)}{ac-b^2}}}\right)\phi\left(\frac{c\xi}{\sqrt{c(1-w^2)}}-\frac{bx}{\sqrt{c(1-w^2)}}\right)dx \\
&+\frac{\sqrt{1-w^2}}{c}\left(\frac{\partial}{\partial\boldsymbol{\theta}}\int_{-\infty}^{\tau}\phi\left(\frac{x}{\sqrt{\frac{c(1-w^2)}{ac-b^2}}}\right)\phi\left(\frac{c\xi}{\sqrt{c(1-w^2)}}-\frac{bx}{\sqrt{c(1-w^2)}}\right)dx\right),
\end{aligned}$$

which can be solved by (12) and (30).

Secondly,

$$\begin{aligned}
&\frac{\partial}{\partial \boldsymbol{\theta}} \frac{2}{\sqrt{2\pi}} \frac{b}{c} \sqrt{\frac{1}{c}} \int_{-\infty}^{\tau} x \phi \left(\frac{x}{\sqrt{\frac{c(1-w^2)}{ac-b^2}}} \right) \Phi \left(\frac{c\xi}{\sqrt{c\left(1-w^2\right)}} - \frac{bx}{\sqrt{c\left(1-w^2\right)}} \right) dx \\
=& \frac{\partial}{\partial \boldsymbol{\theta}} \left(\frac{2}{\sqrt{2\pi}} \frac{b}{c} \sqrt{\frac{1}{c}} \right) \int_{-\infty}^{\tau} x \phi \left(\frac{x}{\sqrt{\frac{c(1-w^2)}{ac-b^2}}} \right) \Phi \left(\frac{c\xi}{\sqrt{c\left(1-w^2\right)}} - \frac{bx}{\sqrt{c\left(1-w^2\right)}} \right) dx \\
&+ \frac{2}{\sqrt{2\pi}} \frac{b}{c} \sqrt{\frac{1}{c}} \frac{\partial}{\partial \boldsymbol{\theta}} \left(\int_{-\infty}^{\tau} x \phi \left(\frac{x}{\sqrt{\frac{c(1-w^2)}{ac-b^2}}} \right) \Phi \left(\frac{c\xi}{\sqrt{c\left(1-w^2\right)}} - \frac{bx}{\sqrt{c\left(1-w^2\right)}} \right) dx \right),
\end{aligned}$$

which can be solved by (18) and (32).

Thirdly,

$$\begin{aligned}
&\frac{\partial}{\partial \boldsymbol{\theta}} \frac{\partial \tau}{\partial \alpha_2} \int_{-\infty}^{\xi} 2\phi_2\left(\tau, y; w\right) \Phi\left(\alpha_1 \tau + \alpha_2 y\right) dy \\
=& \frac{\partial}{\partial \boldsymbol{\theta}} \left(\frac{\partial \tau}{\partial \alpha_2} \right) \int_{-\infty}^{\xi} 2\phi_2\left(\tau, y; w\right) \Phi\left(\alpha_1 \tau + \alpha_2 y\right) dy + \frac{\partial \tau}{\partial \alpha_2} \left(\frac{\partial}{\partial \boldsymbol{\theta}} \int_{-\infty}^{\xi} 2\phi_2\left(\tau, y; w\right) \Phi\left(\alpha_1 \tau + \alpha_2 y\right) dy \right),
\end{aligned}$$

which can be solved by (35) and (41).

Forth term

$$\begin{aligned}
&\frac{\partial}{\partial \boldsymbol{\theta}} \frac{\partial \xi}{\partial \alpha_2} \int_{-\infty}^{\tau} 2\phi_2\left(x, \xi; w\right) \Phi\left(\alpha_1 x + \alpha_2 \xi\right) dx \\
=& \frac{\partial}{\partial \boldsymbol{\theta}} \left(\frac{\partial \xi}{\partial \alpha_2} \right) \int_{-\infty}^{\tau} 2\phi_2\left(x, \xi; w\right) \Phi\left(\alpha_1 x + \alpha_2 \xi\right) dx + \frac{\partial \xi}{\partial \alpha_2} \frac{\partial}{\partial \boldsymbol{\theta}} \left(\int_{-\infty}^{\tau} 2\phi_2\left(x, \xi; w\right) \Phi\left(\alpha_1 x + \alpha_2 \xi\right) dx \right)
\end{aligned}$$

which can be solved by (35) and (40).

3.3.3. Last column of H

Consider the second order derivative of (44) first.

$$\begin{aligned}
\frac{\partial(44)}{\partial\boldsymbol{\theta}} =& \frac{\partial}{\partial\boldsymbol{\theta}} 2\Phi\left(\alpha_1\tau+\alpha_2\xi\right)\phi_2\left(\tau,\xi;w\right)\\
&-\frac{\partial}{\partial\boldsymbol{\theta}}\int_{-\infty}^{\xi} 2\alpha_2\phi_2\left(\tau,y;w\right)\phi\left(\alpha_1\tau+\alpha_2 y\right)dy-\frac{\partial}{\partial\boldsymbol{\theta}}\int_{-\infty}^{\tau} 2\alpha_1\phi_2\left(x,\xi;w\right)\phi\left(\alpha_1 x+\alpha_2\xi\right)dx\\
&+\frac{\partial}{\partial\boldsymbol{\theta}}2\alpha_1\alpha_2\frac{\alpha_1 1-w^2}{\sqrt{2\pi}a\sqrt{c}}\Phi\left(\frac{c\xi-b\tau}{\sqrt{c\left(1-w^2\right)}}\right)\phi\left(\frac{\tau}{\sqrt{\frac{c(1-w^2)}{(ac-b^2)}}}\right)\\
&-\frac{\partial}{\partial\boldsymbol{\theta}}2\alpha_1\alpha_2\frac{\alpha_1}{\sqrt{2\pi}}\frac{b}{a}\sqrt{\frac{1}{a}}\int_{-\infty}^{\xi} y\phi\left(\frac{y}{\sqrt{\frac{a(1-w^2)}{ac-b^2}}}\right)\Phi\left(\frac{a\tau}{\sqrt{a\left(1-w^2\right)}}-\frac{b}{\sqrt{a\left(1-w^2\right)}}y\right)dy\\
&-\frac{\partial}{\partial\boldsymbol{\theta}}2\alpha_1\alpha_2\frac{a_2}{\sqrt{2\pi}\sqrt{a}}\int_{-\infty}^{\xi} y\phi\left(\frac{y}{\sqrt{\frac{a(1-w^2)}{ac-b^2}}}\right)\Phi\left(\frac{a\tau}{\sqrt{a\left(1-w^2\right)}}-\frac{b}{\sqrt{a\left(1-w^2\right)}}y\right)dy.
\end{aligned}$$

Firstly,

$$\begin{aligned}
&\frac{\partial}{\partial\boldsymbol{\theta}}2\Phi\left(\alpha_1\tau+\alpha_2\xi\right)\phi_2\left(\tau,\xi;w\right)\\
=&2\phi\left(\alpha_1\tau+\alpha_2\xi\right)\left(\frac{\partial\alpha_1}{\partial\boldsymbol{\theta}}\tau+\alpha_1\frac{\partial\tau}{\partial\boldsymbol{\theta}}+\frac{\partial\alpha_2}{\partial\boldsymbol{\theta}}\xi+\alpha_2\frac{\partial\xi}{\partial\boldsymbol{\theta}}\right)\phi_2\left(\tau,\xi;w\right)+2\Phi\left(\alpha_1\tau+\alpha_2\xi\right)\frac{\partial}{\partial\boldsymbol{\theta}}\phi_2\left(\tau,\xi;w\right),
\end{aligned}$$

which can be solved by (26), (27), and (39).

Secondly,

$$
\begin{aligned}
&\frac{\partial}{\partial \boldsymbol{\theta}} \int_{-\infty}^{\xi} 2\alpha_2 \phi_2 \left(\tau, y; w\right) \phi \left(\alpha_1 \tau + \alpha_2 y\right) dy \\
=&\frac{\partial \alpha_2}{\partial \boldsymbol{\theta}} \int_{-\infty}^{\xi} 2\phi_2 \left(\tau, y; w\right) \phi \left(\alpha_1 \tau + \alpha_2 y\right) dy \\
&+ \alpha_2 \frac{\partial}{\partial \boldsymbol{\theta}} \left[\frac{2}{\sqrt{2\pi \left(1 - w^2\right)}} \right] \exp\left\{ -\frac{\tau^2}{2} \right\} \int_{-\infty}^{\xi - w\tau} \phi \left(\frac{y}{\sqrt{1 - w^2}} \right) \phi \left(\alpha_1 \tau + \alpha_2 w \tau + \alpha_2 y\right) dy \\
&+ \alpha_2 \frac{2}{\sqrt{2\pi \left(1 - w^2\right)}} \frac{\partial}{\partial \boldsymbol{\theta}} \left[\exp\left\{ -\frac{\tau^2}{2} \right\} \right] \int_{-\infty}^{\xi - w\tau} \phi \left(\frac{y}{\sqrt{1 - w^2}} \right) \phi \left(\alpha_1 \tau + \alpha_2 w \tau + \alpha_2 y\right) dy \\
&+ \alpha_2 \frac{2}{\sqrt{2\pi \left(1 - w^2\right)}} \exp\left\{ -\frac{\tau^2}{2} \right\} \frac{\partial}{\partial \boldsymbol{\theta}} \left[\int_{-\infty}^{\xi - w\tau} \phi \left(\frac{y}{\sqrt{1 - w^2}} \right) \phi \left(\alpha_1 \tau + \alpha_2 w \tau + \alpha_2 y\right) dy \right],
\end{aligned}
$$

which can be solved by (26), (27), and (30).

Thirdly,

$$\begin{aligned}
&\frac{\partial}{\partial \boldsymbol{\theta}} \int_{-\infty}^{\tau} 2\alpha_1 \phi_2\left(x, \xi; w\right) \phi\left(\alpha_1 x + \alpha_2 \xi\right) dx \\
=&\frac{\partial \alpha_1}{\partial \boldsymbol{\theta}} \int_{-\infty}^{\tau} 2\phi_2\left(x, \xi; w\right) \phi\left(\alpha_1 x + \alpha_2 \xi\right) dx \\
&+ \alpha_1 \frac{\partial}{\partial \boldsymbol{\theta}} \left[\frac{2}{\sqrt{2\pi\left(1-w^2\right)}}\right] \exp\left\{-\frac{\xi^2}{2}\right\} \int_{-\infty}^{\tau - w\xi} \phi\left(\frac{x}{\sqrt{1-w^2}}\right) \phi\left(\alpha_1 w \xi + \alpha_2 \xi - \alpha_1 x\right) dx \\
&+ \alpha_1 \frac{2}{\sqrt{2\pi\left(1-w^2\right)}} \frac{\partial}{\partial \boldsymbol{\theta}} \left[\exp\left\{-\frac{\xi^2}{2}\right\}\right] \int_{-\infty}^{\tau - w\xi} \phi\left(\frac{x}{\sqrt{1-w^2}}\right) \phi\left(\alpha_1 w \xi + \alpha_2 \xi - \alpha_1 x\right) dx \\
&+ \alpha_1 \frac{2}{\sqrt{2\pi\left(1-w^2\right)}} \exp\left\{-\frac{\xi^2}{2}\right\} \frac{\partial}{\partial \boldsymbol{\theta}} \left[\int_{-\infty}^{\tau - w\xi} \phi\left(\frac{x}{\sqrt{1-w^2}}\right) \phi\left(\alpha_1 w \xi + \alpha_2 \xi - \alpha_1 x\right) dx\right],
\end{aligned}$$

which can be solved by (26), (27), and (30).

Fourthly,

$$\begin{aligned}
&\frac{\partial}{\partial \boldsymbol{\theta}} 2\alpha_1\alpha_2 \frac{\alpha_1\left(1-w^2\right)}{\sqrt{2\pi} a \sqrt{c}} \Phi\left(\frac{c\xi - b\tau}{\sqrt{c\left(1-w^2\right)}}\right) \phi\left(\frac{\tau}{\sqrt{\frac{c(1-w^2)}{(ac-b^2)}}}\right) \\
=&\frac{\partial}{\partial \boldsymbol{\theta}} \left(\frac{2\alpha_1^2\alpha_2\left(1-w^2\right)}{\sqrt{2\pi} a \sqrt{c}}\right) \Phi\left(\frac{c\xi - b\tau}{\sqrt{c\left(1-w^2\right)}}\right) \phi\left(\frac{\tau}{\sqrt{\frac{c(1-w^2)}{(ac-b^2)}}}\right) \\
&+ \frac{2\alpha_1^2\alpha_2\left(1-w^2\right)}{\sqrt{2\pi} a \sqrt{c}} \phi\left(\frac{c\xi - b\tau}{\sqrt{c\left(1-w^2\right)}}\right) \frac{\partial}{\partial \boldsymbol{\theta}} \left(\frac{c\xi - b\tau}{\sqrt{c\left(1-w^2\right)}}\right) \phi\left(\frac{\tau}{\sqrt{\frac{c(1-w^2)}{(ac-b^2)}}}\right) \\
&- \frac{2\alpha_1^2\alpha_2\left(1-w^2\right)}{\sqrt{2\pi} a \sqrt{c}} \Phi\left(\frac{c\xi - b\tau}{\sqrt{c\left(1-w^2\right)}}\right) \frac{\tau}{\sqrt{\frac{c(1-w^2)}{(ac-b^2)}}} \phi\left(\frac{\tau}{\sqrt{\frac{c(1-w^2)}{(ac-b^2)}}}\right) \frac{\partial}{\partial \boldsymbol{\theta}} \left(\frac{\tau}{\sqrt{\frac{c(1-w^2)}{(ac-b^2)}}}\right),
\end{aligned}$$

which can be solved by (26) and (27).

Fifthly,

$$\begin{aligned}
&\frac{\partial}{\partial \boldsymbol{\theta}} 2\alpha_1\alpha_2 \frac{\alpha_1}{\sqrt{2\pi}} \frac{b}{a}\sqrt{\frac{1}{a}} \int_{-\infty}^{\xi} y\phi\left(\frac{y}{\sqrt{\frac{a(1-w^2)}{ac-b^2}}}\right)\Phi\left(\frac{a\tau}{\sqrt{a\left(1-w^2\right)}} - \frac{b}{\sqrt{a\left(1-w^2\right)}}y\right)dy \\
=&\frac{\partial}{\partial \boldsymbol{\theta}}\left(\frac{2\alpha_1^2\alpha_2}{\sqrt{2\pi}} \frac{b}{a}\sqrt{\frac{1}{a}}\right) \int_{-\infty}^{\xi} y\phi\left(\frac{y}{\sqrt{\frac{a(1-w^2)}{ac-b^2}}}\right)\Phi\left(\frac{a\tau}{\sqrt{a\left(1-w^2\right)}} - \frac{b}{\sqrt{a\left(1-w^2\right)}}y\right)dy \\
&+\frac{2\alpha_1^2\alpha_2}{\sqrt{2\pi}} \frac{b}{a}\sqrt{\frac{1}{a}}\frac{\partial}{\partial \boldsymbol{\theta}} \int_{-\infty}^{\xi} y\phi\left(\frac{y}{\sqrt{\frac{a(1-w^2)}{ac-b^2}}}\right)\Phi\left(\frac{a\tau}{\sqrt{a\left(1-w^2\right)}} - \frac{b}{\sqrt{a\left(1-w^2\right)}}y\right)dy,
\end{aligned}$$

which can be solved by (18) and (32).

Sixthly,

$$\begin{aligned}
&\frac{\partial}{\partial \boldsymbol{\theta}} 2\alpha_1\alpha_2 \frac{a_2}{\sqrt{2\pi}\sqrt{a}} \int_{-\infty}^{\xi} y\phi\left(\frac{y}{\sqrt{\frac{a(1-w^2)}{ac-b^2}}}\right)\Phi\left(\frac{a\tau}{\sqrt{a\left(1-w^2\right)}} - \frac{b}{\sqrt{a\left(1-w^2\right)}}y\right)dy \\
=&\frac{\partial}{\partial \boldsymbol{\theta}}\left(\frac{2\alpha_1\alpha_2^2}{\sqrt{2\pi}\sqrt{a}}\right) \int_{-\infty}^{\xi} y\phi\left(\frac{y}{\sqrt{\frac{a(1-w^2)}{ac-b^2}}}\right)\Phi\left(\frac{a\tau}{\sqrt{a\left(1-w^2\right)}} - \frac{b}{\sqrt{a\left(1-w^2\right)}}y\right)dy \\
&+\frac{2\alpha_1\alpha_2^2}{\sqrt{2\pi}\sqrt{a}}\frac{\partial}{\partial \boldsymbol{\theta}} \int_{-\infty}^{\xi} y\phi\left(\frac{y}{\sqrt{\frac{a(1-w^2)}{ac-b^2}}}\right)\Phi\left(\frac{a\tau}{\sqrt{a\left(1-w^2\right)}} - \frac{b}{\sqrt{a\left(1-w^2\right)}}y\right)dy,
\end{aligned}$$

which can be solved by (18) and (32).

Then consider term (45).

$$\frac{\partial(45)}{\partial \boldsymbol{\theta}} =\frac{\partial}{\partial \boldsymbol{\theta}}\left(\frac{\partial \tau}{\partial w}\right)\int_{-\infty}^{\xi} 2\phi_2\left(\tau, y; w\right)\Phi\left(\alpha_1\tau+\alpha_2 y\right)dy + \frac{\partial \tau}{\partial w}\frac{\partial}{\partial \boldsymbol{\theta}}\left[\int_{-\infty}^{\xi} 2\phi_2\left(\tau, y; w\right)\Phi\left(\alpha_1\tau+\alpha_2 y\right)dy\right],$$

which can be solved by (35) and (38).

At last consider term (46).

$$\frac{\partial(46)}{\partial\boldsymbol{\theta}} = \frac{\partial}{\partial\boldsymbol{\theta}}\left(\frac{\partial\xi}{\partial w}\right)\int_{-\infty}^{\tau} 2\phi_2\left(x,\xi;w\right)\Phi\left(\alpha_1 x+\alpha_2\xi\right)dx + \frac{\partial\xi}{\partial w}\frac{\partial}{\partial\boldsymbol{\theta}}\left[\int_{-\infty}^{\tau} 2\phi_2\left(x,\xi;w\right)\Phi\left(\alpha_1 x+\alpha_2\xi\right)dx\right],$$

which can be solved by (36) and (37).

References

Azzalini, A., & Capitanio, A. (2014). The skew-normal and related families. *Institute of Mathematical Statistics Monographs (No. 3)*, Cambridge: Cambridge University Press.

Azzalini, A., & Valle, A. D. (1996). The multivariate skew-normal distribution. *Biometrika*, *83*, 715-726.

Balakrishnan, N., & Lai, C. D. (2009). *Continuous bivariate distributions* (2nd ed.). New York, NY: Springer.

4. Extra Plots

In this section, figures that are not included in the main text are displayed.

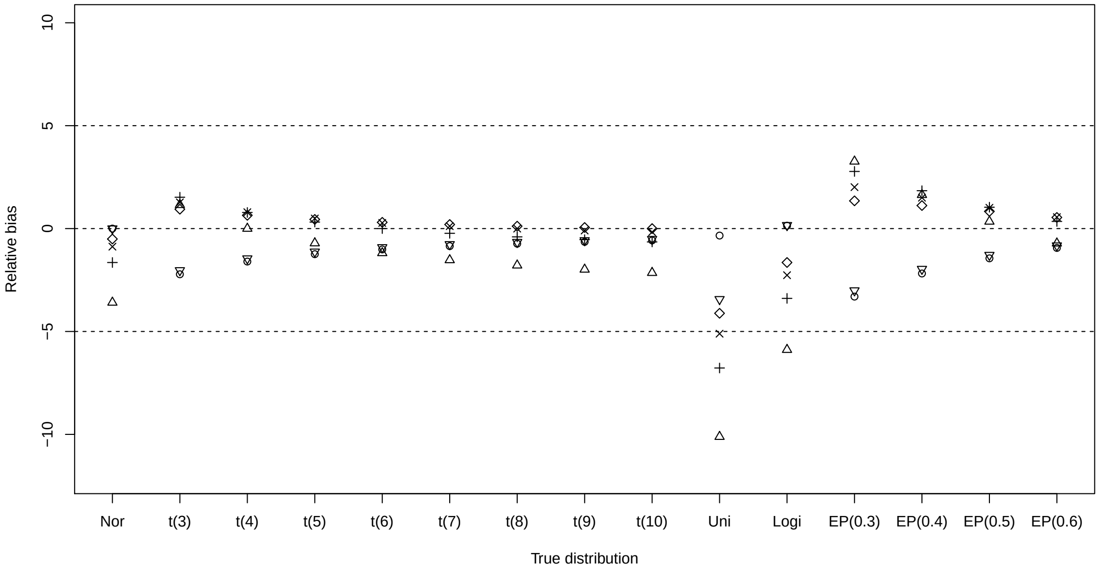

(a) RB

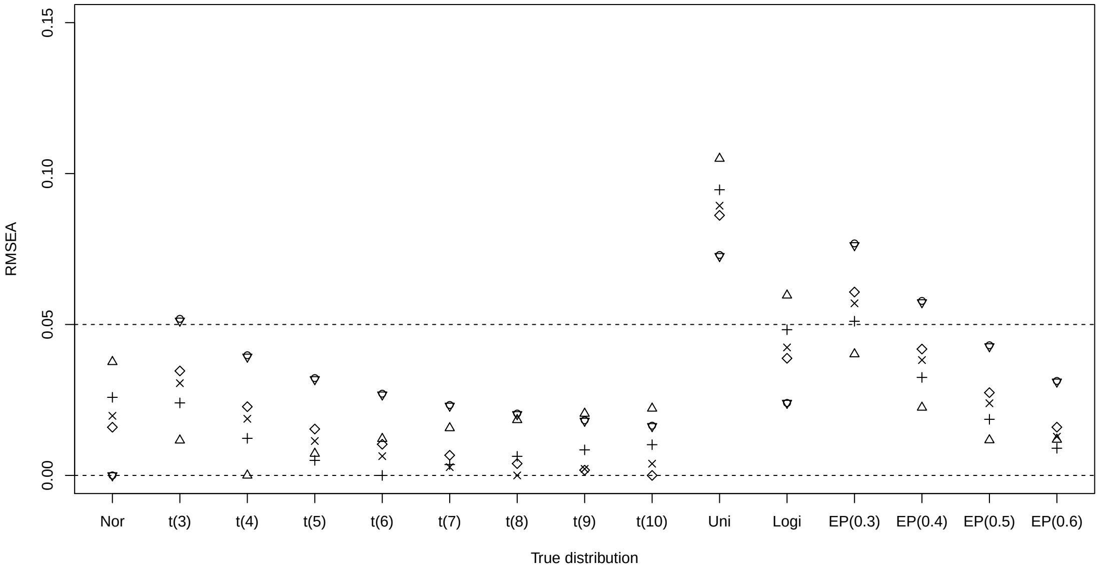

(b) RMSEA

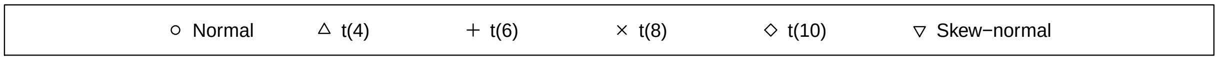

Figure 1: Relative bias (RB) and root mean square error of approximation (RMSEA) of correlation estimates when the true underlying distribution belongs to the elliptical distribution family. Both ordinal variables have five categories. The true correlation coefficient is 0.4. Note: Nor=normal, Uni=uniform, Logi=logistic, EP($\cdot$)=exponential power distribution with the enclosed value of β.

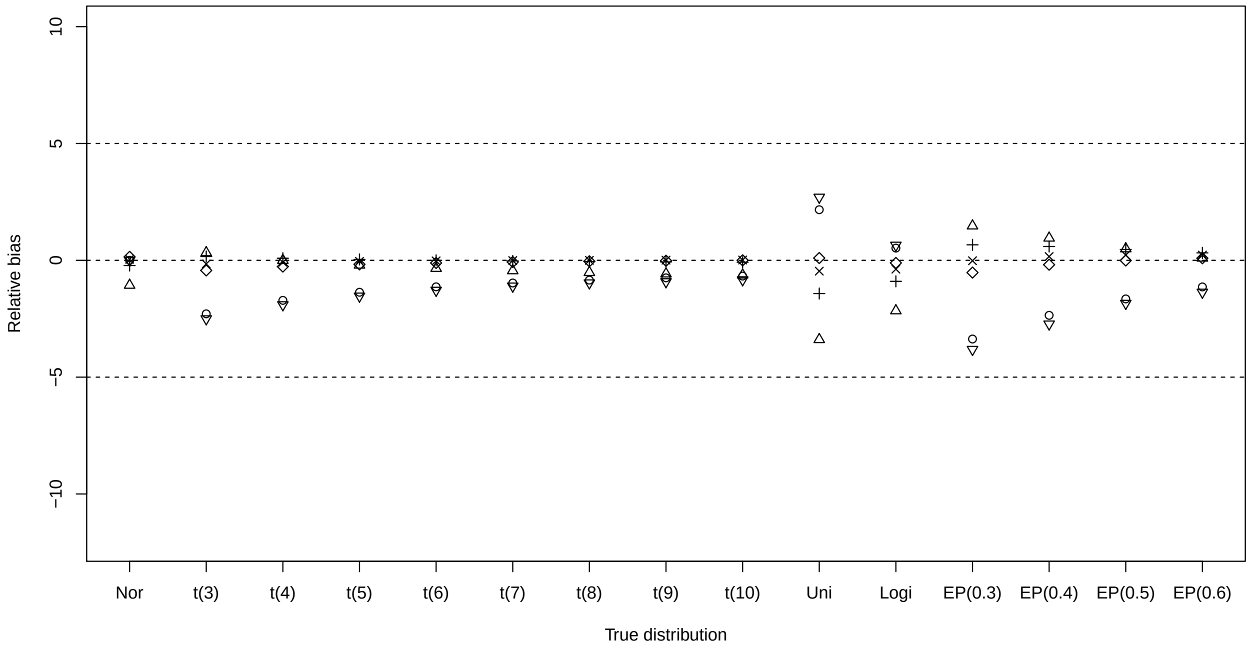

(a) RB

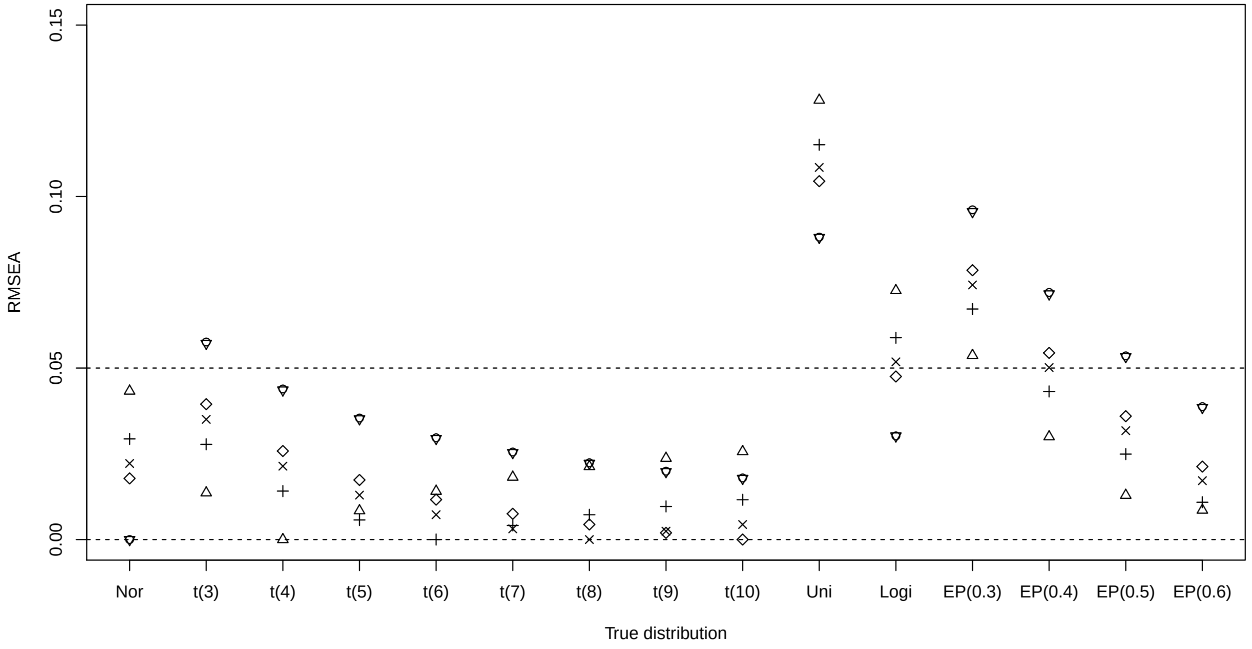

(b) RMSEA

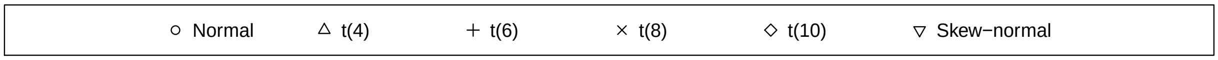

Figure 2: Relative bias (RB) and root mean square error of approximation (RMSEA) of correlation estimates when the true underlying distribution belongs to the elliptical distribution family. The first ordinal variable has three categories and the second ordinal variable has five categories. The true correlation coefficient is 0.4.

Note: Nor=normal, Uni=uniform, Logi=logistic, EP($\cdot$)=exponential power distribution with the enclosed value of β.

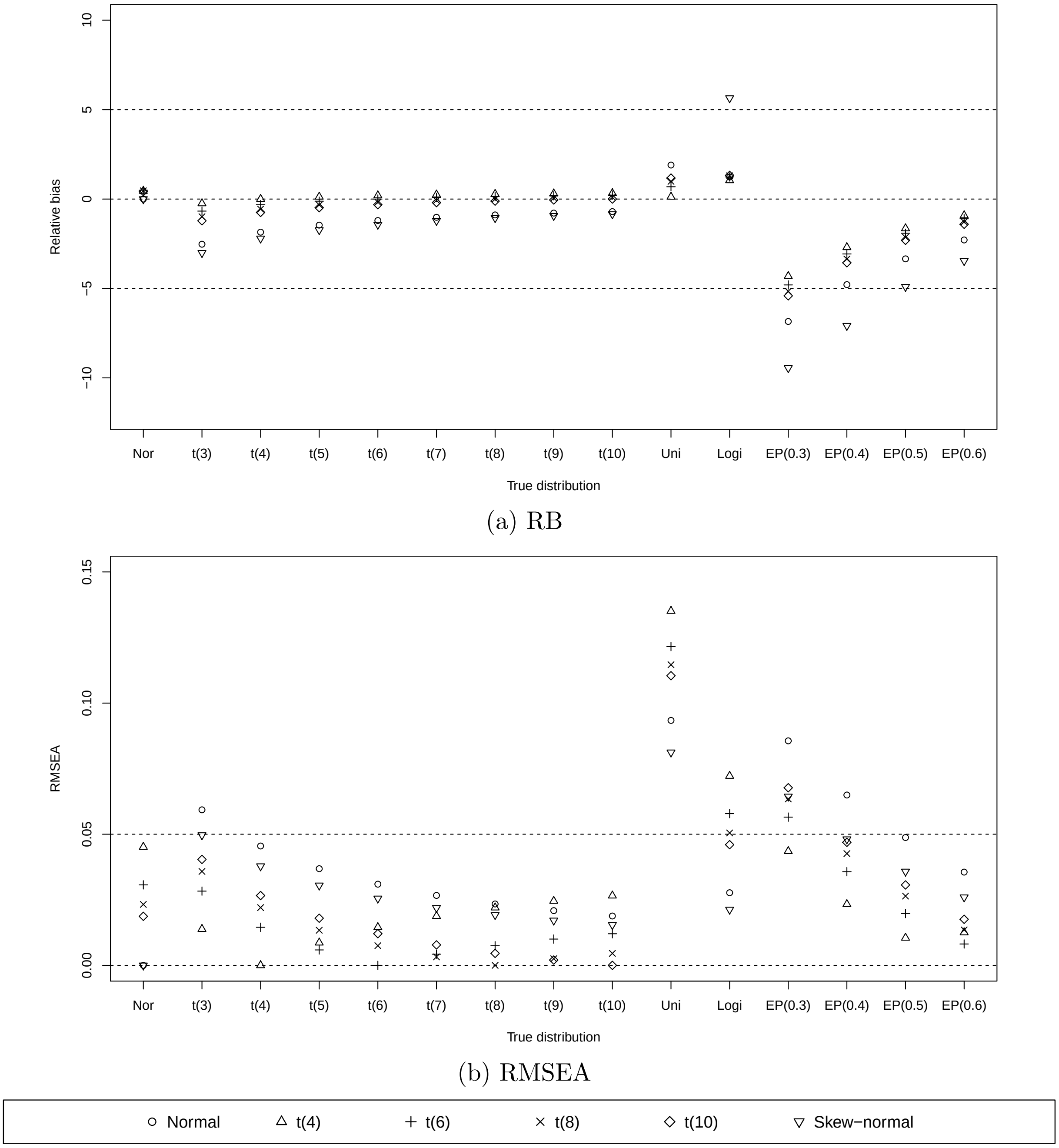

Figure 3: Relative bias (RB) and root mean square error of approximation (RMSEA) of correlation estimates when the true underlying distribution belongs to the elliptical distribution family. Both ordinal variables have three categories. The true correlation coefficient is 0.4. Note: Nor=normal, Uni=uniform, Logi=logistic, EP($\cdot$)=exponential power distribution with the enclosed value of β.

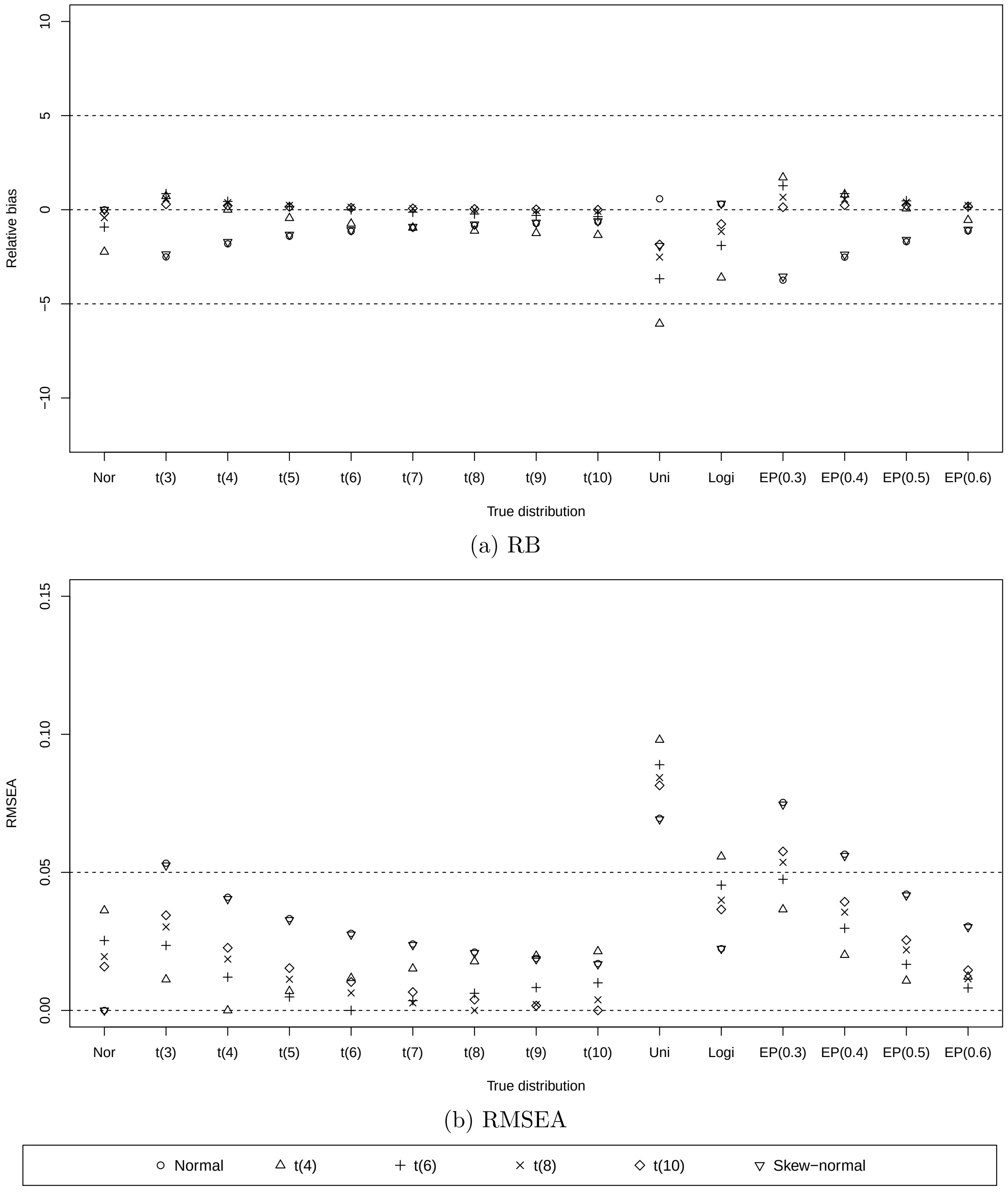

Figure 4: Relative bias (RB) and root mean square error of approximation (RMSEA) of correlation estimates when the true underlying distribution belongs to the elliptical distribution family. Both ordinal variables have five categories. The true correlation coefficient is 0.6. Note: Nor=normal, Uni=uniform, Logi=logistic, EP(·)=exponential power distribution with the enclosed value of β.

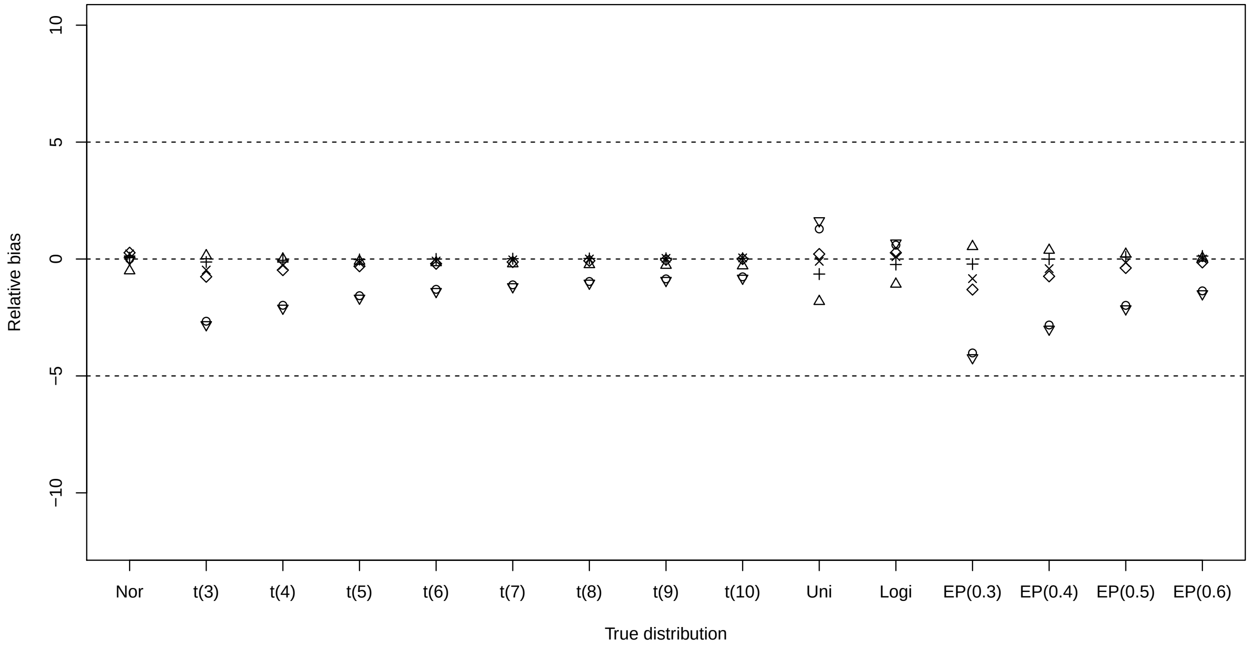

(a) RB

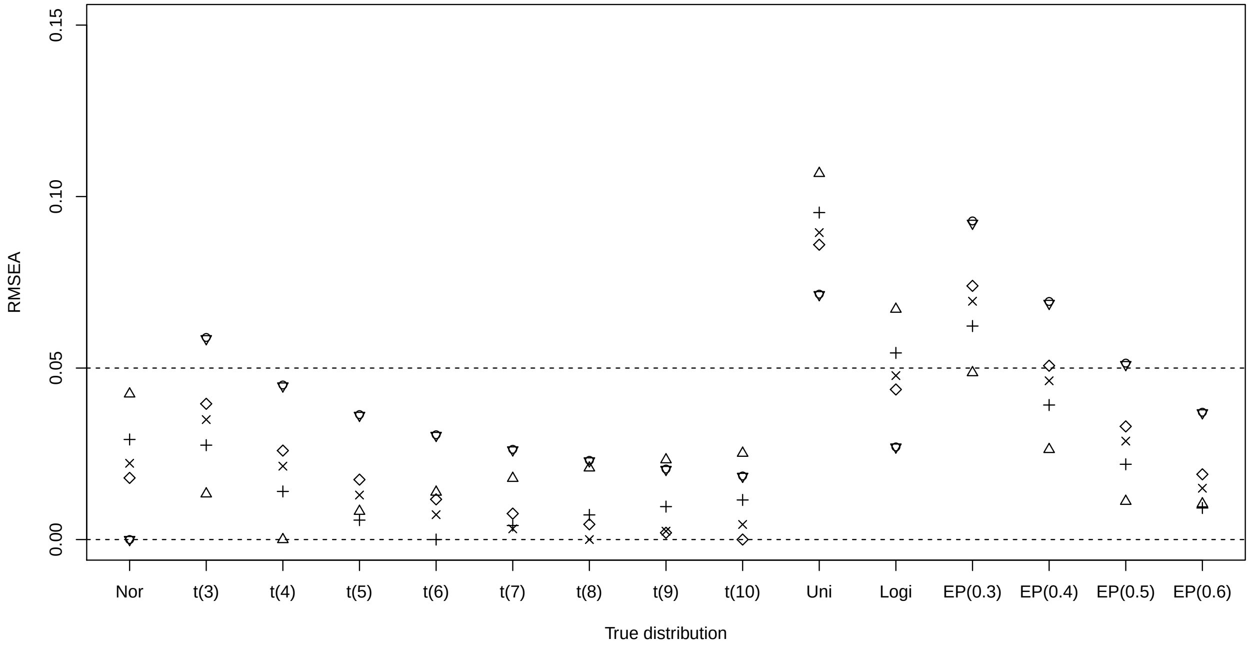

(b) RMSEA

Figure 5: Relative bias (RB) and root mean square error of approximation (RMSEA) of correlation estimates when the true underlying distribution belongs to the elliptical distribution family. The first ordinal variable has three categories and the second ordinal variable has five categories. The true correlation coefficient is 0.6.
Note: Nor=normal, Uni=uniform, Logi=logistic, EP($\cdot$)=exponential power distribution with the enclosed value of β.

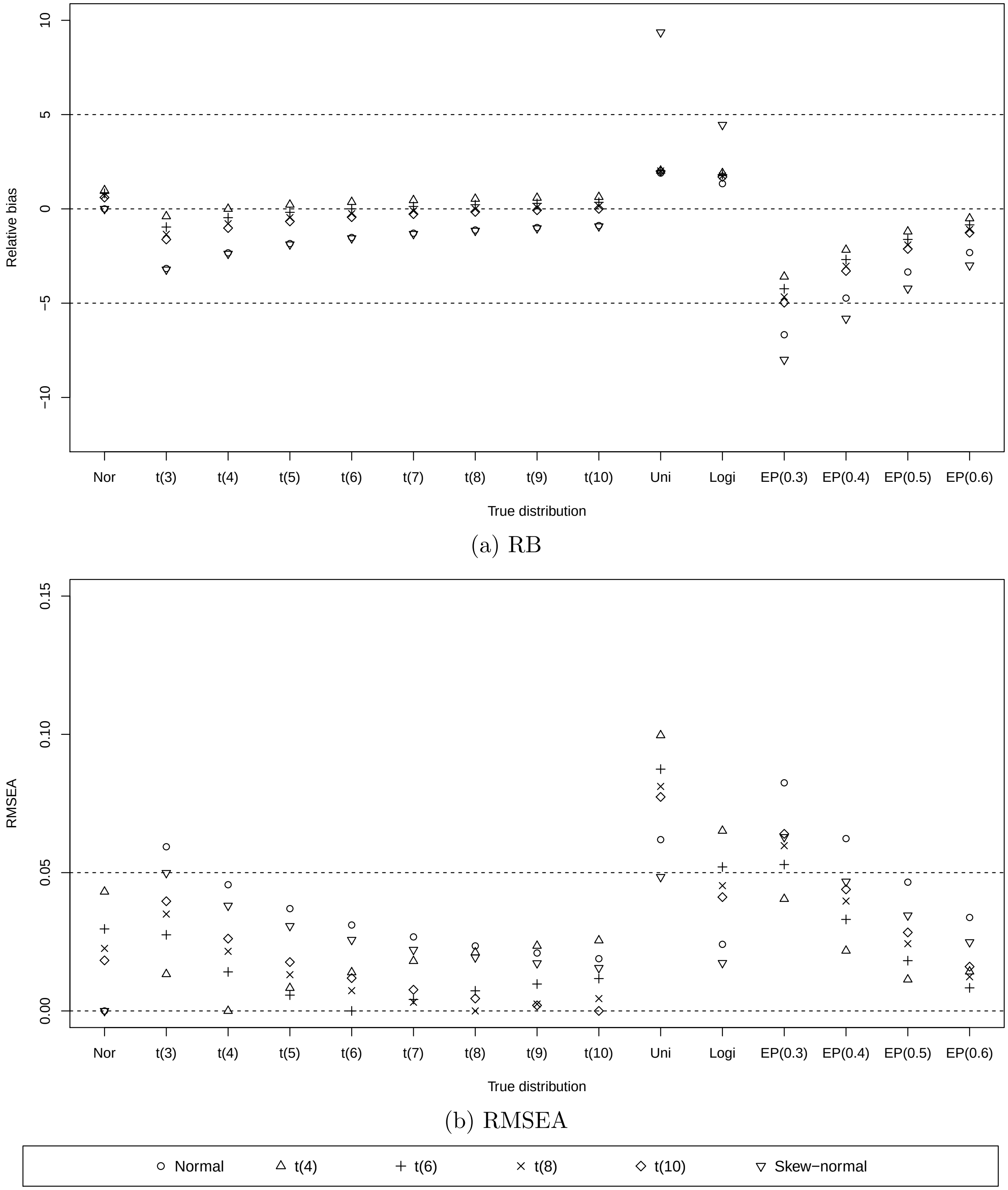

Figure 6: Relative bias (RB) and root mean square error of approximation (RMSEA) of correlation estimates when the true underlying distribution belongs to the elliptical distribution family. Both ordinal variables have three categories. The true correlation coefficient is 0.6. Note: Nor=normal, Uni=uniform, Logi=logistic, EP($\cdot$)=exponential power distribution with the enclosed value of β.

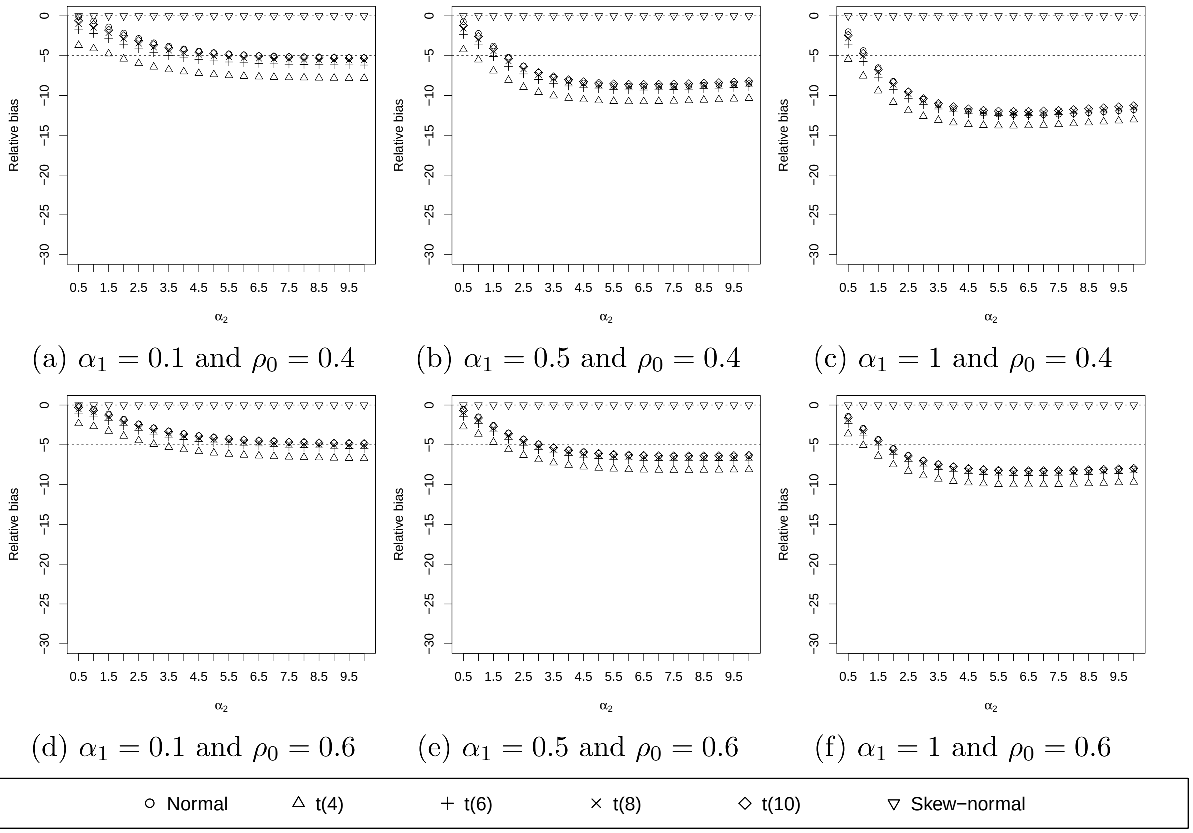

(a) $\alpha_1 = 0.1$ and $\rho_0 = 0.4$ (b) $\alpha_1 = 0.5$ and $\rho_0 = 0.4$ (c) $\alpha_1 = 1$ and $\rho_0 = 0.4$

(d) $\alpha_1 = 0.1$ and $\rho_0 = 0.6$ (e) $\alpha_1 = 0.5$ and $\rho_0 = 0.6$ (f) $\alpha_1 = 1$ and $\rho_0 = 0.6$

Figure 7: Relative bias (RB) of correlation estimates when the true underlying distribution is skew-normal. Both ordinal variables have five categories.

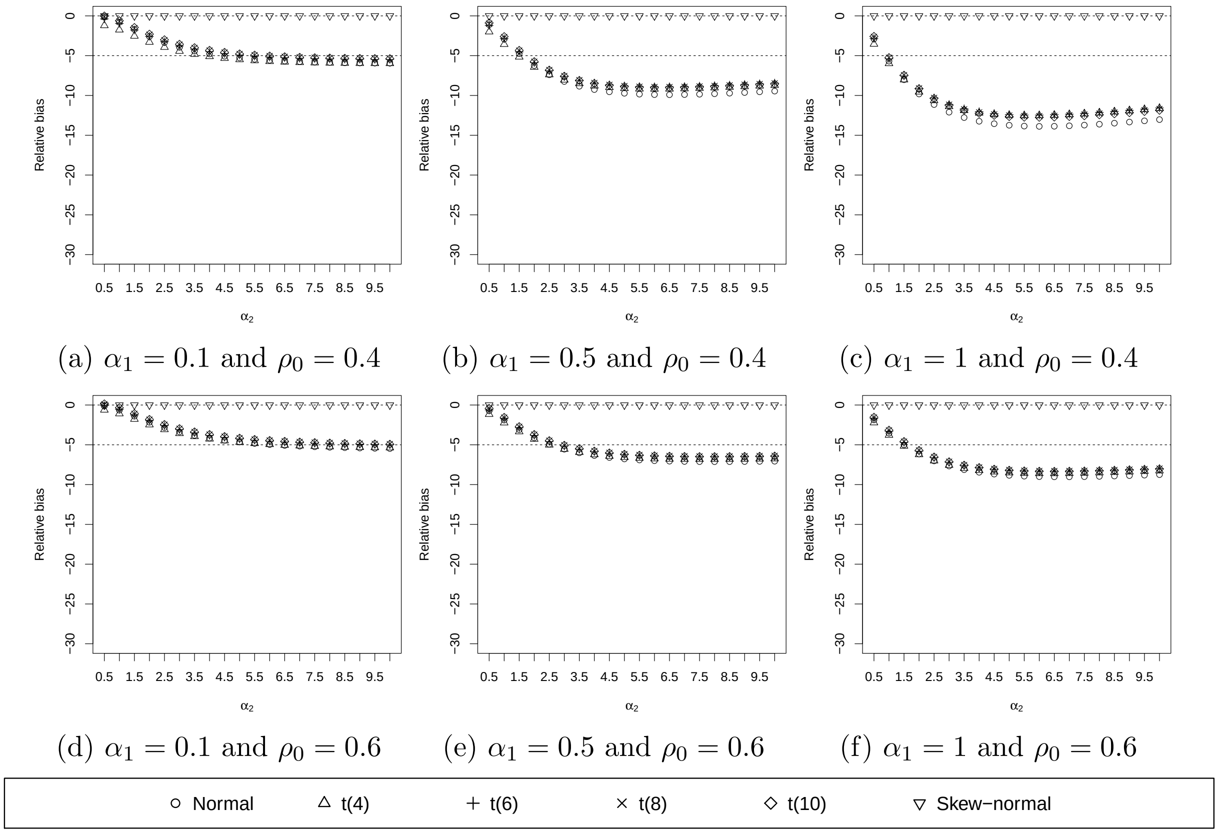

(a) $\alpha_1 = 0.1$ and $\rho_0 = 0.4$ (b) $\alpha_1 = 0.5$ and $\rho_0 = 0.4$ (c) $\alpha_1 = 1$ and $\rho_0 = 0.4$

(d) $\alpha_1 = 0.1$ and $\rho_0 = 0.6$ (e) $\alpha_1 = 0.5$ and $\rho_0 = 0.6$ (f) $\alpha_1 = 1$ and $\rho_0 = 0.6$

Figure 8: Relative bias (RB) of correlation estimates when the true underlying distribution is skew-normal. The first ordinal variable has there categories and the second ordinal variable has five categories.

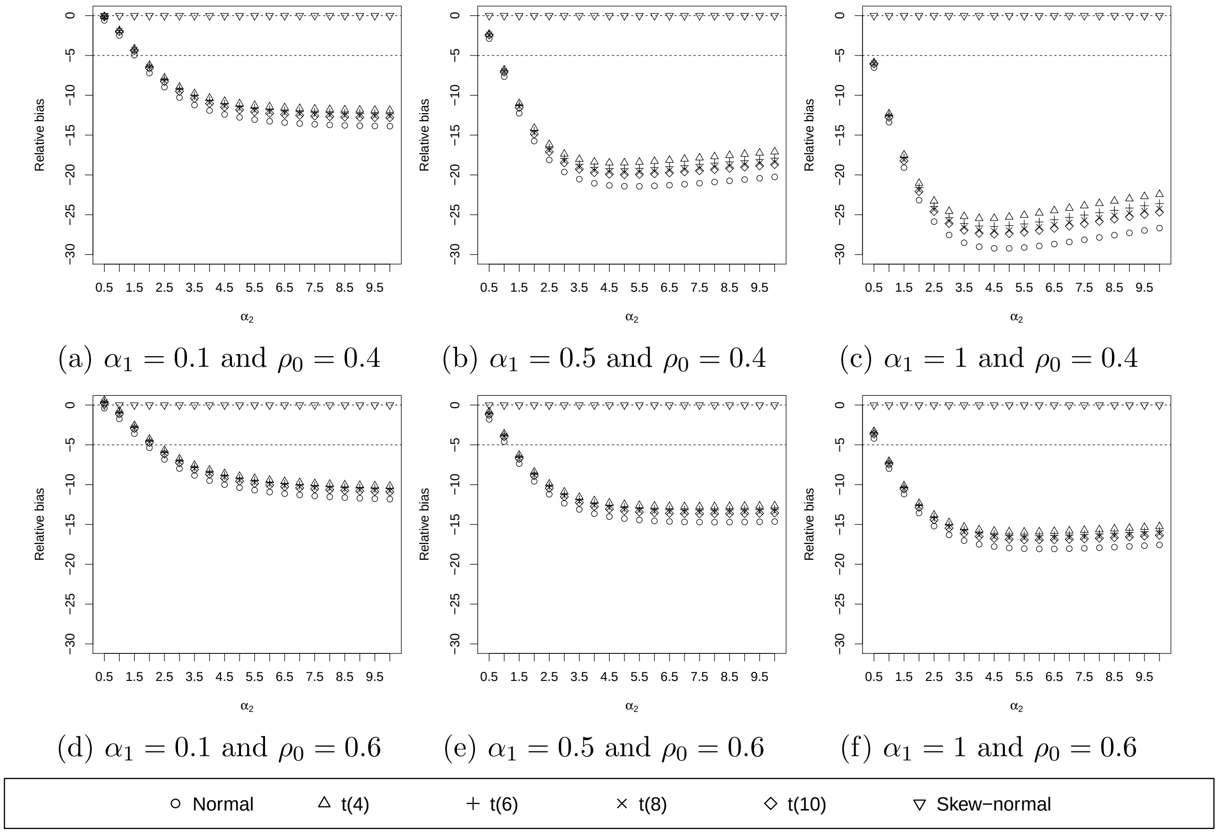

(a) $\alpha_1 = 0.1$ and $\rho_0 = 0.4$ (b) $\alpha_1 = 0.5$ and $\rho_0 = 0.4$ (c) $\alpha_1 = 1$ and $\rho_0 = 0.4$

(d) $\alpha_1 = 0.1$ and $\rho_0 = 0.6$ (e) $\alpha_1 = 0.5$ and $\rho_0 = 0.6$ (f) $\alpha_1 = 1$ and $\rho_0 = 0.6$

Figure 9: Relative bias (RB) of correlation estimates when the true underlying distribution is skew-normal. Both ordinal variables have three categories.

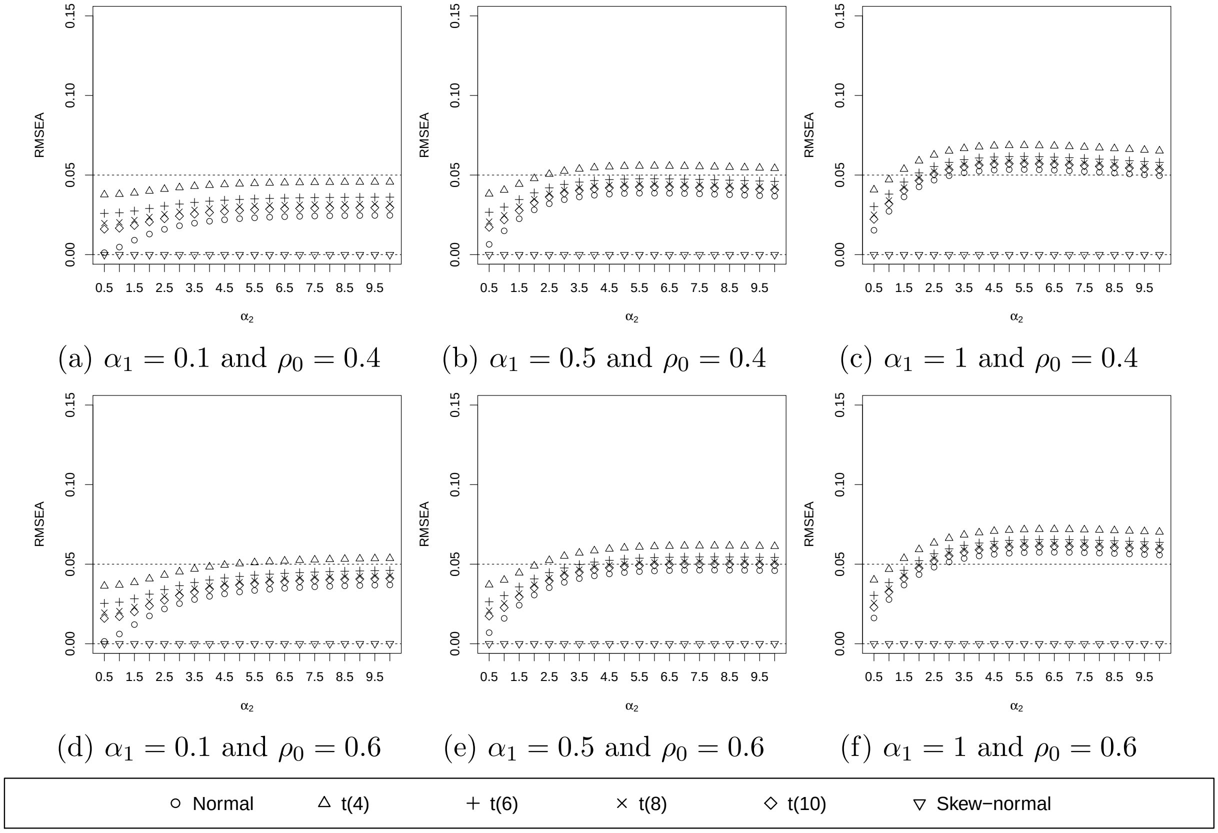

Figure 10: Root mean squared error of approximation (RMSEA) of correlation estimates when the true underlying distribution is skew-normal. Both ordinal variables have five categories.

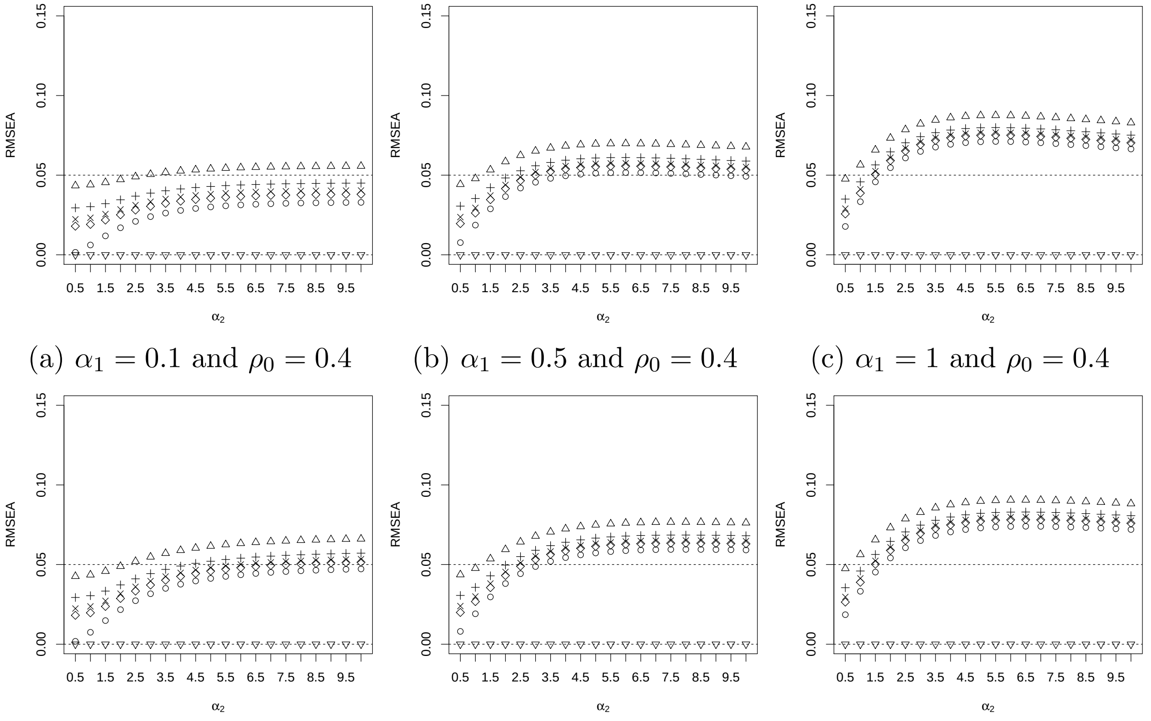

(a) $\alpha_1 = 0.1$ and $\rho_0 = 0.4$ (b) $\alpha_1 = 0.5$ and $\rho_0 = 0.4$ (c) $\alpha_1 = 1$ and $\rho_0 = 0.4$

(d) $\alpha_1 = 0.1$ and $\rho_0 = 0.6$ (e) $\alpha_1 = 0.5$ and $\rho_0 = 0.6$ (f) $\alpha_1 = 1$ and $\rho_0 = 0.6$

○ Normal △ t(4) + t(6) × t(8) ◇ t(10) ▽ Skew−normal

Figure 11: Root mean squared error of approximation (RMSEA) of correlation estimates when the true underlying distribution is skew-normal. The first ordinal variable has there categories and the second ordinal variable has five categories.

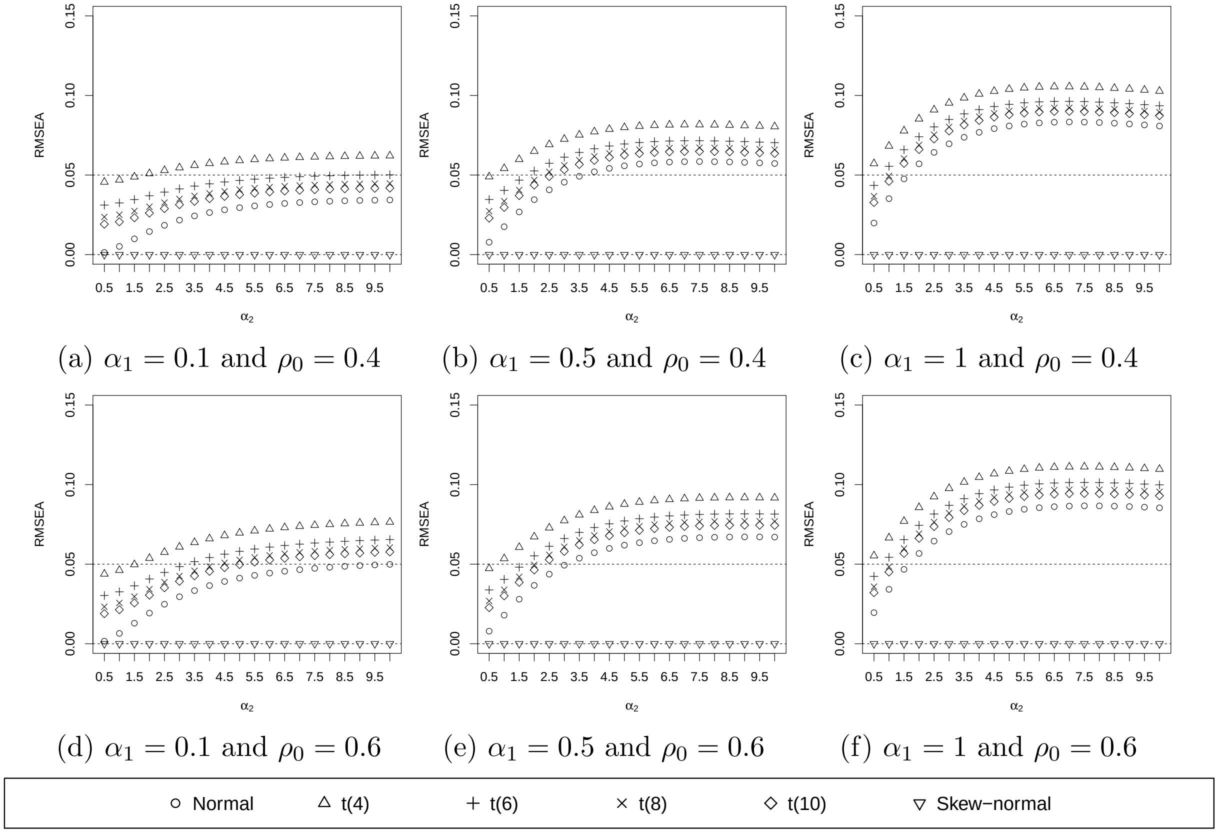

(a) $\alpha_1 = 0.1$ and $\rho_0 = 0.4$ (b) $\alpha_1 = 0.5$ and $\rho_0 = 0.4$ (c) $\alpha_1 = 1$ and $\rho_0 = 0.4$

(d) $\alpha_1 = 0.1$ and $\rho_0 = 0.6$ (e) $\alpha_1 = 0.5$ and $\rho_0 = 0.6$ (f) $\alpha_1 = 1$ and $\rho_0 = 0.6$

Figure 12: Root mean squared error of approximation (RMSEA) of correlation estimates when the true underlying distribution is skew-normal. Both ordinal variables have three categories.

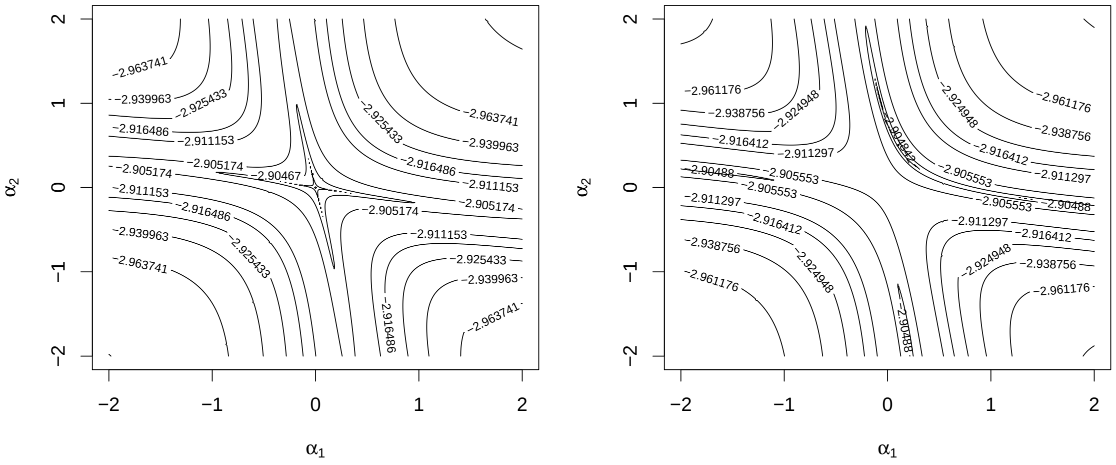

(a) True underlying distribution is standard bivariate normal

(b) True underlying distribution is bivariate skew-normal with $\alpha_1 = 0.1$ and $\alpha_2 = 0.5$

Figure 13: Contour of $\sum_{i=1}^{m_U} \sum_{j=1}^{m_V} p_{ij} \log \pi_{ij,(\mathrm{H})}$ as a function of α_1 and α_2 conditional on the true value of w when $\rho_0 = 0.4$.

Note: In (a) the true values are $\alpha_1 = 0$ and $\alpha_2 = 0$, which are surrounded by nearly local maximizers whose gradient is almost $\mathbf{0}$. In (b) the true values are $\alpha_1 = 0.1$ and $\alpha_2 = 0.5$. However, local maximizers appear when α_1 or α_2 is small.

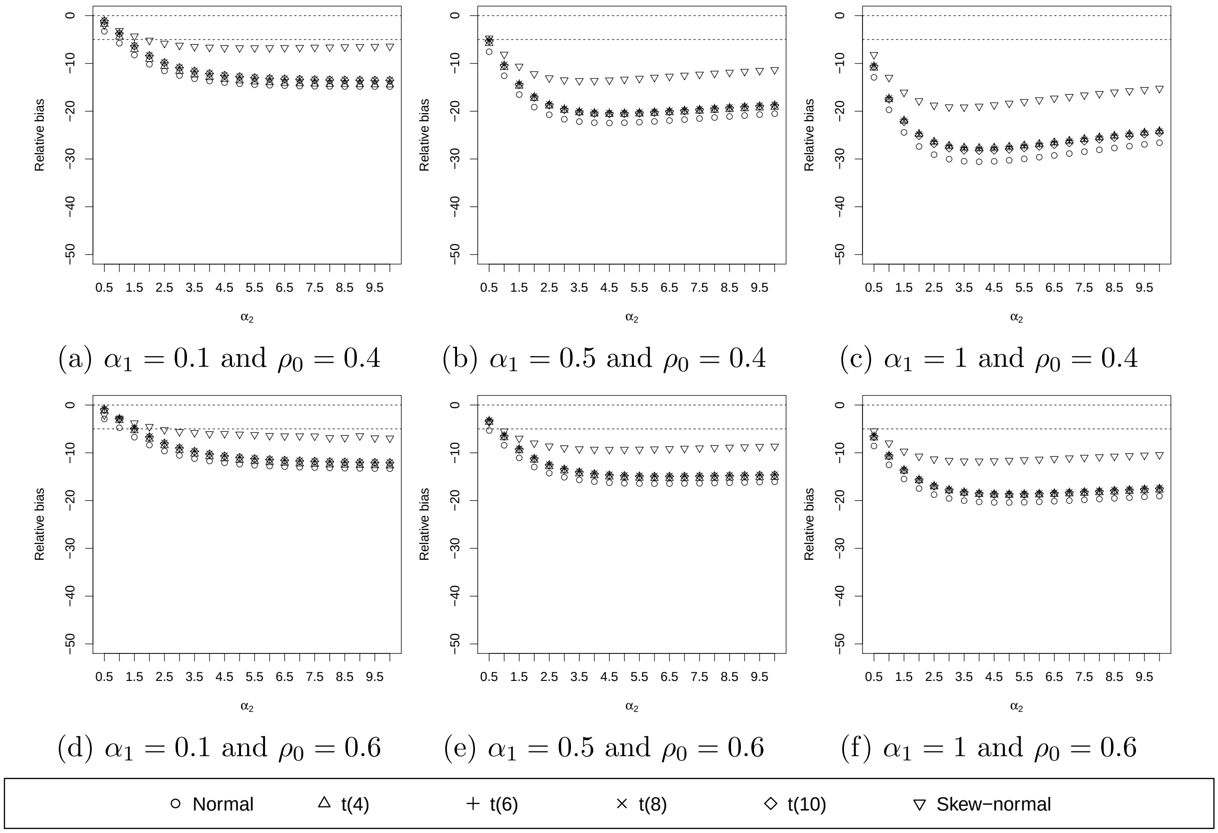

(a) $\alpha_1 = 0.1$ and $\rho_0 = 0.4$ (b) $\alpha_1 = 0.5$ and $\rho_0 = 0.4$ (c) $\alpha_1 = 1$ and $\rho_0 = 0.4$

(d) $\alpha_1 = 0.1$ and $\rho_0 = 0.6$ (e) $\alpha_1 = 0.5$ and $\rho_0 = 0.6$ (f) $\alpha_1 = 1$ and $\rho_0 = 0.6$

Figure 14: Relative bias (RB) of correlation estimates when the true underlying distribution is skew-t(4). Both ordinal variables have five categories.

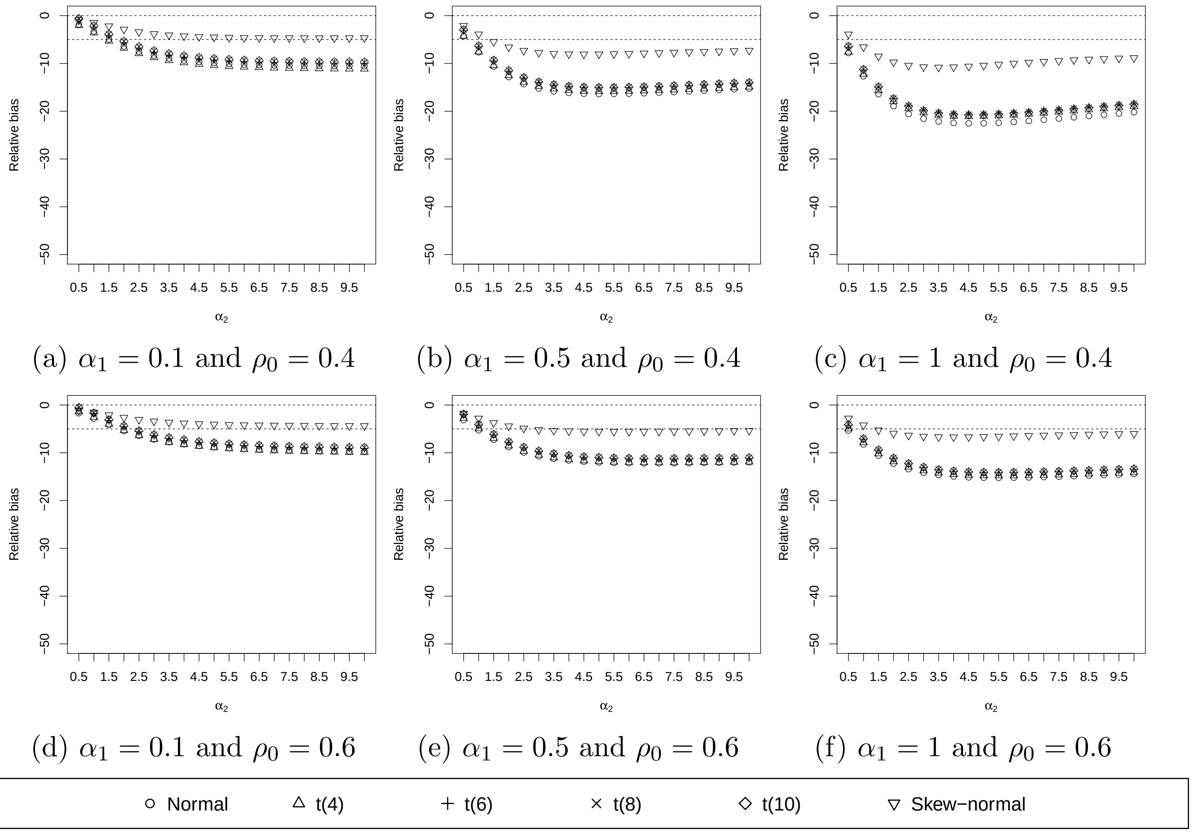

(a) $\alpha_1 = 0.1$ and $\rho_0 = 0.4$ (b) $\alpha_1 = 0.5$ and $\rho_0 = 0.4$ (c) $\alpha_1 = 1$ and $\rho_0 = 0.4$

(d) $\alpha_1 = 0.1$ and $\rho_0 = 0.6$ (e) $\alpha_1 = 0.5$ and $\rho_0 = 0.6$ (f) $\alpha_1 = 1$ and $\rho_0 = 0.6$

Figure 15: Relative bias (RB) of correlation estimates when the true underlying distribution is skew-t(6). Both ordinal variables have five categories.

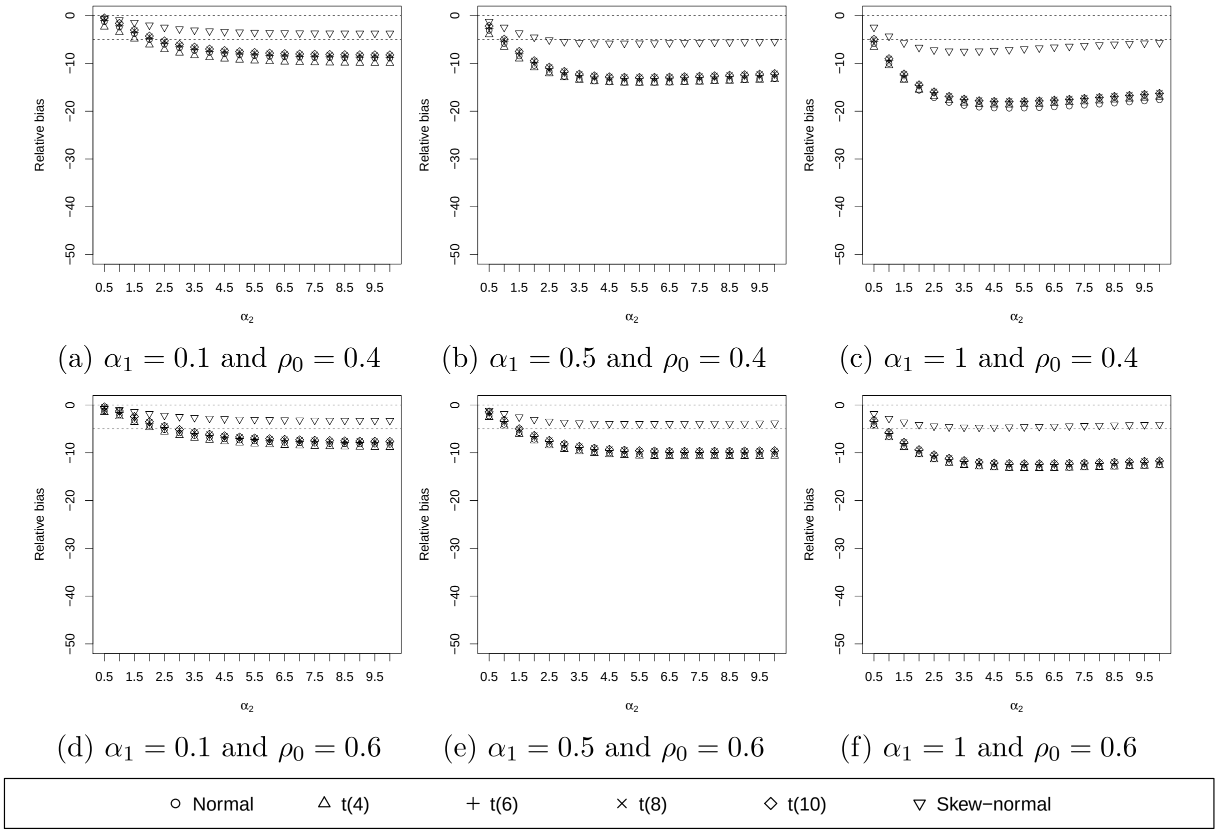

Figure 16: Relative bias (RB) of correlation estimates when the true underlying distribution is skew-t(8). Both ordinal variables have five categories.

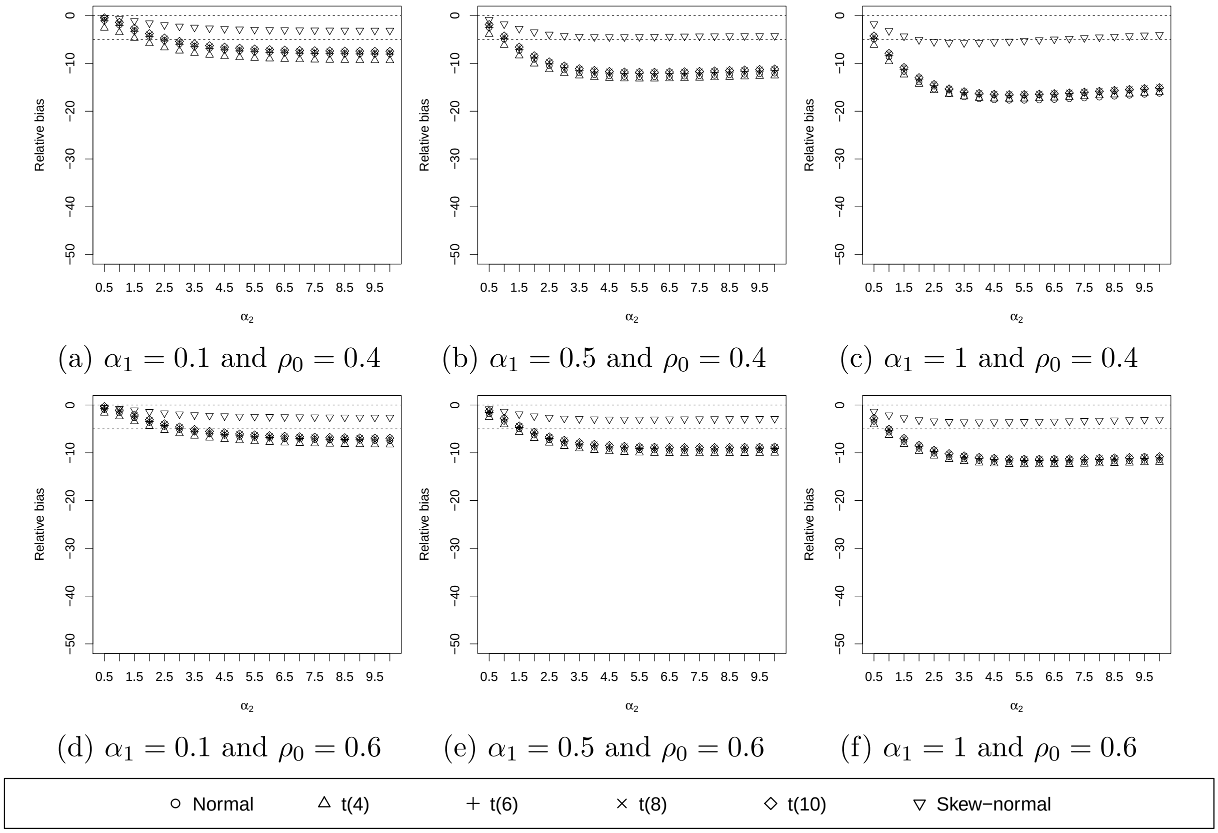

(a) $\alpha_1 = 0.1$ and $\rho_0 = 0.4$ (b) $\alpha_1 = 0.5$ and $\rho_0 = 0.4$ (c) $\alpha_1 = 1$ and $\rho_0 = 0.4$

(d) $\alpha_1 = 0.1$ and $\rho_0 = 0.6$ (e) $\alpha_1 = 0.5$ and $\rho_0 = 0.6$ (f) $\alpha_1 = 1$ and $\rho_0 = 0.6$

Figure 17: Relative bias (RB) of correlation estimates when the true underlying distribution is skew-t(10). Both ordinal variables have five categories.

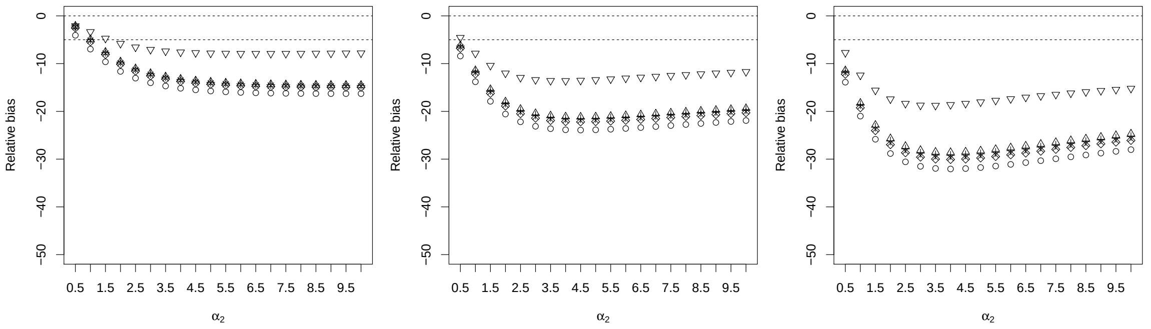

(a) $\alpha_1 = 0.1$ and $\rho_0 = 0.4$ (b) $\alpha_1 = 0.5$ and $\rho_0 = 0.4$ (c) $\alpha_1 = 1$ and $\rho_0 = 0.4$

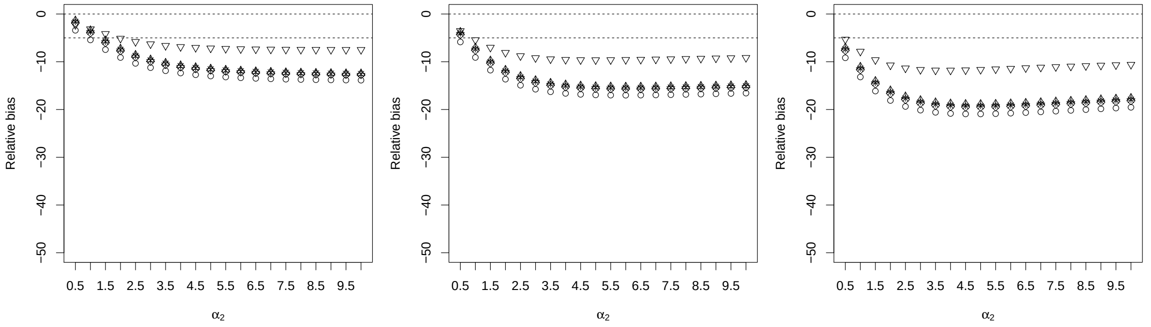

(d) $\alpha_1 = 0.1$ and $\rho_0 = 0.6$ (e) $\alpha_1 = 0.5$ and $\rho_0 = 0.6$ (f) $\alpha_1 = 1$ and $\rho_0 = 0.6$

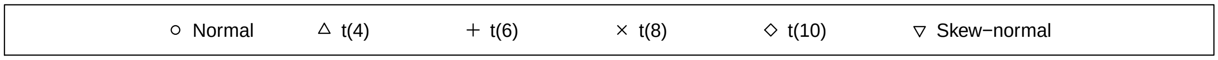

Figure 18: Relative bias (RB) of correlation estimates when the true underlying distribution is skew-t(4). The first ordinal variable has three categories and the second ordinal variable has five categories.

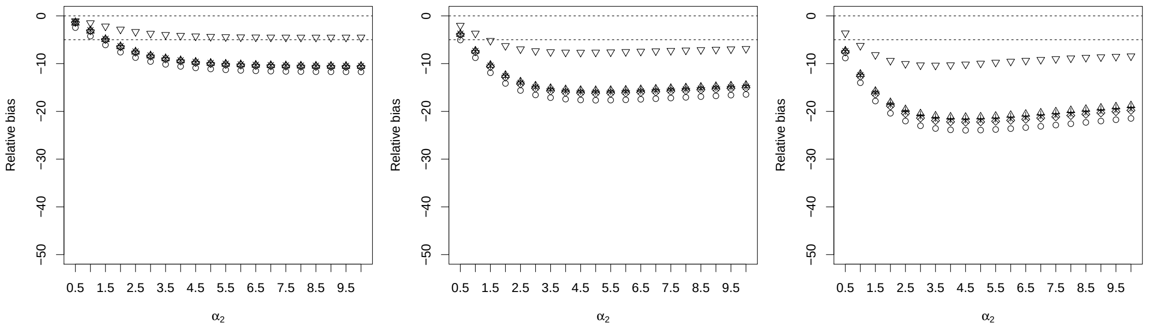

(a) $\alpha_1 = 0.1$ and $\rho_0 = 0.4$ (b) $\alpha_1 = 0.5$ and $\rho_0 = 0.4$ (c) $\alpha_1 = 1$ and $\rho_0 = 0.4$

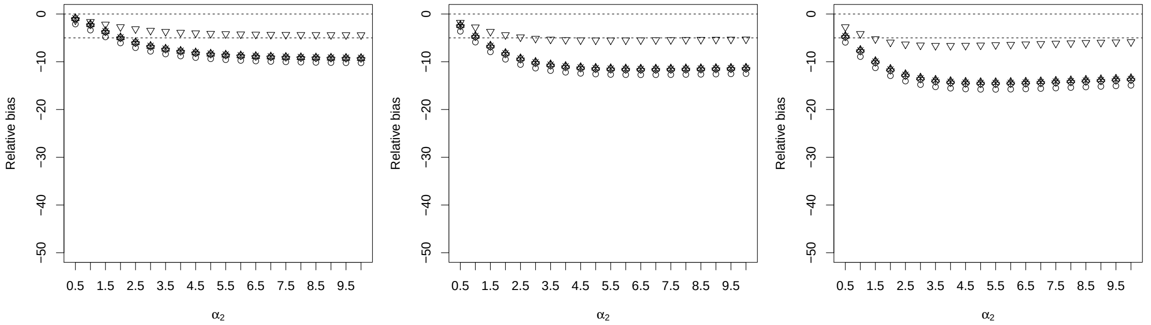

(d) $\alpha_1 = 0.1$ and $\rho_0 = 0.6$ (e) $\alpha_1 = 0.5$ and $\rho_0 = 0.6$ (f) $\alpha_1 = 1$ and $\rho_0 = 0.6$

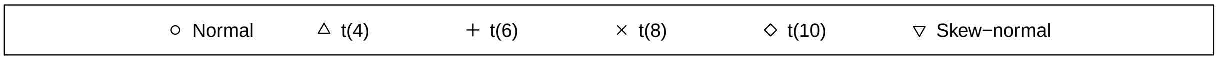

Figure 19: Relative bias (RB) of correlation estimates when the true underlying distribution is skew-t(6). The first ordinal variable has three categories and the second ordinal variable has five categories.

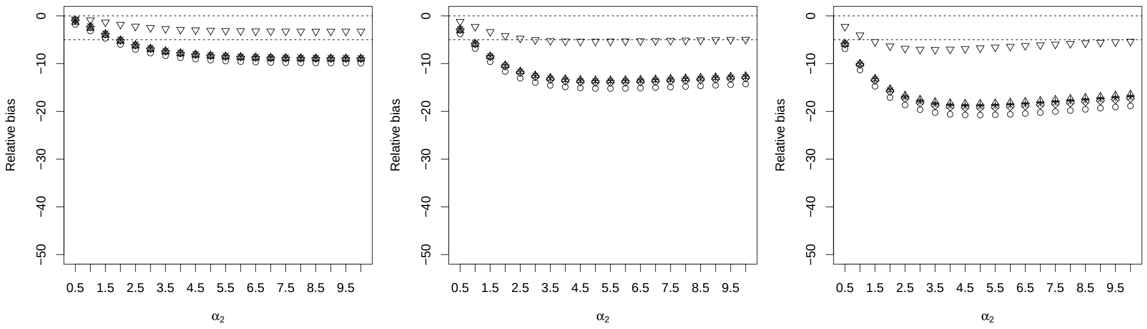

(a) $\alpha_1 = 0.1$ and $\rho_0 = 0.4$ (b) $\alpha_1 = 0.5$ and $\rho_0 = 0.4$ (c) $\alpha_1 = 1$ and $\rho_0 = 0.4$

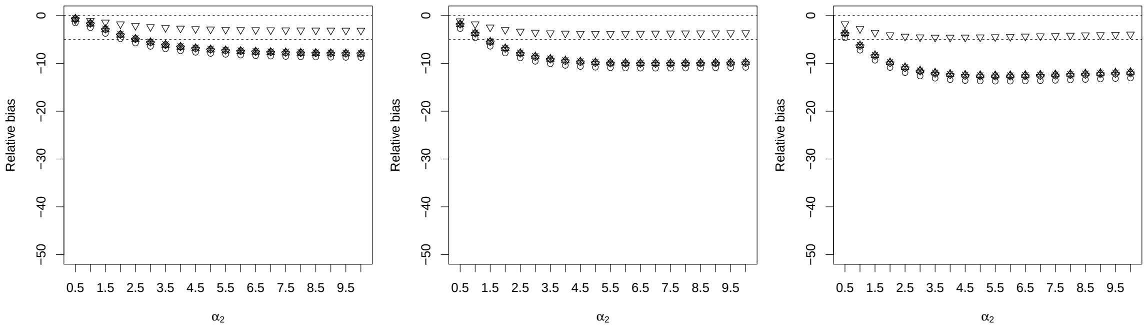

(d) $\alpha_1 = 0.1$ and $\rho_0 = 0.6$ (e) $\alpha_1 = 0.5$ and $\rho_0 = 0.6$ (f) $\alpha_1 = 1$ and $\rho_0 = 0.6$

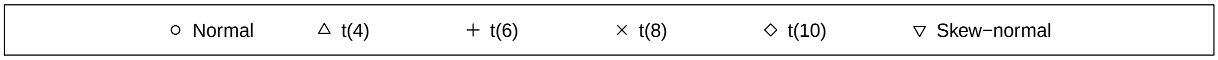

Figure 20: Relative bias (RB) of correlation estimates when the true underlying distribution is skew-t(8). The first ordinal variable has three categories and the second ordinal variable has five categories.

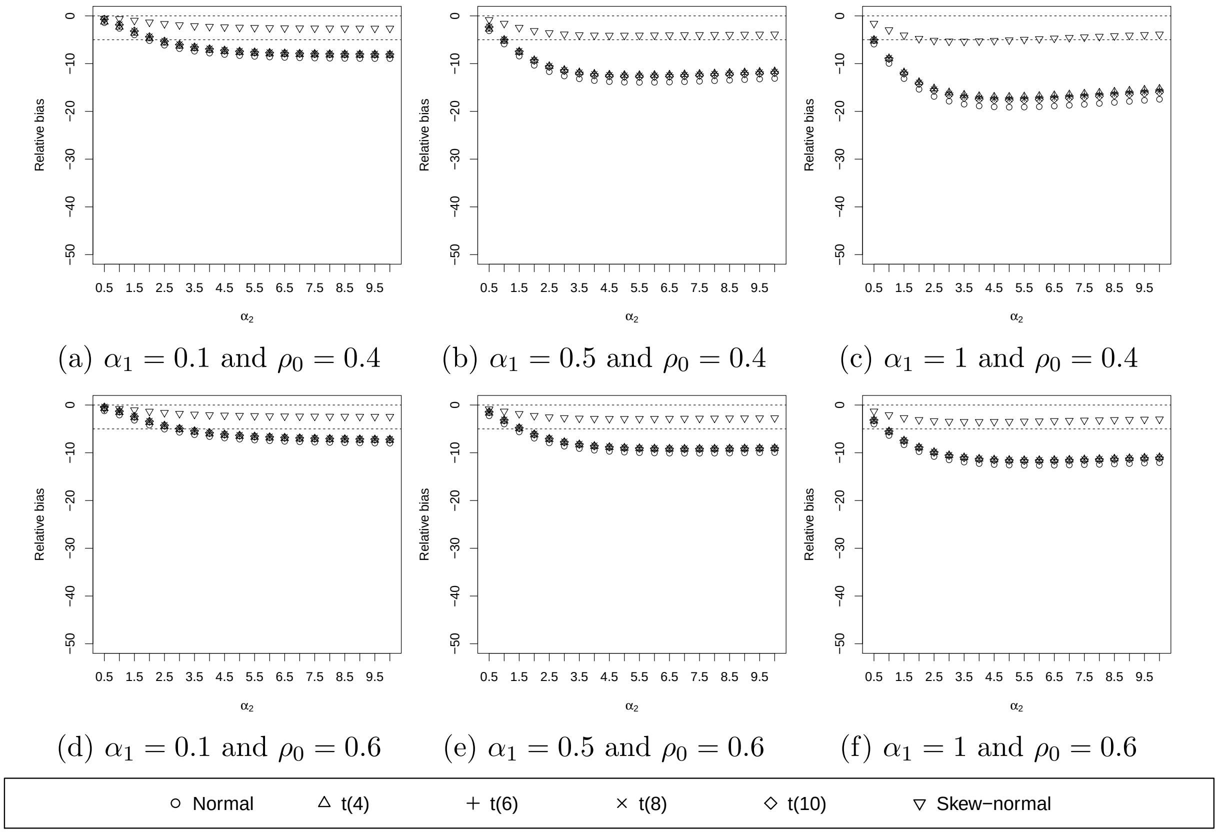

(a) $\alpha_1 = 0.1$ and $\rho_0 = 0.4$ (b) $\alpha_1 = 0.5$ and $\rho_0 = 0.4$ (c) $\alpha_1 = 1$ and $\rho_0 = 0.4$

(d) $\alpha_1 = 0.1$ and $\rho_0 = 0.6$ (e) $\alpha_1 = 0.5$ and $\rho_0 = 0.6$ (f) $\alpha_1 = 1$ and $\rho_0 = 0.6$

Figure 21: Relative bias (RB) of correlation estimates when the true underlying distribution is skew-t(10). The first ordinal variable has three categories and the second ordinal variable has five categories.

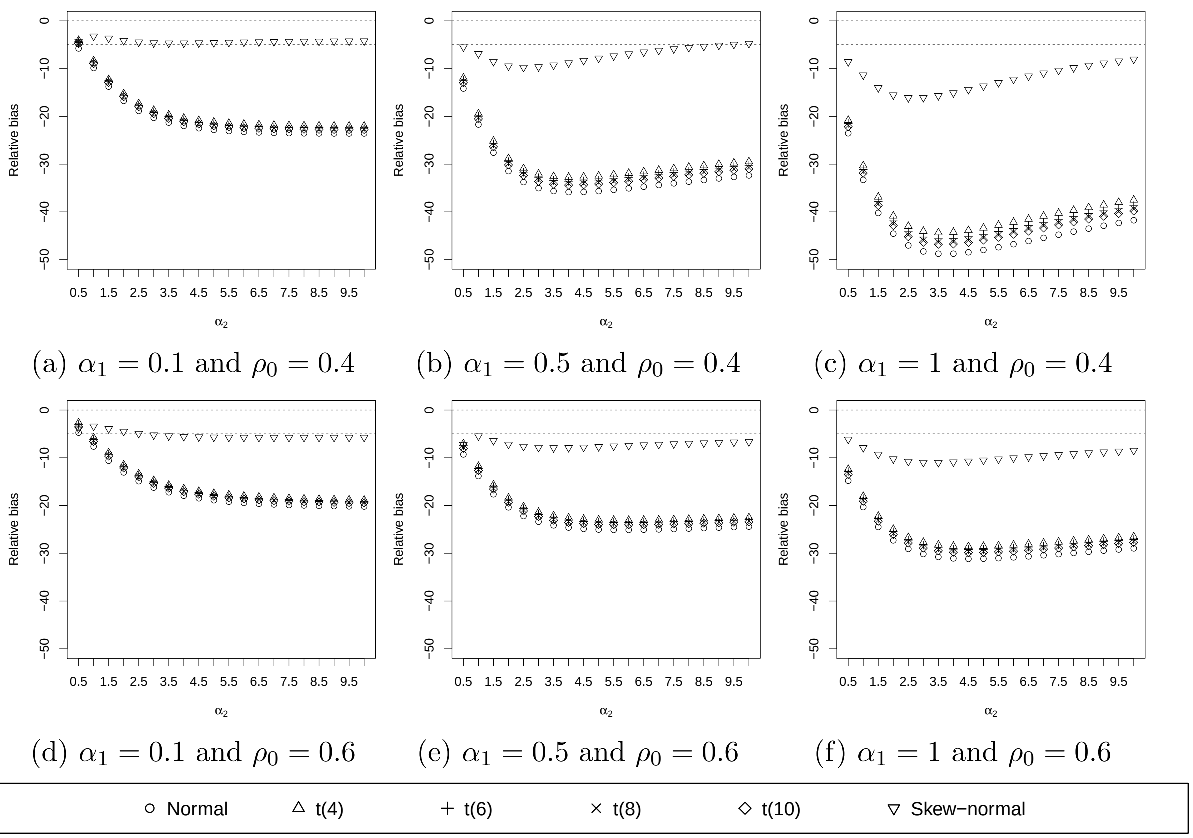

(a) $\alpha_1 = 0.1$ and $\rho_0 = 0.4$ (b) $\alpha_1 = 0.5$ and $\rho_0 = 0.4$ (c) $\alpha_1 = 1$ and $\rho_0 = 0.4$

(d) $\alpha_1 = 0.1$ and $\rho_0 = 0.6$ (e) $\alpha_1 = 0.5$ and $\rho_0 = 0.6$ (f) $\alpha_1 = 1$ and $\rho_0 = 0.6$

Figure 22: Relative bias (RB) of correlation estimates when the true underlying distribution is skew-t(4). Both ordinal variables have three categories.

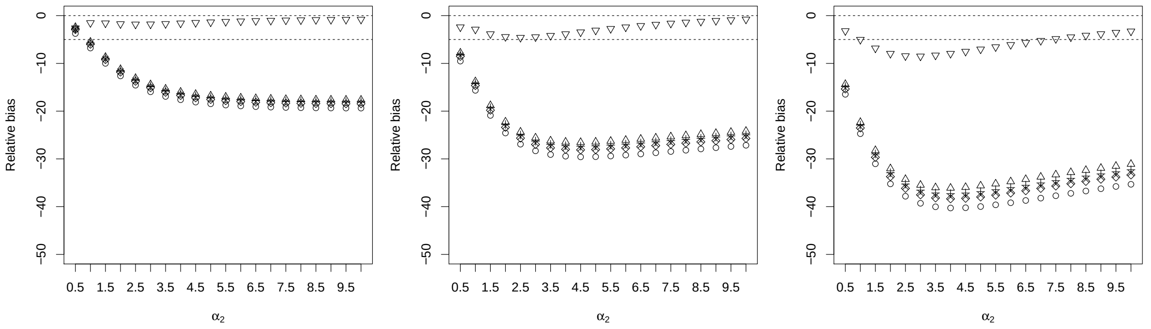

(a) $\alpha_1 = 0.1$ and $\rho_0 = 0.4$ (b) $\alpha_1 = 0.5$ and $\rho_0 = 0.4$ (c) $\alpha_1 = 1$ and $\rho_0 = 0.4$

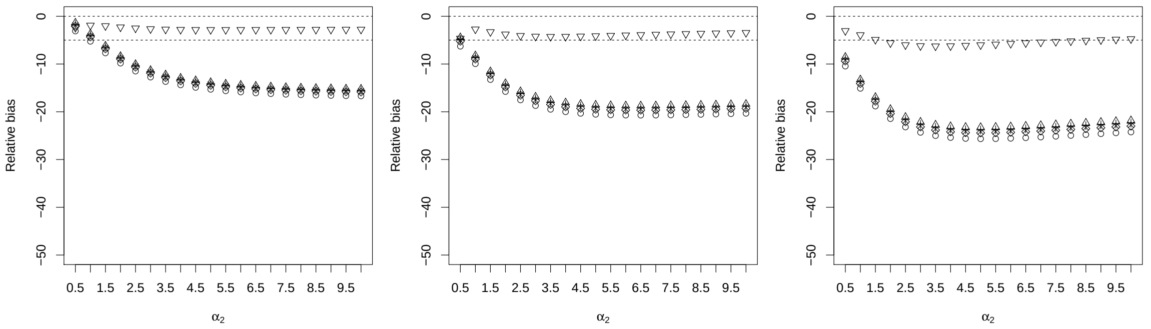

(d) $\alpha_1 = 0.1$ and $\rho_0 = 0.6$ (e) $\alpha_1 = 0.5$ and $\rho_0 = 0.6$ (f) $\alpha_1 = 1$ and $\rho_0 = 0.6$

○ Normal △ t(4) + t(6) × t(8) ◇ t(10) ▽ Skew−normal

Figure 23: Relative bias (RB) of correlation estimates when the true underlying distribution is skew-t(6). Both ordinal variables have three categories.

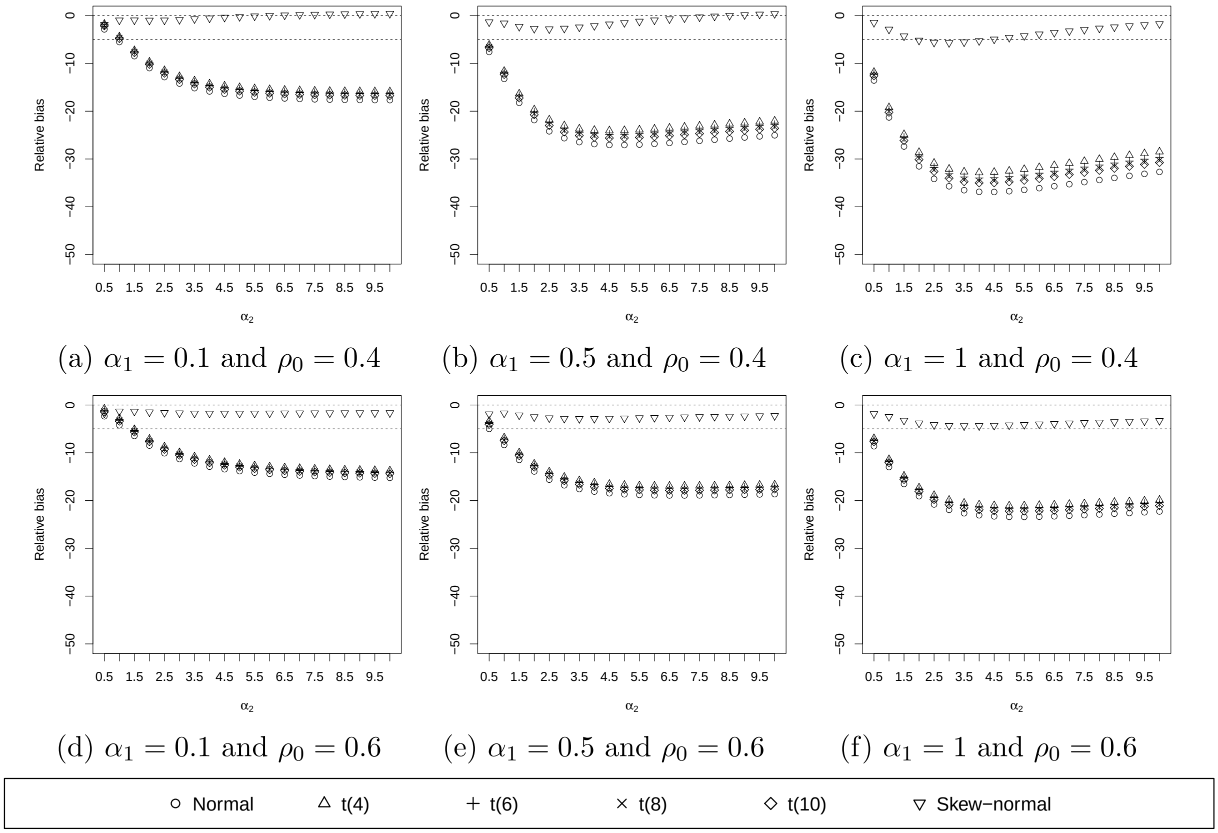

Figure 24: Relative bias (RB) of correlation estimates when the true underlying distribution is skew-t(8). Both ordinal variables have three categories.

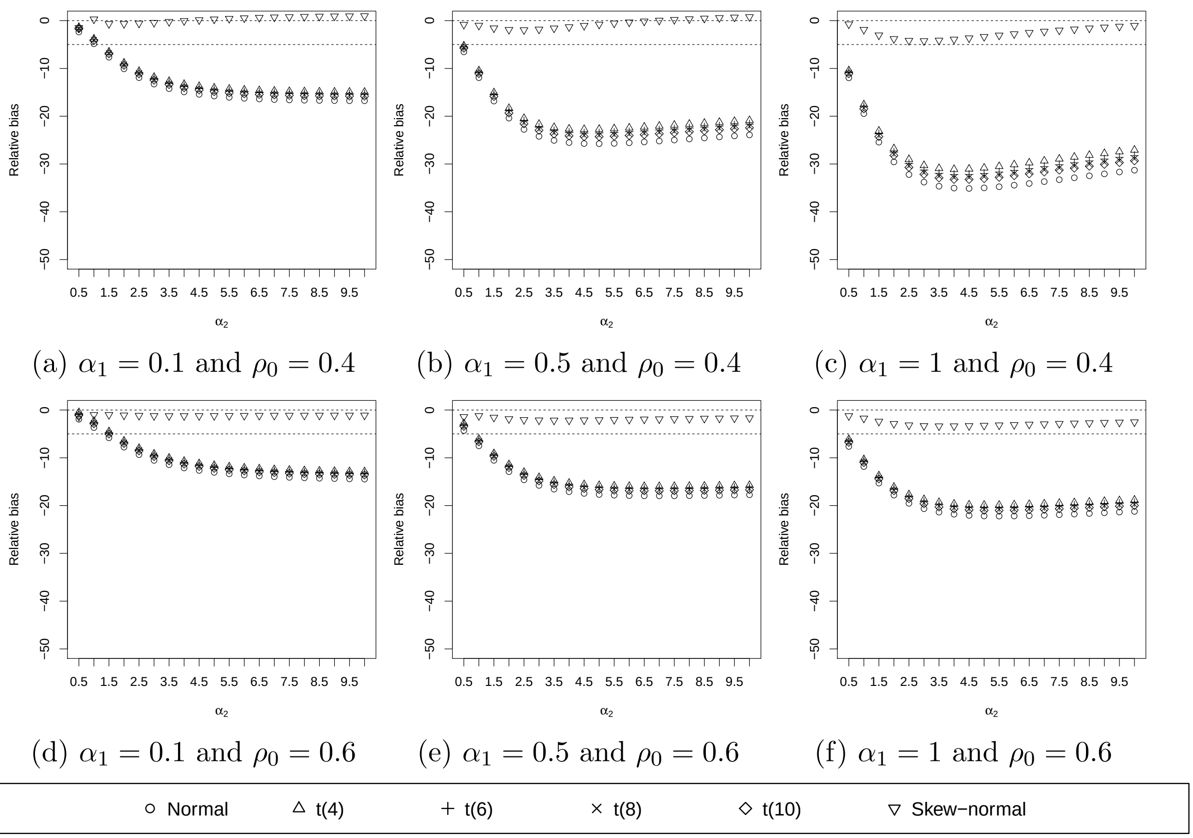

(a) $\alpha_1 = 0.1$ and $\rho_0 = 0.4$ (b) $\alpha_1 = 0.5$ and $\rho_0 = 0.4$ (c) $\alpha_1 = 1$ and $\rho_0 = 0.4$

(d) $\alpha_1 = 0.1$ and $\rho_0 = 0.6$ (e) $\alpha_1 = 0.5$ and $\rho_0 = 0.6$ (f) $\alpha_1 = 1$ and $\rho_0 = 0.6$

Figure 25: Relative bias (RB) of correlation estimates when the true underlying distribution is skew-t(10). Both ordinal variables have three categories.

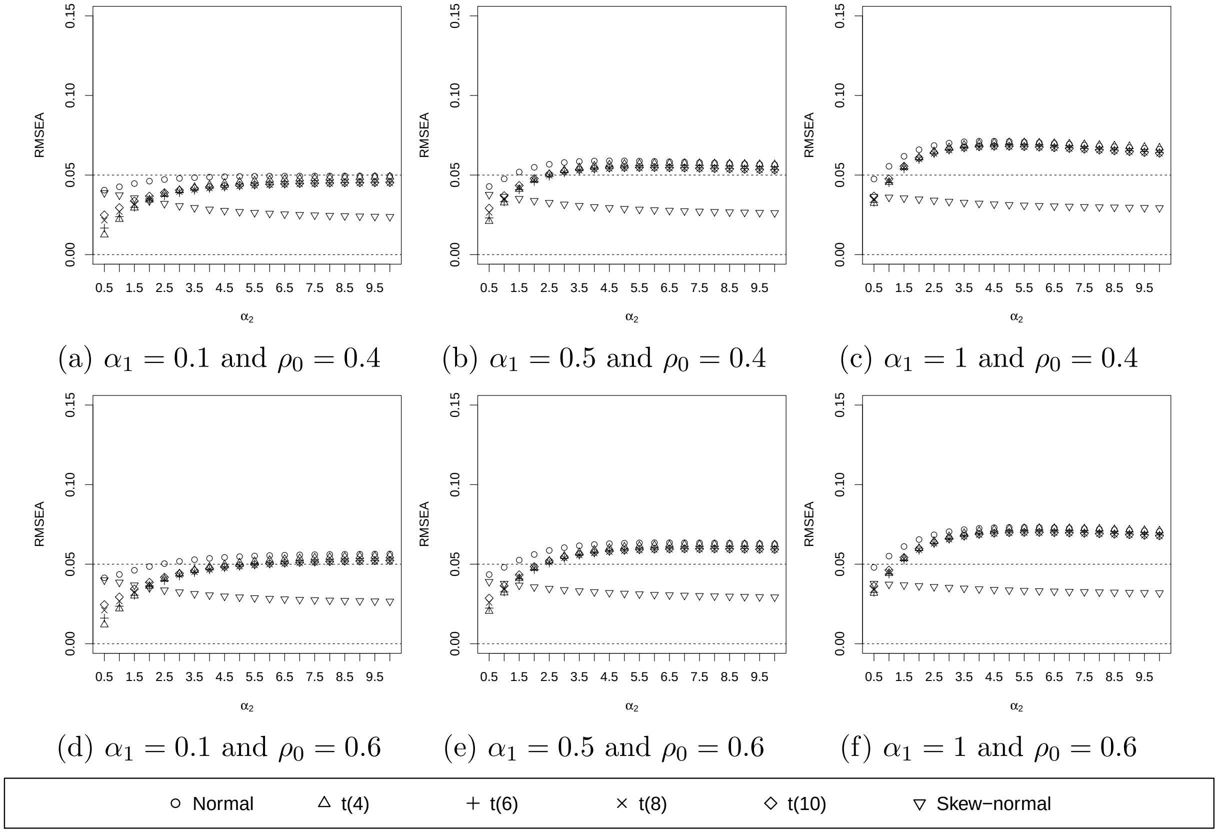

(a) $\alpha_1 = 0.1$ and $\rho_0 = 0.4$ (b) $\alpha_1 = 0.5$ and $\rho_0 = 0.4$ (c) $\alpha_1 = 1$ and $\rho_0 = 0.4$

(d) $\alpha_1 = 0.1$ and $\rho_0 = 0.6$ (e) $\alpha_1 = 0.5$ and $\rho_0 = 0.6$ (f) $\alpha_1 = 1$ and $\rho_0 = 0.6$

Figure 26: Root mean squared error of approximatoin (RMSEA) of correlation estimates when the true underlying distribution is skew-t(4). Both ordinal variables have five categories.

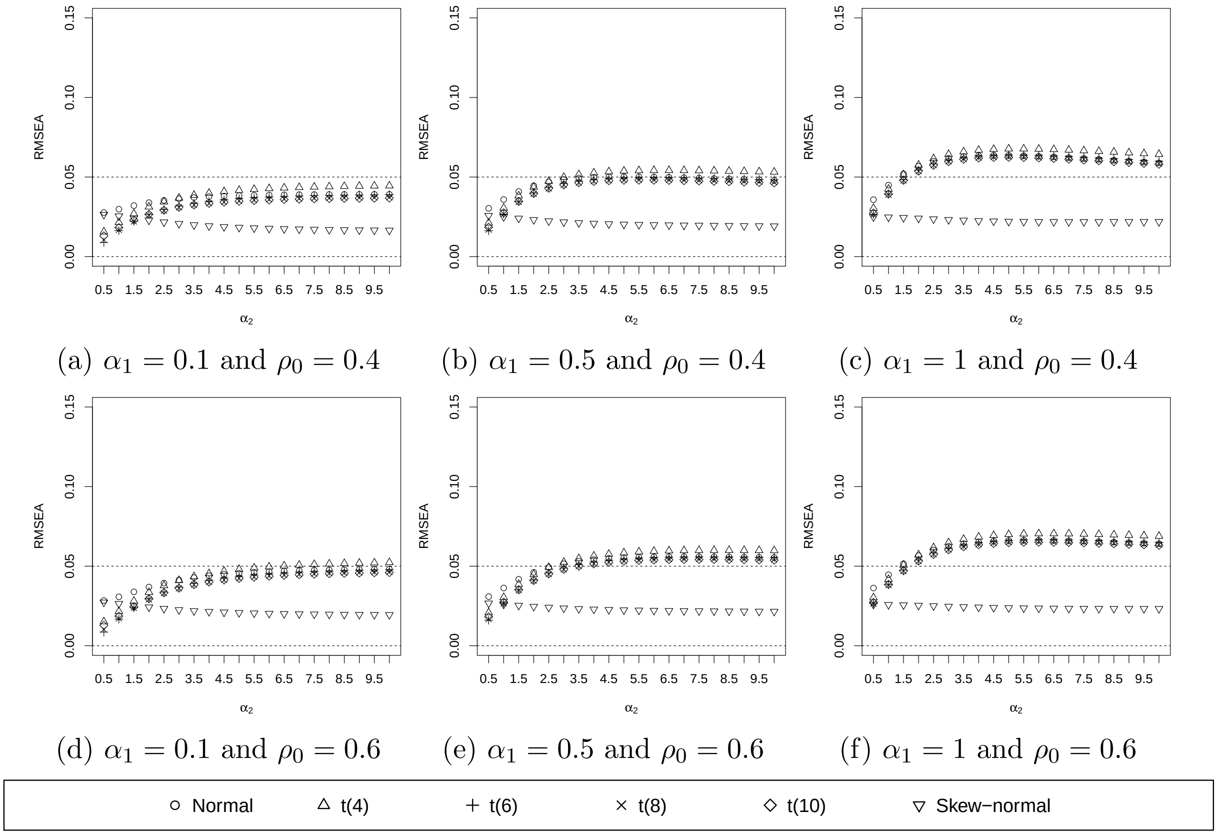

(a) $\alpha_1 = 0.1$ and $\rho_0 = 0.4$ (b) $\alpha_1 = 0.5$ and $\rho_0 = 0.4$ (c) $\alpha_1 = 1$ and $\rho_0 = 0.4$

(d) $\alpha_1 = 0.1$ and $\rho_0 = 0.6$ (e) $\alpha_1 = 0.5$ and $\rho_0 = 0.6$ (f) $\alpha_1 = 1$ and $\rho_0 = 0.6$

Figure 27: Root mean squared error of approximatoin (RMSEA) of correlation estimates when the true underlying distribution is skew-t(6). Both ordinal variables have five categories.

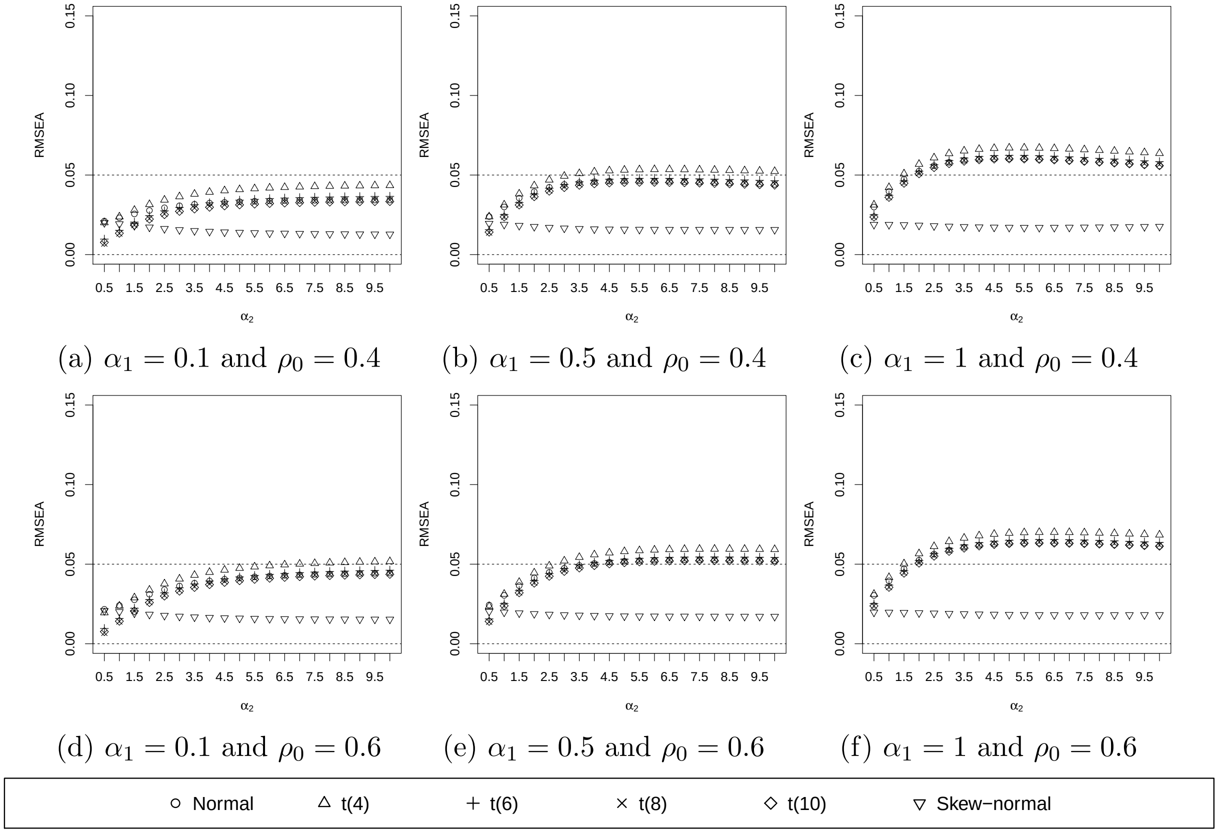

Figure 28: Root mean squared error of approximatoin (RMSEA) of correlation estimates when the true underlying distribution is skew-t(8). Both ordinal variables have five categories.

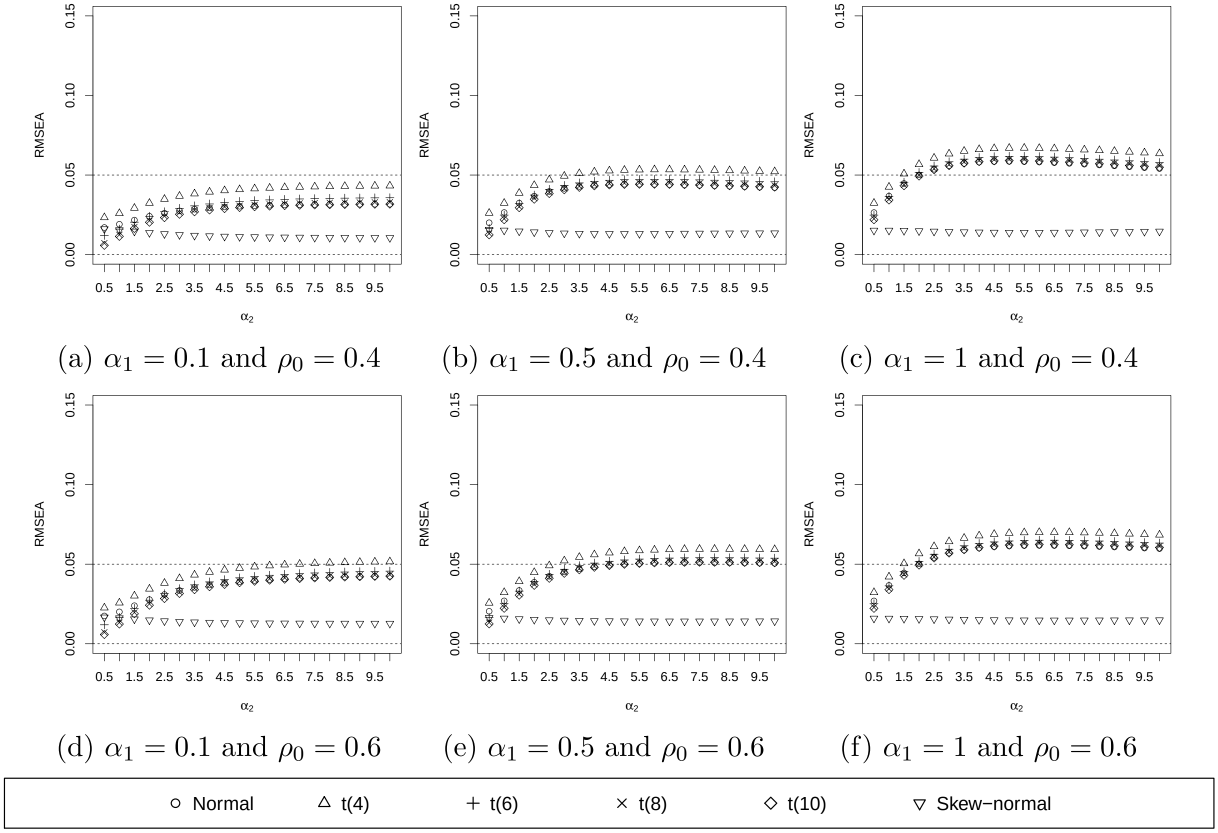

(a) $\alpha_1 = 0.1$ and $\rho_0 = 0.4$ (b) $\alpha_1 = 0.5$ and $\rho_0 = 0.4$ (c) $\alpha_1 = 1$ and $\rho_0 = 0.4$

(d) $\alpha_1 = 0.1$ and $\rho_0 = 0.6$ (e) $\alpha_1 = 0.5$ and $\rho_0 = 0.6$ (f) $\alpha_1 = 1$ and $\rho_0 = 0.6$

Figure 29: Root mean squared error of approximatoin (RMSEA) of correlation estimates when the true underlying distribution is skew-t(10). Both ordinal variables have five categories.

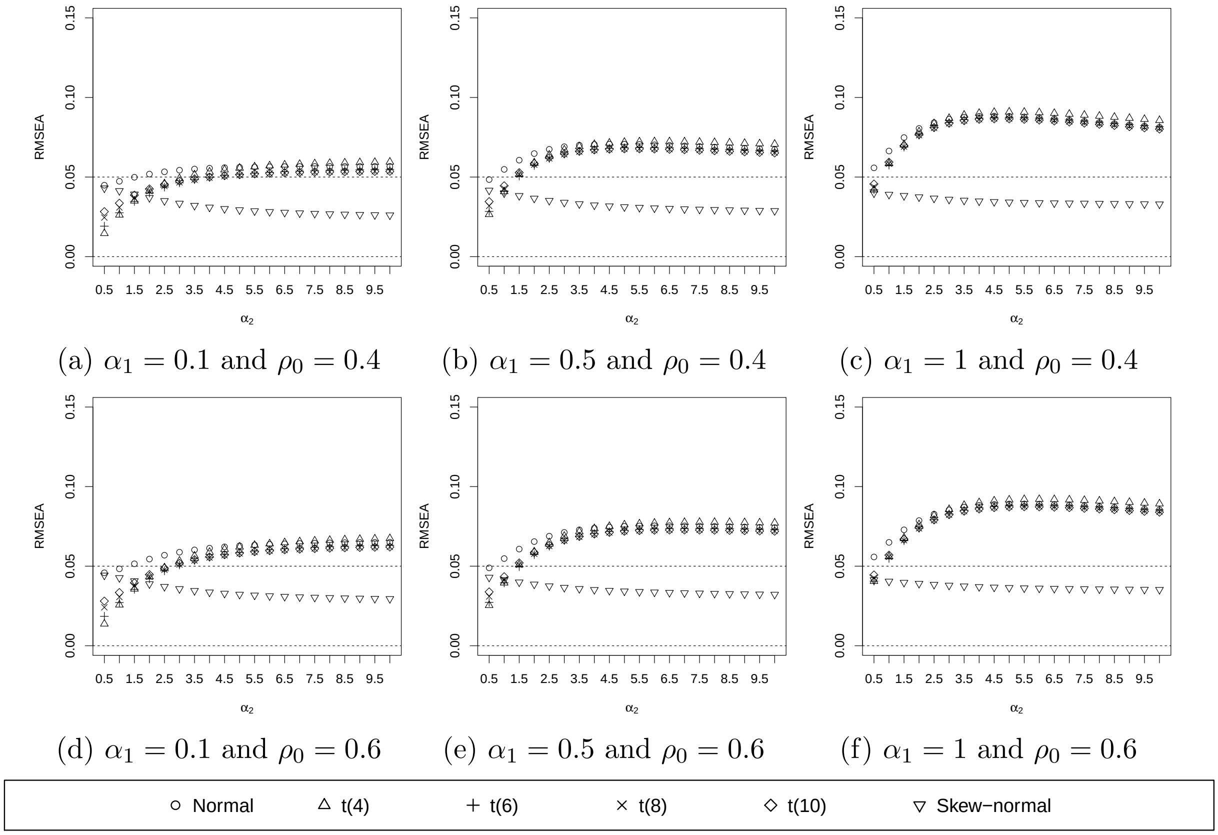

(a) $\alpha_1 = 0.1$ and $\rho_0 = 0.4$ (b) $\alpha_1 = 0.5$ and $\rho_0 = 0.4$ (c) $\alpha_1 = 1$ and $\rho_0 = 0.4$

(d) $\alpha_1 = 0.1$ and $\rho_0 = 0.6$ (e) $\alpha_1 = 0.5$ and $\rho_0 = 0.6$ (f) $\alpha_1 = 1$ and $\rho_0 = 0.6$

Figure 30: Root mean squared error of approximatoin (RMSEA) of correlation estimates when the true underlying distribution is skew-t(4). The first ordinal variable has three categories and the second ordinal variable has five categories.

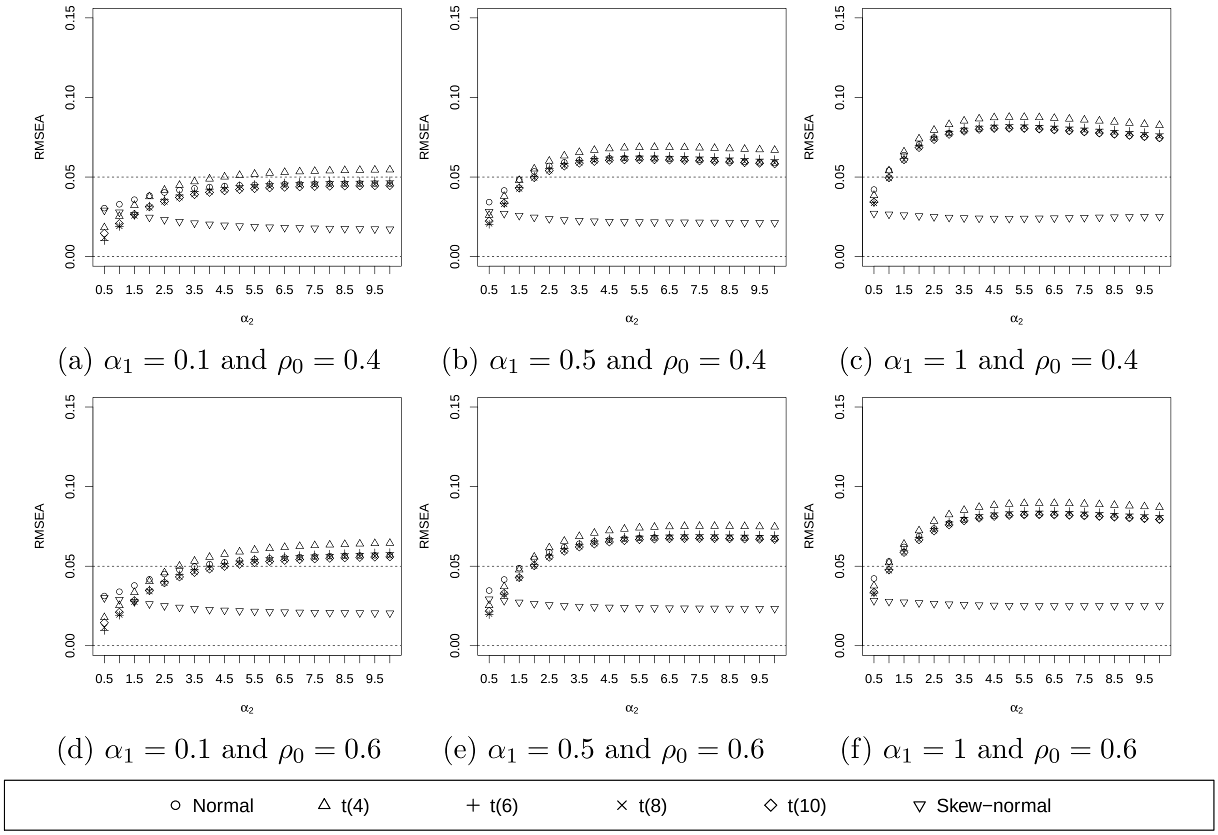

(a) $\alpha_1 = 0.1$ and $\rho_0 = 0.4$ (b) $\alpha_1 = 0.5$ and $\rho_0 = 0.4$ (c) $\alpha_1 = 1$ and $\rho_0 = 0.4$

(d) $\alpha_1 = 0.1$ and $\rho_0 = 0.6$ (e) $\alpha_1 = 0.5$ and $\rho_0 = 0.6$ (f) $\alpha_1 = 1$ and $\rho_0 = 0.6$

Figure 31: Root mean squared error of approximatoin (RMSEA) of correlation estimates when the true underlying distribution is skew-t(6). The first ordinal variable has three categories and the second ordinal variable has five categories.

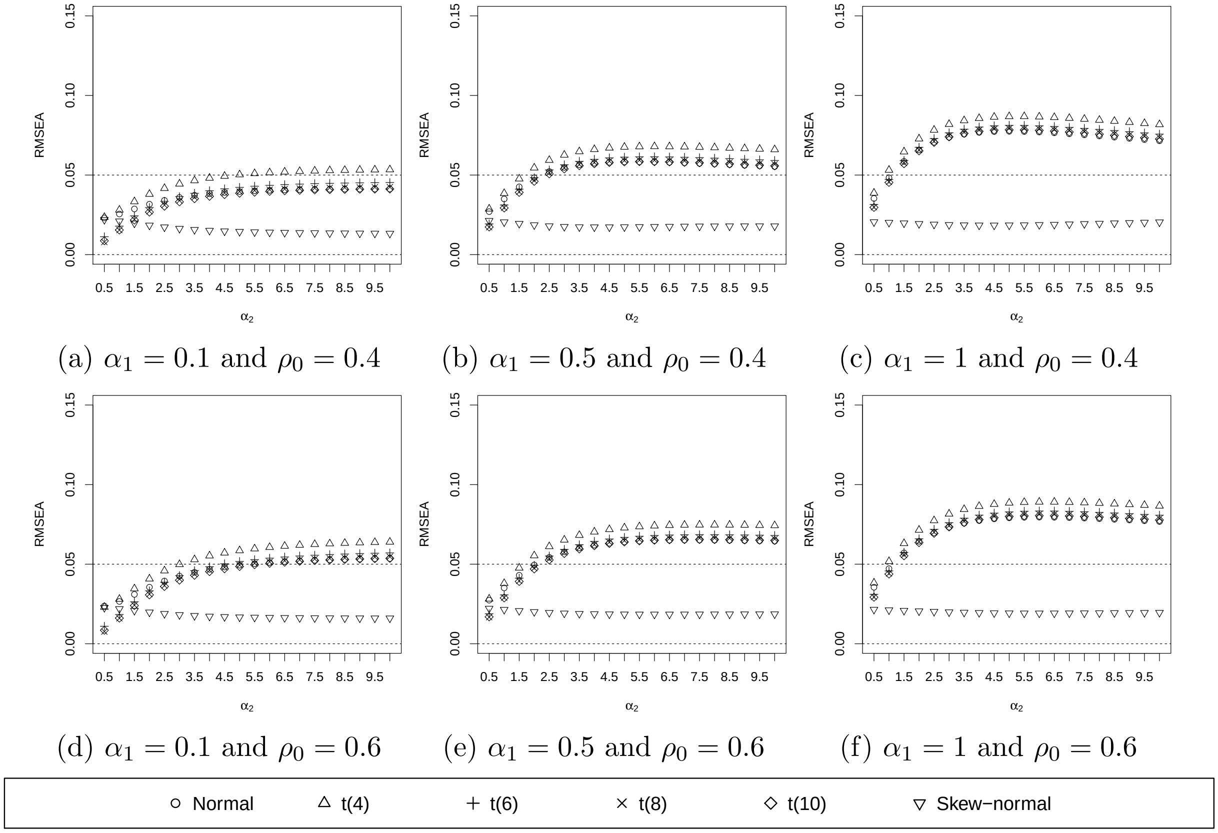

(a) $\alpha_1 = 0.1$ and $\rho_0 = 0.4$ (b) $\alpha_1 = 0.5$ and $\rho_0 = 0.4$ (c) $\alpha_1 = 1$ and $\rho_0 = 0.4$

(d) $\alpha_1 = 0.1$ and $\rho_0 = 0.6$ (e) $\alpha_1 = 0.5$ and $\rho_0 = 0.6$ (f) $\alpha_1 = 1$ and $\rho_0 = 0.6$

Figure 32: Root mean squared error of approximatoin (RMSEA) of correlation estimates when the true underlying distribution is skew-t(8). The first ordinal variable has three categories and the second ordinal variable has five categories.

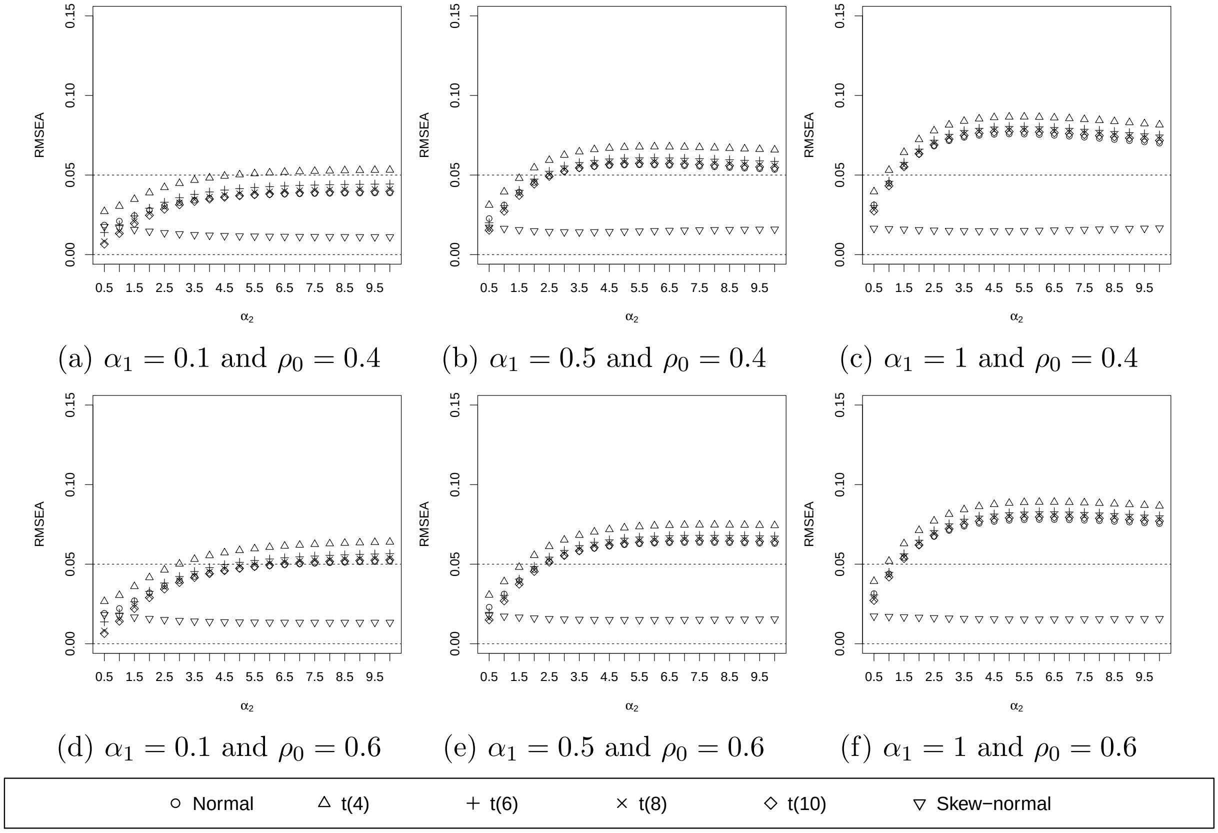

(a) $\alpha_1 = 0.1$ and $\rho_0 = 0.4$ (b) $\alpha_1 = 0.5$ and $\rho_0 = 0.4$ (c) $\alpha_1 = 1$ and $\rho_0 = 0.4$

(d) $\alpha_1 = 0.1$ and $\rho_0 = 0.6$ (e) $\alpha_1 = 0.5$ and $\rho_0 = 0.6$ (f) $\alpha_1 = 1$ and $\rho_0 = 0.6$

Figure 33: Root mean squared error of approximatoin (RMSEA) of correlation estimates when the true underlying distribution is skew-t(10). The first ordinal variable has three categories and the second ordinal variable has five categories.

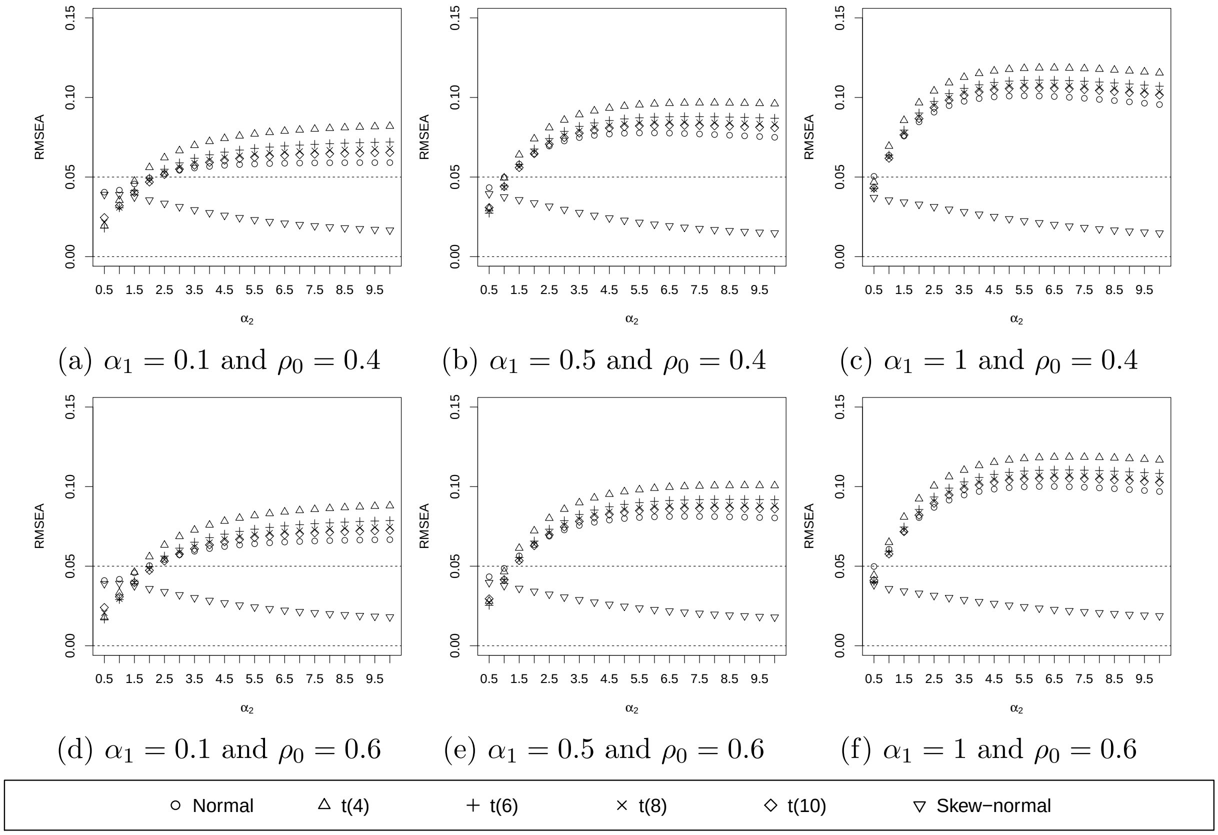

(a) $\alpha_1 = 0.1$ and $\rho_0 = 0.4$ (b) $\alpha_1 = 0.5$ and $\rho_0 = 0.4$ (c) $\alpha_1 = 1$ and $\rho_0 = 0.4$

(d) $\alpha_1 = 0.1$ and $\rho_0 = 0.6$ (e) $\alpha_1 = 0.5$ and $\rho_0 = 0.6$ (f) $\alpha_1 = 1$ and $\rho_0 = 0.6$

Figure 34: Root mean squared error of approximatoin (RMSEA) of correlation estimates when the true underlying distribution is skew-t(4). Both ordinal variables have three categories.

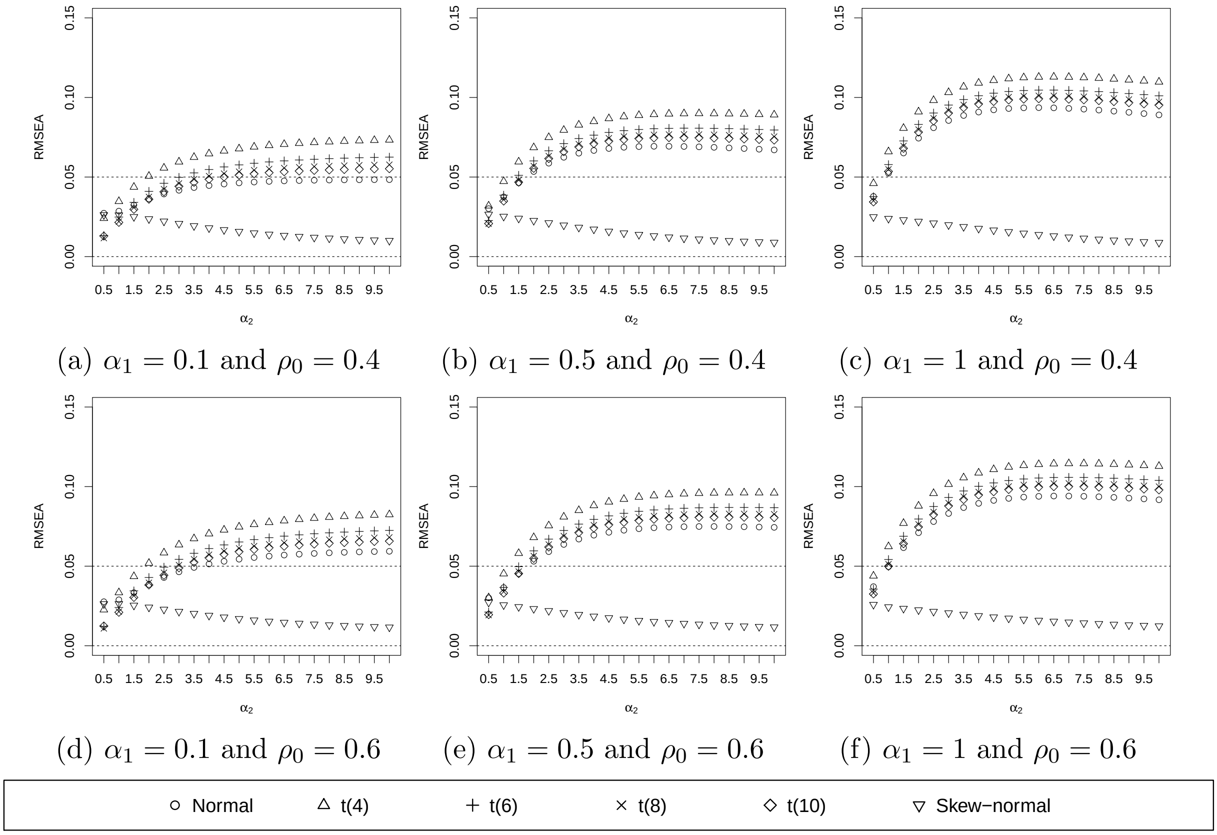

(a) $\alpha_1 = 0.1$ and $\rho_0 = 0.4$ (b) $\alpha_1 = 0.5$ and $\rho_0 = 0.4$ (c) $\alpha_1 = 1$ and $\rho_0 = 0.4$

(d) $\alpha_1 = 0.1$ and $\rho_0 = 0.6$ (e) $\alpha_1 = 0.5$ and $\rho_0 = 0.6$ (f) $\alpha_1 = 1$ and $\rho_0 = 0.6$

Figure 35: Root mean squared error of approximatoin (RMSEA) of correlation estimates when the true underlying distribution is skew-t(6). Both ordinal variables have three categories.

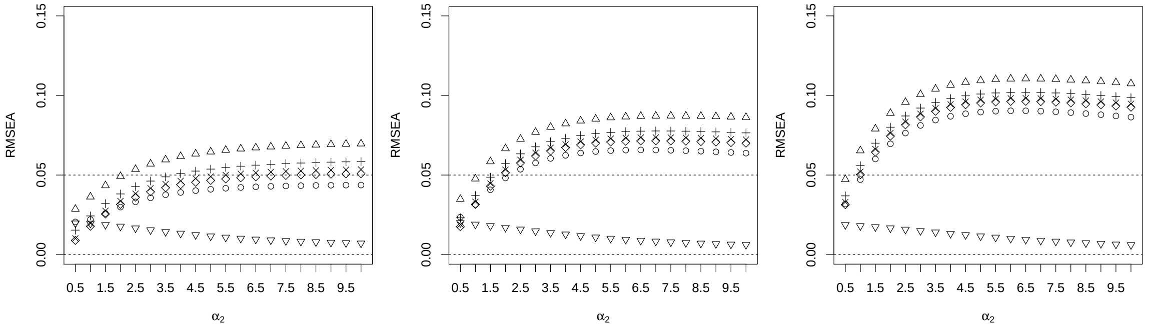

(a) $\alpha_1 = 0.1$ and $\rho_0 = 0.4$ (b) $\alpha_1 = 0.5$ and $\rho_0 = 0.4$ (c) $\alpha_1 = 1$ and $\rho_0 = 0.4$

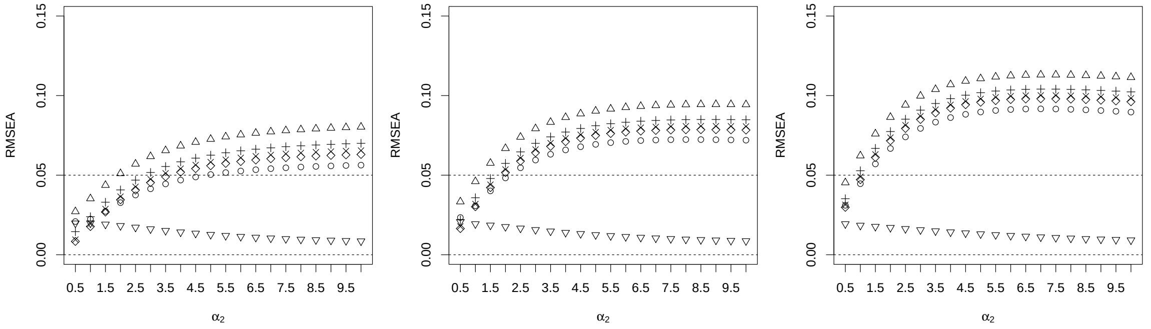

(d) $\alpha_1 = 0.1$ and $\rho_0 = 0.6$ (e) $\alpha_1 = 0.5$ and $\rho_0 = 0.6$ (f) $\alpha_1 = 1$ and $\rho_0 = 0.6$

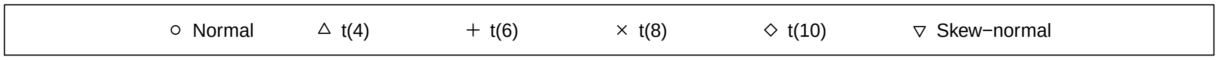

Figure 36: Root mean squared error of approximatoin (RMSEA) of correlation estimates when the true underlying distribution is skew-t(8). Both ordinal variables have three categories.

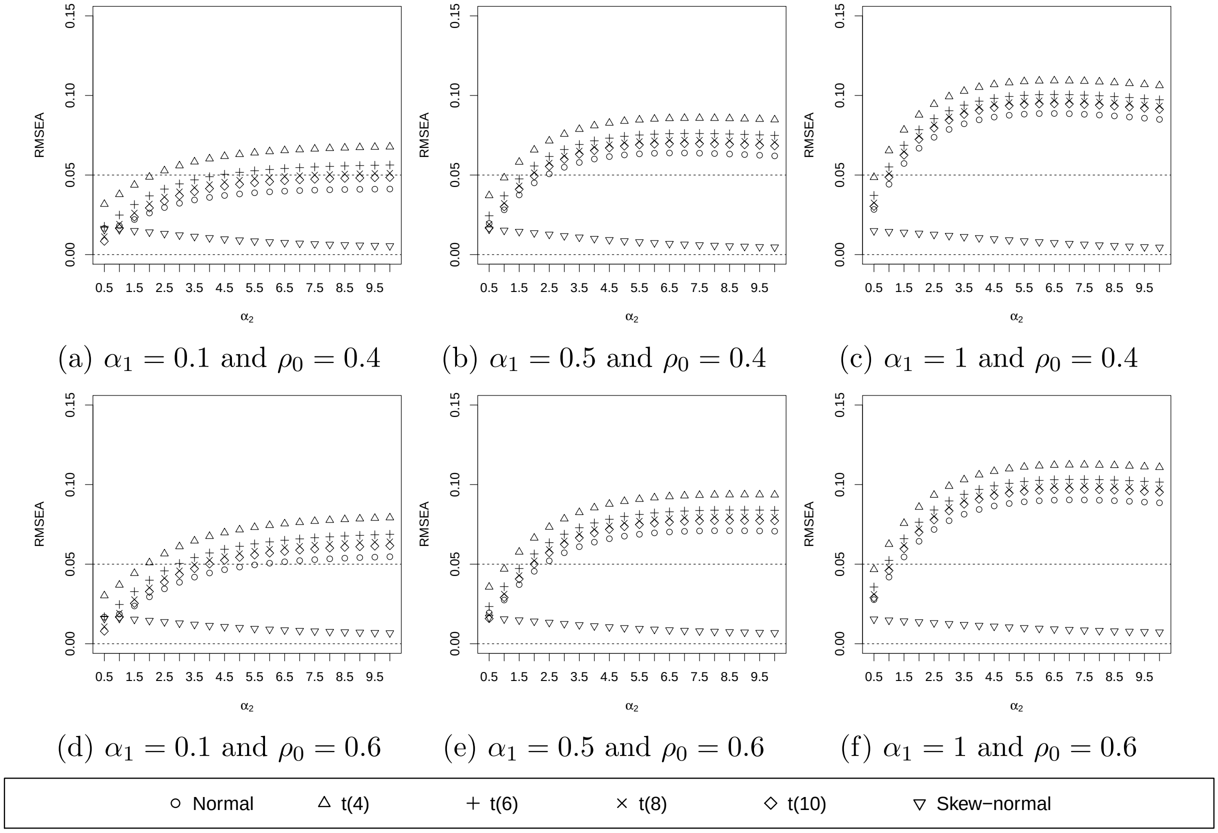

Figure 37: Root mean squared error of approximatoin (RMSEA) of correlation estimates when the true underlying distribution is skew-t(10). Both ordinal variables have three categories.

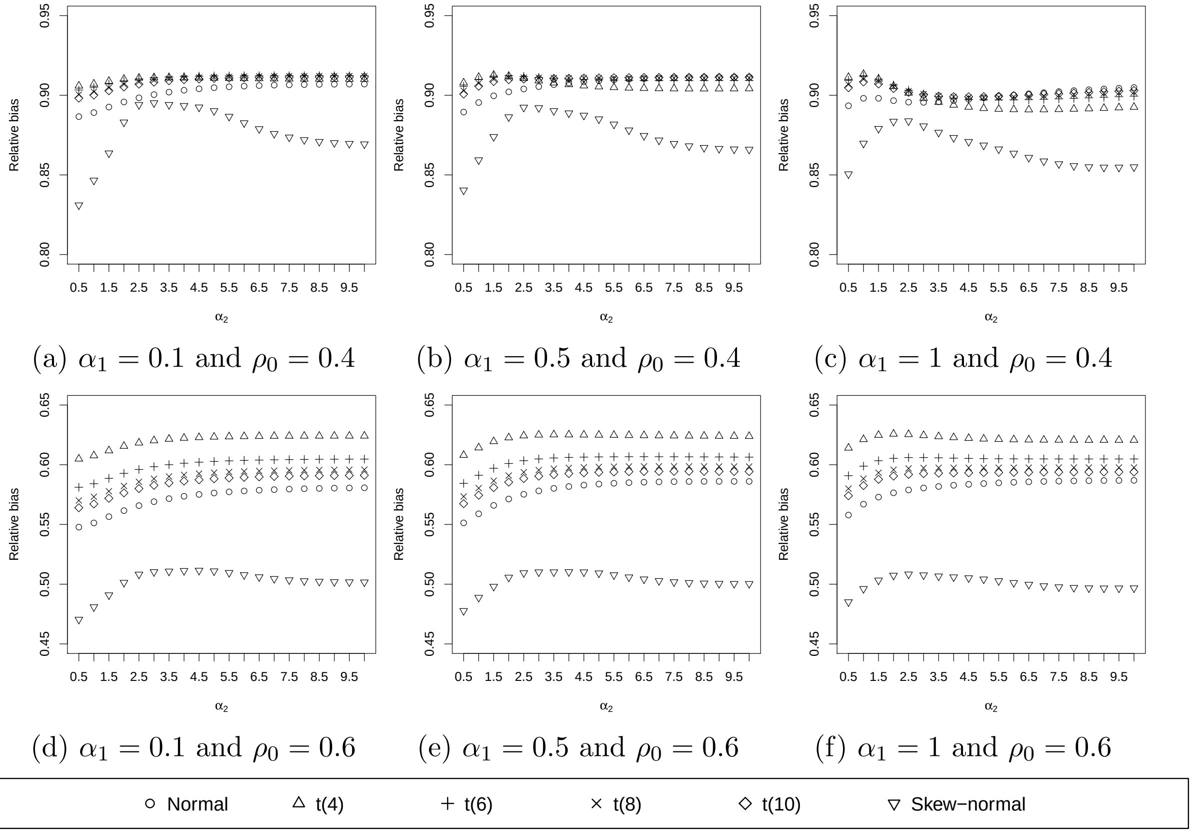

(a) $\alpha_1 = 0.1$ and $\rho_0 = 0.4$ (b) $\alpha_1 = 0.5$ and $\rho_0 = 0.4$ (c) $\alpha_1 = 1$ and $\rho_0 = 0.4$

(d) $\alpha_1 = 0.1$ and $\rho_0 = 0.6$ (e) $\alpha_1 = 0.5$ and $\rho_0 = 0.6$ (f) $\alpha_1 = 1$ and $\rho_0 = 0.6$

Figure 38: Asymptotic variances of correlation estimators when the true underlying distribution is skew-normal. Both ordinal variables have five categories.

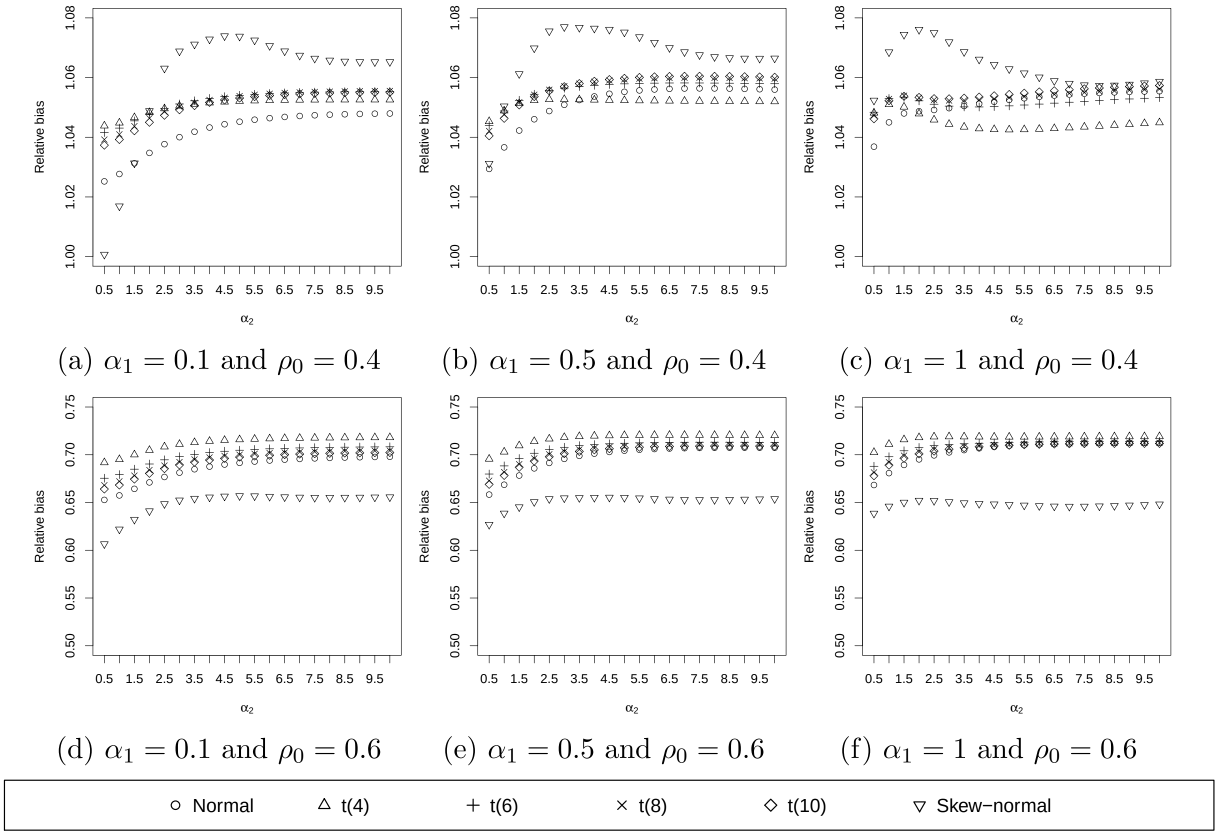

Figure 39: Asymptotic variances of correlation estimators when the true underlying distribution is skew-normal. The first ordinal variable has three categories and the second ordinal variable has five categories.

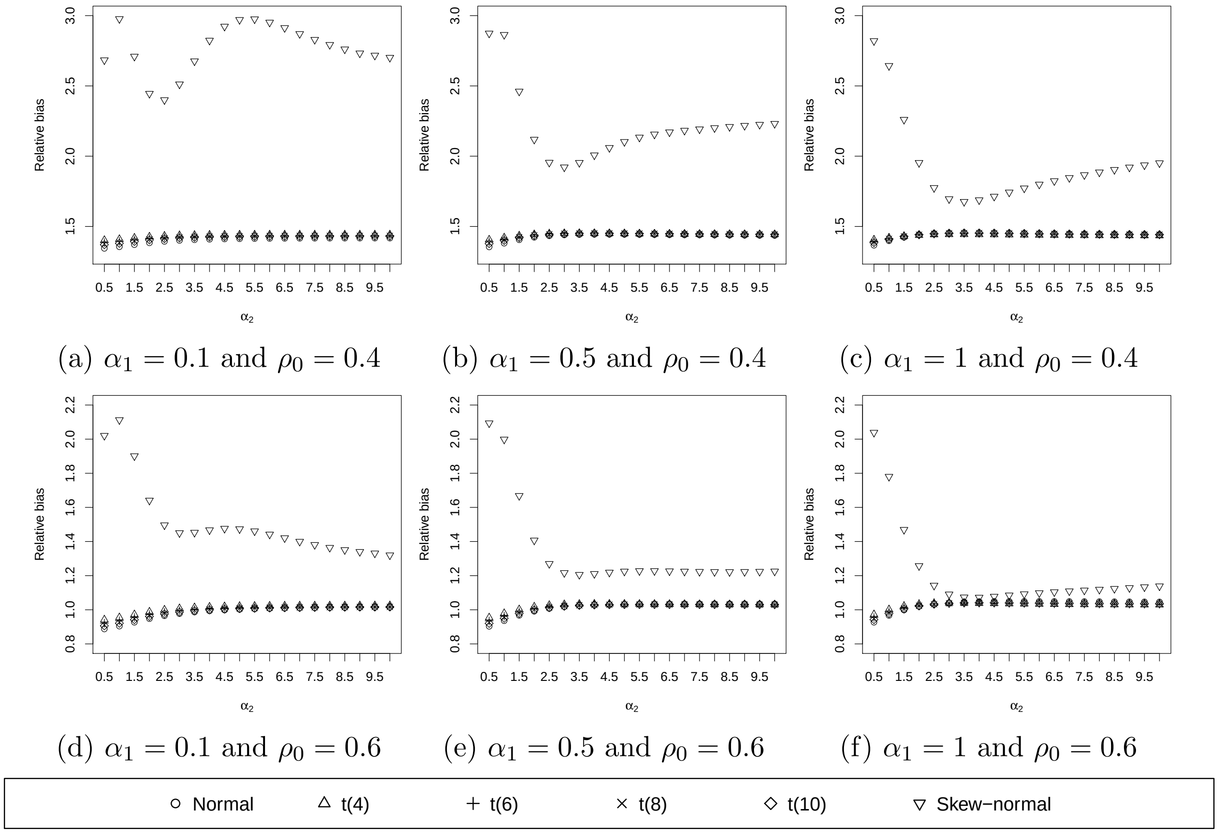

(a) $\alpha_1 = 0.1$ and $\rho_0 = 0.4$ (b) $\alpha_1 = 0.5$ and $\rho_0 = 0.4$ (c) $\alpha_1 = 1$ and $\rho_0 = 0.4$

(d) $\alpha_1 = 0.1$ and $\rho_0 = 0.6$ (e) $\alpha_1 = 0.5$ and $\rho_0 = 0.6$ (f) $\alpha_1 = 1$ and $\rho_0 = 0.6$

Figure 40: Asymptotic variances of correlation estimators when the true underlying distribution is skew-normal. Both ordinal variables have three categories.
